# Synthesis of Trifluoroacetamidoketones by Acylation of Ferrocene with In Situ Protected Amino Acids

**DOI:** 10.1021/acs.joc.4c02717

**Published:** 2025-02-18

**Authors:** Michał Piotrowicz, Natasza Masłowska, Róża Dziewiątkowska, Anna Makal, Bogna Rudolf

**Affiliations:** † University of Lodz, Faculty of Chemistry, Department of Organic Chemistry, Tamka 12, 91-403 Lodz, Poland; ‡ Biological and Chemical Research Centre, Faculty of Chemistry, University of Warsaw, Żwirki I Wigury 101, 02-089 Warszawa, Poland

## Abstract

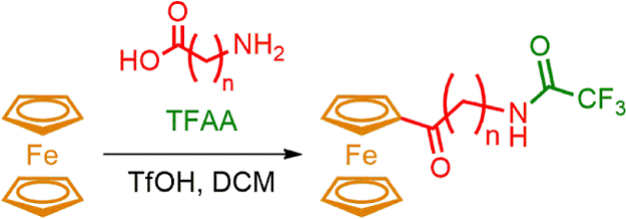

The Friedel–Crafts acylation of ferrocene with amino acids carried out under mild conditions (metal-free catalytic system, room temperature, and a short reaction time of 1 h) has been reported. The acylating agent is generated in situ by N-protection of the amino group of the amino acid, followed by formation of mixed anhydride. This one-pot triflic-acid-promoted reaction provides *N*-trifluoroacetyl-protected amidoketones in good to excellent yields. Moreover, the trifluoroacetyl group can be easily removed or replaced with another protecting group under mild conditions in a one-pot procedure.

## 1 Introduction

Aminoketones constitute an important group of bifunctional organic compounds.^1,2^ Representatives of this class of compounds have various applications. As the compounds possess biological activity, they are utilized in medicinal chemistry as drugs, active substances, and their precursors.^3−12^

For example, α-aminoketones, exhibiting a wide spectrum of regulatory properties, serve as the basis for antidepressant drugs (Bupropion),^13^ appetite suppressants (Amfepramone),^14^ ACE inhibitors for hypertension treatment (Keto-ACE),^15,16^ or antiplatelet agents (Prasugrel).^17^ Similarly, the β-aminoketone skeleton can be found in Proroxane (nonselective α-adrenergic blocker),^18,19^ Tolperisone (vasodilation),^20^ Oxyfedrine (coronary artery disease),^21^ or Sitagliptin (antidiabetic).^22^

On the other hand, the presence of both carbonyl and amino groups in their molecular structure makes them extremely useful building blocks employed in organic synthesis.^23,24^ As building blocks, α-aminoketones play an important role in the synthesis of heterocycles.^25−28^ Particularly significant is their role in the formation of nitrogen-containing ring systems such as pyrazines^29,30^ or pyrroles.^31,32^ Through the reduction of the carbonyl group, α-aminoketones yield 1,2-amino alcohols,^33−35^ which are useful ligands and chiral auxiliaries.^36^ In turn, β-aminoketones find application in the synthesis of 1,3-amino alcohols,^37−42^ 1,3-diamines,^43−45^ and γ-aryl amines^46−49^ and the asymmetric synthesis of β-amino acids.^50,51^ In the Mannich reaction, β-aminoketones can undergo cyclization with aldehydes to form piperidones, which constitutes an important aspect of the asymmetric synthesis of these compounds.^52^

The importance of this group of compounds, also as useful synthetic intermediates, has led to the development of various synthesis methods for aminoketones. Mainly, these include the addition reactions of organolithium and organomagnesium compounds to α-amino acids^53,54^ and their derivatives such as esters^55^ or Weinreb amides^56^ or the direct amination of ketones in the presence of oxidants for the synthesis of α-aminoketones.^57^ For β-aminoketones, these are mainly various variants of Mannich reactions.^2^ Most of these reactions require, due to the presence of two active groups in aminoketone molecules, the use of blocking groups, the use of transition metal catalysis, or the use of organocatalysts with complex structures.^1,2^

This prompted us to develop a simple one-step synthesis enabling the production of aromatics α, β, γ, etc., aminoketones from aromatic compounds and unprotected amino acids through electrophilic substitution in the aromatic system. There are few known examples in the literature of using α-amino acid derivatives in the Friedel–Crafts intra- and intermolecular reactions with benzene and its derivatives.^58−65^ However, they require α-amino acid chlorides with blocked amino groups, which are unstable, and their synthesis requires a two-step process.

Herein, we report the efficient acylation of ferrocene with unprotected amino acids and trifluoroacetic anhydride (TFAA) which undergoes under mild conditions in the presence of triflic acid.

## 2 Results and Discussion

### 2.1 Synthesis

**Scheme 1:**
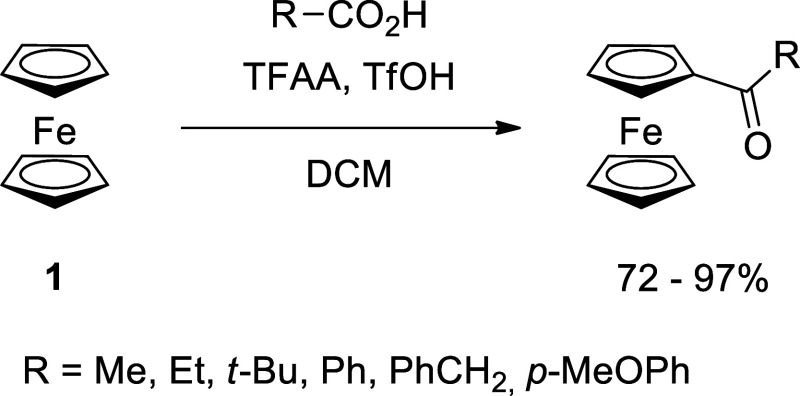
Acylation of Ferrocene with the Carboxylic Acids

There are literature reports of the Friedel–Crafts acylation reactions of ferrocene using carboxylic acids with trifluoroacetic anhydride and trifluoromethanesulfonic acid. The carboxylic acids used in these reactions are either the simple ones (Scheme 1)^66,67^ but also substrates with more complicated structure like biotin can play this role efficiently.^68,69^

In these reactions, carboxylic acid and a stoichiometric amount of TFAA form acyl trifluoroacetate in situ, which then, under protonation by trifluoromethanesulfonic acid (TfOH), generates the acylating agent. This allows one to avoid the use of AlCl_3_, which is moisture-sensitive, difficult to handle, and causes difficulties during workup (Scheme 1).

The above facts prompted us to try to apply this methodology for the use of unprotected amino acids as acylating agents. We assumed that using a 2-fold excess of TFAA, it would be possible to *in situ* protect the amino group of an amino acid with the trifluoroacetyl group and subsequently to generate acyl trifluoroacetate from such N-protected amino acid. The existing literature examples that the *N*-trifluoroacetyl group is stable against TfOH^70−72^ assured us about the chosen approach.

**Scheme 2:**
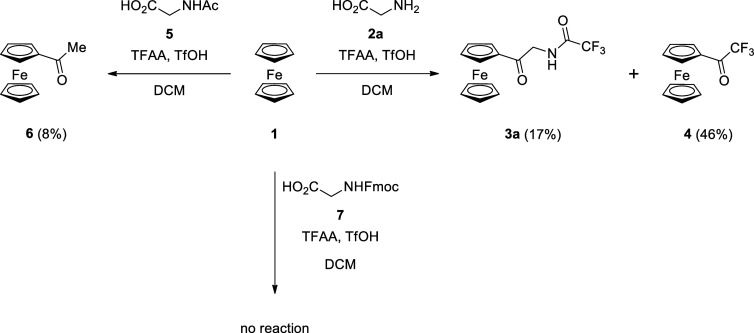
Attempts of the Acylation of Ferrocene with Glycine (**2a**) and Its Derivatives (**5** and **7**)

For our studies, we chose glycine **2a** as the simplest amino acid as a model compound. Since glycine does not dissolve in dichloromethane, we mixed 1 mmol of glycine with 2 mmol of trifluoroacetic anhydride and stirred this mixture for 15 min in anhydrous conditions to protect the amino group and convert the carboxyl group into acyl trifluoroacetate. Then, we were adding anhydrous dichloromethane, followed by ferrocene **1** (1 mmol) and TfOH (1 mmol), and stirred the reaction mixture for 1 h at ambient temperature. The workup of the reaction afforded the corresponding *N*-trifluoroacetylaminoketone **3a**, albeit with low yield, a significant amount of trifluoroacetylferrocene **4** as the main product, and also unreacted ferrocene (Scheme 2). The assumption that the *N*-trifluoroacetyl protecting group is too weak for the reaction conditions prompted us to try this reaction with N-protected glycine derivatives, namely, *N*-acetylglycine **5** and Fmoc-glycine **7**. The rationale behind it was that **5** is known of its stability in various reaction conditions,^73^ and the Fmoc group present in compound **7** is stable toward acid conditions.^74^ We conducted both reactions with only one equivalent of TFAA and found that in the first case, we obtained only acetylferrocene **6**, and in the second, no formation of the product was observed (Scheme 2).

We attempted to optimize the reaction conditions of the reaction of unprotected glycine by extending the reaction time and increasing the amount of TFAA. We also tried to extend the time of generation of acyl trifluoroacetate to ensure that most of TFAA was consumed and therefore decrease the amount of byproduct **4**. Both attempts yielded moderate success. We slightly improved the yield but still observed a significant amount of byproduct **4** as well as the unreacted ferrocene. The results are summarized in Table 1.

**Table 1:** Optimization of the Acylation of Ferrocene with Unprotected Glycine

entry	TFAA eq	acyl trifluoroacetate generation time min	reaction time h	3a	4	% of reacting Fc-H
1	2	15	1	17%	46%	97%
2	2	30	1	21%	45%	94%
3	2	30	4	25%	45%	96%
4	3	30	1	26%	44%	94%

**Scheme 3:**
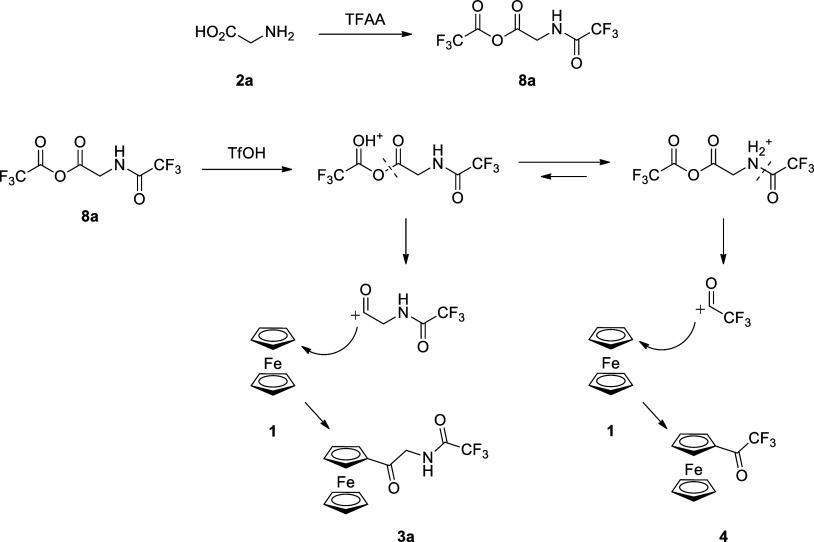
Mechanism of Formation of the Products **3a** and **4**

The possible explanation is due to the proximity of the *N*-trifluoroacetyl group and the acyl trifluoroacetate group, and the proton from TfOH can be transferred from the latter to the first and lead to the formation of trifluoroacetyl carbocation, which is responsible for the formation of **4** (Scheme 3).

The formation of acetylferrocene has been also observed in the sole example of acylation of ferrocene with *N*-acetylamino acid (6-acetamidohexanoic acid) in similar conditions, but only as a small amount of byproduct.^69^

**Scheme 4:**
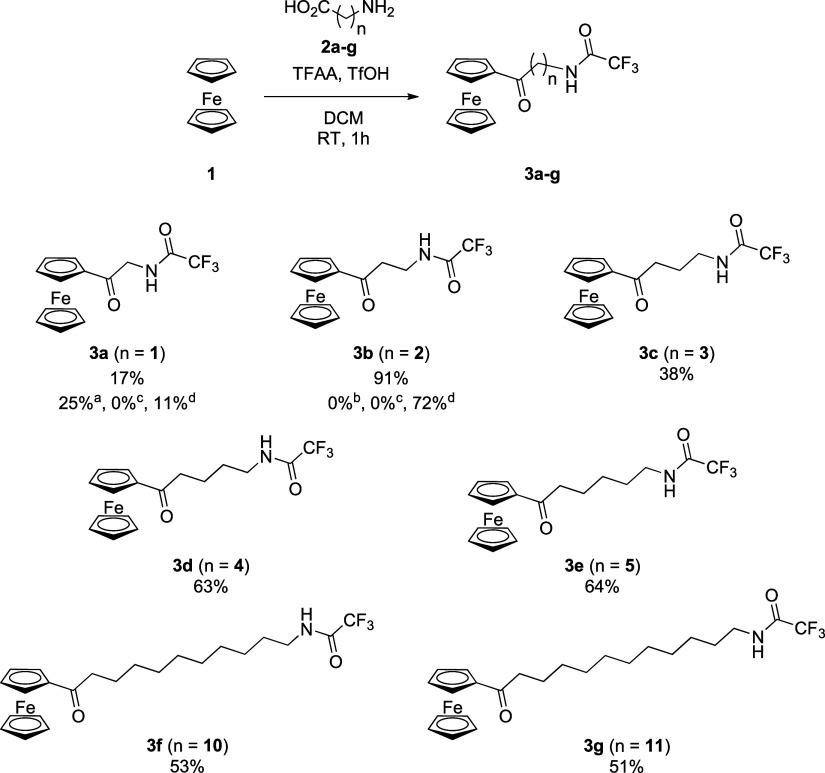
Acylation of Ferrocene with Amino Acids with Different Chain Lengths reaction time 4h. reaction without TFAA. reaction with 1 eq. H_3_PO_4_ instead of TfOH. reaction with 1 eq. BF_3_xEt_2_O instead of TfOH.

Next, we studied how the elongation of the hydrocarbon chain would affect the course of the reaction. We chose β-alanine **2b** and reacted it under basic conditions with ferrocene. After a 1 h reaction, we obtained the corresponding *N*-trifluoroacetyl amidoketone **3b** in excellent 91% yield, virtually without any byproducts, so the reaction workup required only extraction and crystallization from hexane to obtain the pure product. This showed that one more methylene group between the *N*-trifluoroacetyl group and the acyl trifluoroacetate group is sufficient to isolate them from each other enough to prevent the proton transfer and equilibrium which lead to the mixture of products. Therefore, with such an efficient reaction system in hand, we decided to extend the scope of our reaction to amino acids with a longer chain (Scheme 4).

The reactions of ferrocene with a series of amino acids, from 4-aminobutyric **2c** acid to 6-aminohexanoic acid **2e**, and two long-chain ones, 11-aminoundecanoic acid **2f** and 12-aminododecanoic acid **2g**, yielded the corresponding *N*-trifluoroacetamidoketones in good yields. In the case of products **3d** and **3e**, reactions proceed almost without byproducts and require only simple crystallization like in the case of **3b**. The long-chain products require purification by flash chromatography due to the formation of polar byproducts during the course of the reactions. We also attempted to replace the triflic acid with other acids but with moderate success. We conducted the synthesis of products **3a** (with the lowest yield) and **3b** (with the highest yield) using the orthophosphoric acid and the boron trifluoride diethyl etherate. In the reactions with orthophosphoric acid, we did not observe the formation of the product. In the ones where the boron trifluoride diethyl etherate was used, the yield was lower compared to the triflic acid (Scheme 4).

This is the first example of using unblocked amino acids of different hydrocarbon chain lengths and the positions of amino and carboxyl groups as acylating agents in the Friedel–Crafts reaction of ferrocene. Earlier literature reports include one example of using *N*-acylated 6-aminohexanoic acid in this role^69^ and the use of β-lactam and its N-protected derivatives as acylating agents, which undergo ring opening under the influence of a strong acid, with this method being limited to the synthesis of β-aminoketones.^75^

### 2.2 Study on the Reaction Mechanism

**Scheme 5:**
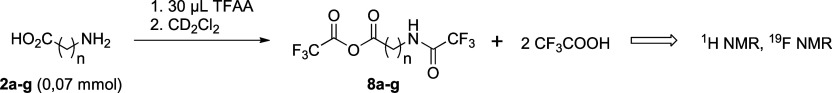
NMR Experiment to Investigate the Formation of *N*-Trifluoroacetylaminoacyl Trifluoroacetates (**8a**–**g**)

To further investigate the formation of *N*-trifluoroacetylaminoacyl trifluoroacetates from amino acids under the reaction conditions, we conducted the following experiment. We charged the NMR test tube with appropriate amino acid, apply TFAA, and left the mixture for 15 min to generate mixed anhydride under conditions similar to the reaction conditions. Then, we dissolved the reaction mixture in deuterated dichloromethane (CD_2_Cl_2_) and recorded the ^1^H and ^19^F NMR spectra (for details, see the Experimental Section). We repeat this procedure for all amino acids in series **2a**–**g** (Scheme 5).

**Figure 1:**
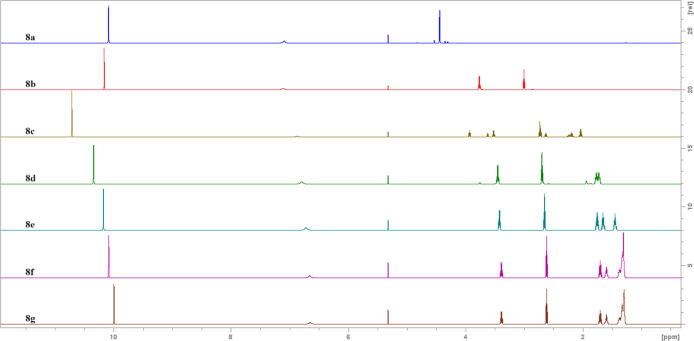
^1^H NMR spectra of acyl trifluoroacetates (**8a**–**g**) in CD_2_Cl_2_.

We obtain the series of ^1^H NMR spectra presented below (Figure 1).

**Figure 2:**
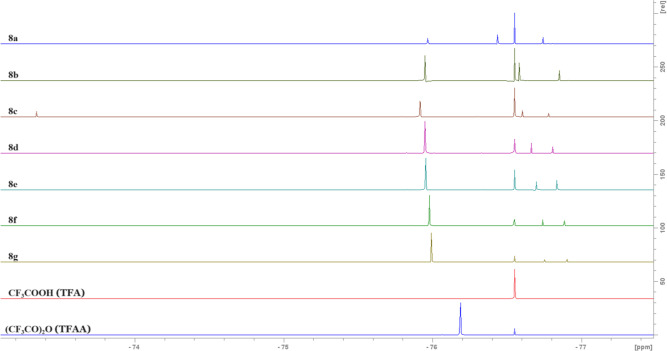
^19^F NMR spectra of acyl trifluoroacetates (**8a**–**g**) (TFA and TFAA for comparison) in CD_2_Cl_2_.

On each of them, we can observe the signal from the acidic proton from trifluoroacetic acid (TFA) around 10 ppm and the signal from the *N*-trifluoroacetylamide proton in the area between 6 and 7 ppm. On all spectra except **8c**, we observed the integration ratio 2:1 between signals of TFA and amide protons, which is consistent with the stoichiometry presented in Scheme 5 and supports the mechanism involving the formation of *N*-trifluoroacetylaminoacyl trifluoroacetates. In the case of **8c**, the integration ratio between the above-mentioned signals is 5:1, and two sets of signals are present in the alkyl region of the spectra, suggesting the formation of a mixture of products. The ^19^F NMR spectra support this hypothesis. Again on all of them, except **8c**, we can observe four signals: one from TFA, second from TFAA, and the third and fourth from *N*-trifluoroacetylamide and acyl trifluoroacetate groups, respectively (Figure 2). On spectrum **8c**, an additional fifth signal is present, at −73 ppm, which can originate from the second product, apart from the expected mixed anhydride.

**Scheme 6:**
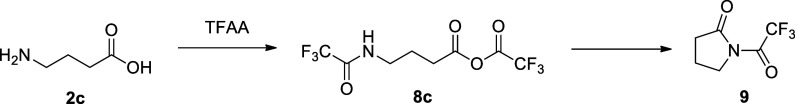
Possible Pathway of Formation of Lactam **9** from 4-Aminobutyric Acid **2c**

This second product could be 5-member *N*-trifluoroacetylated lactam **9**. This lactam may form from part of in situ generated mixed anhydride **8c** (Scheme 6).

Such a compound will contribute to ^19^F NMR spectra with a single signal from the only CF_3_ group, which is consistent with the experiment. Lactam **9** also does not undergo acid-driven ring opening, so it would be inactive in reaction conditions.^75^ This explains the lower yield of product **3c** (Scheme 4).

### 2.3 Deprotection–Reprotection Studies

**Scheme 7:**
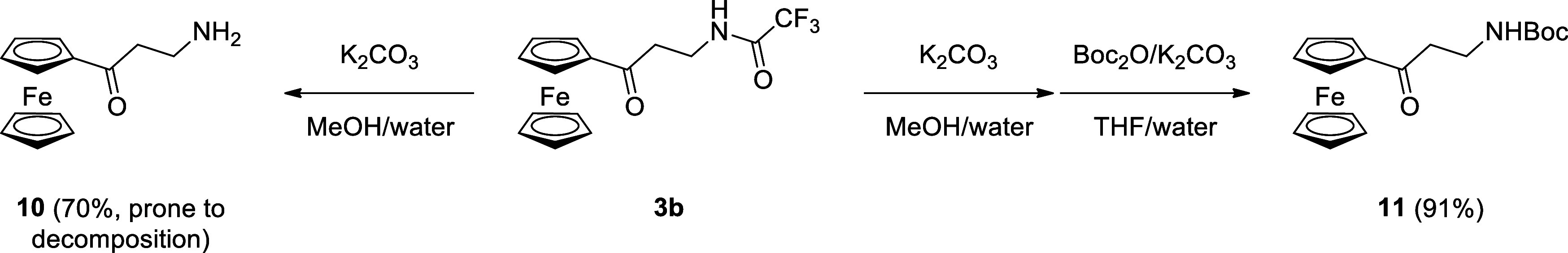
Deprotection and Deprotection/Protection of **3b**

The *N-*trifluoroacetyl group is labile under mild basic conditions,^76,77^ allowing its easy deprotection. We decided to try such deprotection on compound **3b** to obtain its aminoketone derivative **10** (Scheme 7). To achieve this, we treated compound **3b** with an excess of potassium carbonate in a mixture of methanol and water.

We found by comparative TLC that in the course of the reaction, the product is more polar than the substrate is formed, which suggested the formation of β-aminopropionyl ferrocene **10**. However, it turned out that this compound undergoes slow (few days) decomposition, when exposed to air and room temperature. This decomposition undergoes probably due to the simultaneous presence of the carbonyl group and the free amino group in the molecule. To solve this problem, we modified the deprotection procedure to the deprotection and subsequent protection of the free aminoketone with the Boc group. Since such protection can be done in aqueous conditions,^78^ we were able to achieve it in a one-pot reaction. The only issue is to replace methanol with tetrahydrofuran between the deprotection and subsequent Boc protection to make the latter possible. The reaction proceeds with excellent yield, giving the bench-stable compound **11**. Such Boc derivative can be easily deprotected^79,80^ before use in synthesis, which makes it a very useful building block.

### 2.4 Crystallographic Studies

**Figure 3:**
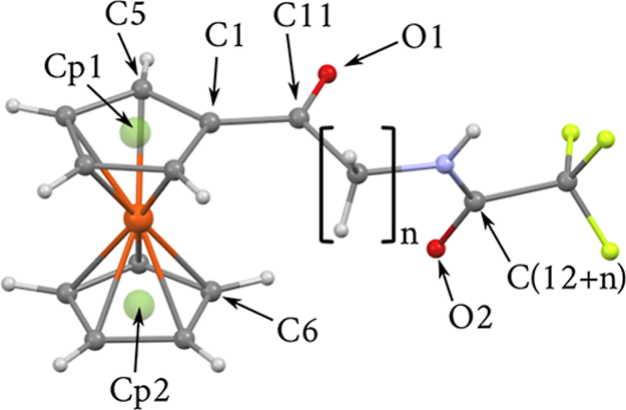
Numbering scheme common for all described structures.

Crystal structures for compounds **3a**–**3g** and **11** were determined by means of single-crystal X-ray diffraction. Detailed information about data processing, structure solution, and refinement is shown in Table S1 in the Supporting Information. Atom and centroid labeling used for structure description is shown in Figure 3. In the case of **3a** and **3e**, there were, respectively, three and two molecules in the crystallographic asymmetric unit. They were denoted as molecules I–III or I–II depending on the number of the iron atoms.

Ferrocene conformation in almost all structures was typical, close to eclipsed, with relative rotation of cyclopentadienyl (Cp) rings in the range −7.18–6.74° (Table S4). Only for **3f**, there was staggered conformation (relative rotation of −23.13°). Distance between Cp rings was in the range of 3.26–3.32 Å.

The C1–C11 bond was close to 1.46–1.48 Å, which suggests a proper single bond (Table S2.). As expected, the carbonyl group C11–O1 bound to cyclopentadienyl was almost in plane with the ring. The biggest out-of-plane bend was found in the structure of **3a** (*n* = 1) and equaled 11.7(5)°. For the majority of structures, the beginning of the alkyl chain was almost in the cyclopentadienyl plane, with rotation around the first single C–C bond in the range of −160–178°. Only for **3f** alkyl chain was bent at the Cp end (dihedral angle −75(2)°). Different behavior was visible at the amide end of the alkyl chain. It was bent for most structures and almost straight (rotated by less than 10°) for the structures with longest alkyl chain −**3f** and **3g** (*n* = 10 and 11, Figure S3 in the Supporting Information).

**Figure 4:**
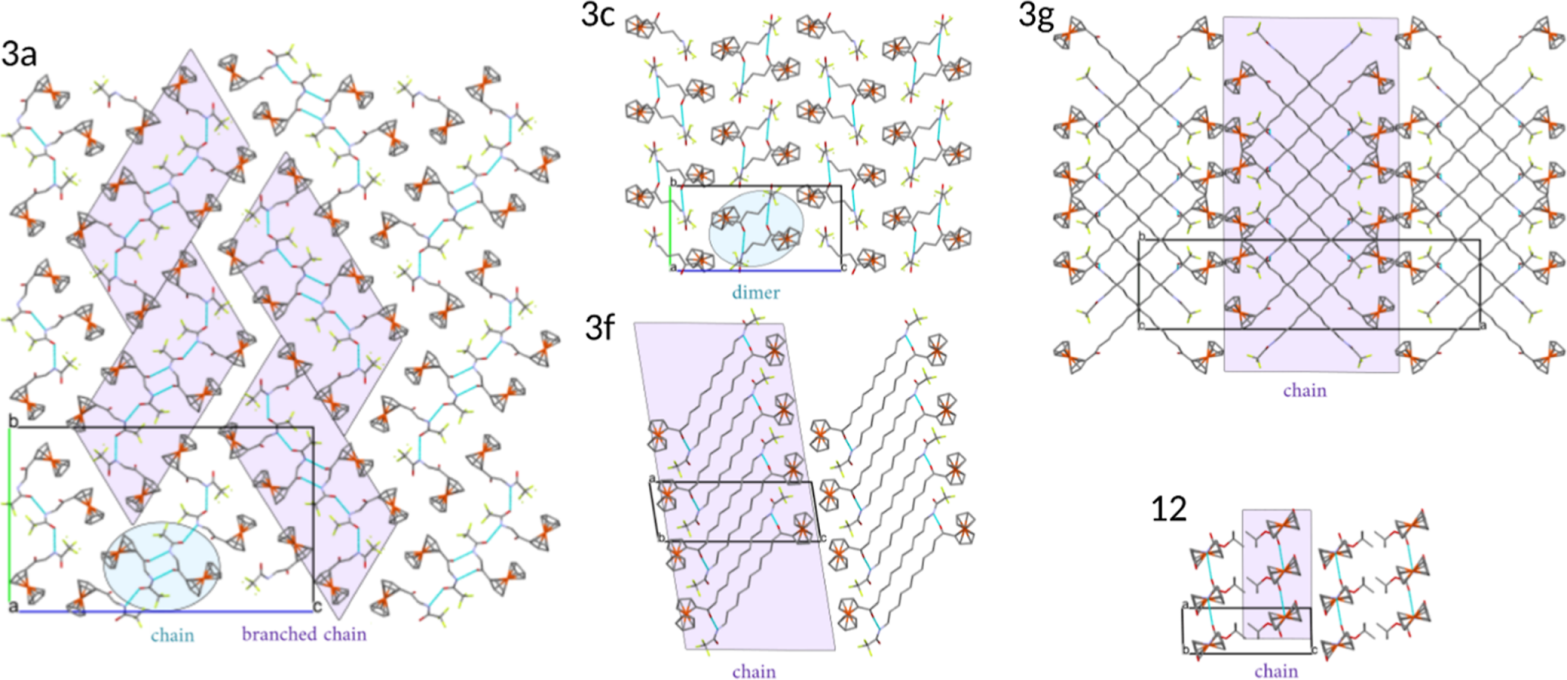
Crystal packing in described structures with supramolecular motifs related to H-bond presence highlighted: **3a** in the [100] direction, **3c** in the [100] direction, **3g** in the [010] direction, **3f** in the [010] direction, and **11** in the [010] direction.

Molecules in all structures were interacting by hydrogen bonds between the amide group and carbonyl group bonded to Cp (for **3a**–**g**) or from other amide groups (for **3a** and **11**). The geometry of hydrogen bonds is shown in Table S1, and motifs are shown in Figure S2. In **3a**, hydrogen bond interactions built a branched chain (Figure 4). The chain core (network C(5) in Etter notation^81^) consisted of molecules I, where the hydrogen atoms bonded to the amide group interacted with the oxygen atoms from the carbonyl group bonded to Cp (O1). Each second oxygen atom (O2–from amide moiety) of molecule I was an acceptor to the hydrogen bond from the amide group of molecule II. Similarly, molecule III was a donor of the hydrogen bond to the oxygen atom of the amide group in the molecule (Figure S2).

**3b**–**d** had different hydrogen bond motifs, which can be described as *R*(4 + *n*). Their molecules formed dimers, acting as a donor and an acceptor (Figures 4 and S2b). The longer aliphatic chain in **3e**, **3f**, and **3g** and the Boc moiety in **11** resulted in chain formation by hydrogen bonds. Aliphatic chains were parallel in structure with even *n* value (**3f**, *n* = 10, Figure 4: **3f**) and crossed when the *n* value was odd (**3e** and **3g**, *n* = 5 and *n* = 11, Figure 4: **3g**), in both cases, resulting in the C(4 + *n*) motif. The Boc derivative in hydrogen bond formation contributed only amide moiety (Figure S2e), creating the C(4) network.

There were no visible differences between the geometry of hydrogen bonds as a function of aliphatic chain length. All N···O distances in structures of **3a**–**g** were in the range 2.83–2.93 Å, and N–H···O angles were 149–169°. The structure of **11**, because of steric bulk due to the Boc moiety, was an outlier with N···O distance 3.43 Å.

Hydrogen bond motifs have an impact on crystal packing. In the structure of **3a**, there were columns of branched chains in the [100] direction, creating a herringbone pattern on the (100) plane (Figure 4: **3a**). There were no distinguishable layers. In structures of **3b**–**d**, dimers were separated. Layers were formed in structures with longer aliphatic chains **3e**–**g** or with Boc derivative **11**. Nonparallelism in H-bond chain formation resulted in layers with crossed aliphatic chains (Figure 4: **3g**). Crystal packing tended to maximize the number of Fc···Fc interactions.

## 3 Conclusions

In this study, we obtained a series of trifluoroacetamide ketones by the acylation of ferrocene with unprotected amino acids. We used trifluoroacetic anhydride to protect and activate the amino acids simultaneously and trifluoromethanesulfonic acid (TfOH) as a proton source. We proposed a mechanism for this reaction, involving the *in situ* creation of *N*-trifluoroacetylaminoacyl trifluoroacetate, which is subsequently protonated by TfOH to generate a carbocation. Our study demonstrated the possibility of using unprotected amino acids as acylating agents, allowing these reactions to be conducted under relatively mild conditions. This approach gradually expanded the scope of Friedel–Crafts reactions with mixed anhydrides. Additionally, the obtained ferrocene trifluoroacetamide ketones could easily undergo deprotection or a deprotection–protection procedure to yield β-aminopropyl ferrocene derivatives in good yields.

## 4 Experimental Section

### 4.1 General Information

All reactions were carried out in an Ar atmosphere using typical glassware. Commercially available reagents and solvents were used as received. Column chromatography was performed on silica gel 60 (230–400 mesh) purchased from Merck. Thin-layer chromatography was performed on aluminum sheets precoated with silica purchased from Silicycle. Elemental analysis were performed on an Elementar Vario Micro Cube. Infrared (IR) spectra were recorded in KBr on a Fourier Transform InfraRed (FTIR) NEXUS (Thermo Nicolet) spectrometer. Electrospray ionization mass spectrometry (ESI-MS) spectra were recorded on a Varian 500 MS LC ion trap spectrometer. High-resolution mass spectrometry (HRMS) measurements were performed using a Synapt G2 Si mass spectrometer (Waters) equipped with an ESI source and a quadrupole-time-of-flight mass analyzer. ^1^H and ^13^C{^1^H} NMR spectra were recorded at 300 K on a Bruker UltraShield Avance III 600 MHz spectrometer (600.26 MHz for ^1^H and 150.94 MHz for ^13^C) equipped with a BBFO probe and a Bruker Ascend Neo 600 MHz spectrometer (600.14 MHz for ^1^H and 150.90 MHz for ^13^C) equipped with a Prodigy cryoprobe. ^19^F NMR spectra were recorded at 300 K on a Bruker Ascend Neo 600 MHz spectrometer (564.70 MHz for ^19^F). Chemical shifts were calibrated on the residual solvent signals: CDCl_3_ 7.26 ppm for ^1^H and 77.16 ppm for ^13^C, CD_3_OD 3.31 ppm for ^1^H and 49.00 ppm for ^13^C, and CD_2_Cl_2_ 5.32 ppm for ^1^H and 53.84 ppm for ^13^C.^82^ Melting points were measured with a Stanford Research Systems DigiMelt MPA161.

### 4.2 Synthesis of **3a–g**

Corresponding amino acid (1 mmol, 1 equiv) was stirred with TFAA (280 μL, 2 mmol, 2 equiv) in a 10 mL round-bottom flask equipped with a magnetic stirring bar until complete dissolution. Dry DCM (5 mL) was added, followed by ferrocene (186 mg, 1 mmol, 1 equiv) and triflic acid (90 μL, 1 mmol, 1 equiv). Reaction progress was monitored by TLC. After 1 h, the reaction was quenched by pouring into ice–water (50 mL) and then placing in a separatory funnel. The organic layer was washed with water (3 × 25 mL) and brine (1 × 25 mL) and dried over anhydrous MgSO_4_. The solvent was removed under reduced pressure, and the crude product was purified by crystallization or column chromatography on silica gel.

#### 4.2.1 3a

Purified by column chromatography using 1:1 DCM/hexane as an eluent. The product was crystallized from DCM/hexane. Orange–red crystals (71 mg, 21%), mp 98.6–100.3 °C. ^1^H NMR (600 MHz, CDCl_3_): δ 7.43 (s, 1H), 4.87 (t, *J =* 1.7 Hz, 2H), 4.65 (t, *J =* 1.7 Hz, 2H), 4.52 (d, *J =* 4.3 Hz, 2H), 4.25 (s, 5H). ^13^C{^1^H} NMR (151 MHz, CDCl_3_): δ 196.4, 76.9, 75.2, 73.5, 70.5, 69.2, 46.5. ^19^F NMR (565 MHz, CDCl_3_): δ −75.64. IR (KBr, cm^–1^): 3292, 3101, 2933, 1734, 1711, 1682, 1558, 1464, 1438, 1323, 1252, 1211, 1188, 1144, 1106, 1047, 818. MS (ESI) *m*/*z*: [M + Na]^+^ calcd for C_14_H_12_F_3_FeNO_2_Na^+^ 362.0; found, 362.1. Anal. Calcd for C_14_H_12_F_3_FeNO_2_: C, 49.59; H, 3.57; N, 4.13. Found: C, 49.55; H, 3.73; N, 4.38.

#### 4.2.2 3b

Purified by crystallization from DCM/hexane. Orange-red crystals (321 mg, 91%), mp 127.2–128.1 °C. ^1^H NMR (600 MHz, CDCl_3_): δ 7.25 (s, 1H), 4.79 (t, *J =* 1.9 Hz, 2H), 4.57 (t, *J =* 1.9 Hz, 2H), 4.19 (s, 5H), 3.74 (q, *J =* 5.8 Hz, 2H), 3.03 (t, *J =* 5.6 Hz, 2H). ^13^C{^1^H} NMR (151 MHz, CDCl_3_): δ 203.5, 78.0, 73.1, 70.1, 69.4, 38.1, 35.0. ^19^F NMR (565 MHz, CDCl_3_): δ −76.05. IR (KBr, cm-1): 3239, 3071, 2966, 2910, 1714, 1646, 1552, 1458, 1279, 1244, 1200, 1176, 1153, 1079, 1003, 824. MS (ESI) *m*/*z*: [M + H]^+^ calcd for C_15_H_15_F_3_FeNO_2_^+^ 354.0; found, 354.1. Anal. Calcd for C_15_H_14_F_3_FeNO_2_: C, 51.02; H, 4.00; N, 3.97. Found: C, 51.23; H, 4.02; N, 3.91.

#### 4.2.3 3c

Purified by column chromatography using DCM/MeOH 200:1 as an eluent. The product was crystallized from DCM/hexane. Orange-red crystals (141 mg, 38%), mp 86.5–87.4 °C. ^1^H NMR (600 MHz, CDCl_3_): δ 7.50 (s, 1H), 4.80 (t, *J =* 1.9 Hz, 2H), 4.55 (t, *J =* 1.9 Hz, 2H), 4.19 (s, 5H), 3.45 (q, *J =* 6.1 Hz, 2H), 2.89 (t, *J =* 6.4 Hz, 2H), 2.02 (quint, *J =* 6.4 Hz, 2H). ^13^C{^1^H} NMR (151 MHz, CDCl_3_): δ 205.1, 78.3, 72.9, 70.0, 69.6, 40.5, 37.6, 22.6. ^19^F NMR (565 MHz, CDCl_3_): δ −75.92. IR (KBr, cm^–1^): 3260, 3095, 2951, 2892, 1720, 1649, 1561, 1461, 1211, 1179, 1150, 820. MS (ESI) *m*/*z*: [M + H]^+^ calcd for C_16_H_17_F_3_FeNO_2_^+^ 368.1; found, 368.1. Anal. Calcd for C_16_H_16_F_3_FeNO_2_: C, 52.34; H, 4.39; N, 3.82. Found: C, 52.27; H, 4.53; N, 3.60.

#### 4.2.4 3d

Purified by crystallization from DCM/hexane. Orange-red crystals (240 mg, 63%), mp 99.8–101.6 °C. ^1^H NMR (600 MHz, CDCl_3_): δ 6.90 (br s, 1H), 4.79 (t, *J =* 1.9 Hz, 2H), 4.52 (t, *J =* 1.9 Hz, 2H), 4.20 (s, 5H), 3.40 (q, *J =* 6.4 Hz, 2H), 2.79 (t, *J =* 6.7 Hz, 2H), 1.77 (m, 2H), 1.68 (m, 2H). ^13^C{^1^H} NMR (151 MHz, CDCl_3_): δ 204.4, 78.7, 72.4, 69.85, 69.32, 39.5, 38.5, 28.4, 20.6. ^19^F NMR (565 MHz, CDCl_3_): δ −75.87. IR (KBr, cm^–1^): 3280, 3083, 2951, 1720, 1661, 1558, 1455, 1358, 1255, 1208, 1182, 1156, 1103, 818. MS (ESI) *m*/*z*: [M + H]^+^ calcd for C_17_H_19_F_3_FeNO_2_^+^ 382.1; found, 382.2. Anal. Calcd for C_17_H_18_F_3_FeNO_2_: C, 53.57; H, 4.76; N, 3.67. Found: C, 53.58; H, 4.80; N, 3.54.

#### 4.2.5 3e

Purified by crystallization from DCM/hexane. Orange-red crystals (254 mg, 64%), mp 71.8–73.3 °C. ^1^H NMR (600 MHz, CDCl_3_): δ 6.65 (br s, 1H), 4.78 (t, *J =* 1.9 Hz, 2H), 4.51 (t, *J =* 1.8 Hz, 2H), 4.19 (s, 5H), 3.43 (q, *J =* 6.7 Hz, 2H), 2.74 (t, *J =* 7.1 Hz, 2H), 1.74 (quint, *J =* 7.5 Hz, 2H), 1.65 (quint, *J =* 7.3 Hz, 2H), 1.43 (m, 2H). ^13^C{^1^H} NMR (151 MHz, CDCl_3_): δ 204.5, 79.1, 72.4, 69.92, 69.43, 39.6, 39.3, 28.7, 26.4, 23.4. ^19^F NMR (565 MHz, CDCl_3_): δ −75.85. IR (KBr, cm^–1^): 3289, 3080, 2945, 2866, 1711, 1658, 1555, 1458, 1255, 1197, 1182, 1150, 820. MS (ESI) *m*/*z*: [M + H]^+^ calcd for C_18_H_21_F_3_FeNO_2_^+^ 396.1; found, 396.2. Anal. Calcd for C_18_H_20_F_3_FeNO_2_: C, 54.71; H, 5.10; N, 3.54. Found: C, 54.57; H, 5.15; N, 3.68.

#### 4.2.6 3f

Purified by column chromatography using a DCM/MeOH 400:1 as an eluent. The product was crystallized from DCM/hexane. Orange powder (246 mg, 53%), mp 58.1–59.5 °C. ^1^H NMR (600 MHz, CDCl_3_): δ 6.33 (s, 1H), 4.78 (t, *J =* 1.9 Hz, 2H), 4.49 (t, *J =* 1.9 Hz, 2H), 4.19 (s, 5H), 3.36 (q, *J =* 6.8 Hz, 2H), 2.69 (t, *J =* 7.4 Hz, 2H), 1.70 (quint, *J =* 7.4 Hz, 2H), 1.58 (quint, *J =* 7.2 Hz, 2H), 1.35 (m, 12H). ^13^C{^1^H} NMR (151 MHz, CDCl_3_):δ 204.9, 79.3, 72.3, 69.88, 69.47, 40.1, 39.9, 29.58, 29.53, 29.40, 29.16, 29.04, 26.7, 24.7. ^19^F NMR (565 MHz, CDCl_3_): δ −75.91. IR (KBr, cm^–1^): 3301, 3083, 2939, 2916, 2851, 1705, 1646, 1552, 1458, 1376, 1200, 1164, 1108, 824. MS (ESI) *m*/*z*: [M + H]^+^ calcd for C_23_H_31_F_3_FeNO_2_^+^ 466.2; found, 466.4. Anal. Calcd for C_23_H_30_F_3_FeNO_2_: C, 59.37; H, 6.50; N, 3.01. Found: C, 59.52; H, 6.55; N, 2.91.

#### 4.2.7 3g

Purified by column chromatography using DCM/MeOH 400:1 as an eluent. The product was crystallized from DCM/hexane. Orange powder (246 mg, 51%), mp 79.4–80.9 °C. ^1^H NMR (600 MHz, CDCl_3_): δ 6.31 (s, 1H), 4.78 (t, *J =* 1.9 Hz, 2H), 4.49 (t, *J =* 1.9 Hz, 2H), 4.19 (s, 5H), 3.36 (q, *J =* 6.8 Hz, 2H), 2.69 (t, *J =* 7.4 Hz, 2H), 1.70 (quint, *J =* 7.4 Hz, 2H), 1.58 (quint, *J =* 7.4 Hz, 2H), 1.33 (m, 14H). ^13^C{^1^H} NMR (151 MHz, CDCl_3_):δ 204.9, 79.4, 72.2, 69.88, 69.49, 40.2, 39.9, 29.64, 29.57, 29.52, 29.46, 29.19, 29.08, 26.8, 24.8. ^19^F NMR (565 MHz, CDCl_3_): δ −75.93. IR (KBr, cm^–1^): 3321, 3083, 2916, 2851, 1714, 1655, 1549, 1473, 1455, 1385, 1270, 1202, 1182, 1153, 820. MS (ESI) *m*/*z*: [M + H]^+^ calcd for C_24_H_33_F_3_FeNO_2_^+^ 480.2; found, 480.4. Anal. Calcd for C_24_H_32_F_3_FeNO_2_: C, 60.13; H, 6.73; N, 2.92. Found: C, 60.20; H, 6.81; N, 2.84.

### 4.3 Synthesis of **10**

Compound **3b** (353 mg, 1 mmol) was suspended in MeOH/water 1:1 (10 mL) in a 25 mL round-bottom flask equipped with a magnetic stirring bar, and potassium carbonate (1.38 g, 10 mmol) was added to the reaction mixture. Reaction was stirred overnight when TLC indicated completion of deprotection. One M NaOH (3 mL) was added to the reaction mixture, and the product was extracted with an EtOAc/MeOH 20:1 solution. Combined organic extracts were washed with brine and dried over anhydrous Na_2_SO_4_. The solvent was removed under reduced pressure, and the crude product was purified by column chromatography using DCM/MeOH 20:1 as an eluent. The compound slowly decomposes and should be stored in the dark under argon at −20 °C. Reddish oil (180 mg, 70%). ^1^H NMR (600 MHz, CD_3_OD): δ 4.85 (t, *J* = 1.7 Hz, 2H), 4.62 (t, *J* = 1.7 Hz, 2H), 4.26 (s, 5H), 3.06 (m, 4H). ^13^C{^1^H} NMR (151 MHz, CD_3_OD): δ 205.7, 79.7, 74.0, 71.1, 70.5, 41.2, 37.5. IR (KBr, cm^–1^): 3500, 3442, 3348, 3097, 2961, 2924, 1664, 1479, 1454, 1379, 1252, 1103, 821. HRMS (ESI-TOF) *m*/*z*: [M + H]^+^ calcd for C_13_H_16_FeNO^+^ 258.0576; found, 258.0578.

### 4.4 Synthesis of **11**

Compound **3b** (353 mg, 1 mmol) was suspended in MeOH/water 1:1 (10 mL) in a 25 mL round-bottom flask equipped with a magnetic stirring bar, and potassium carbonate (1.38 g, 10 mmol) was added to the reaction mixture. The reaction was stirred overnight when TLC indicated completion of deprotection. MeOH was removed by evaporation and replaced with THF (5 mL), and Boc_2_O (436 mg, 2 mmol) was added to the reaction mixture. After 3 h, TLC indicated completion of the reaction. The reaction mixture was concentrated to remove THF and taken between water and DCM. The organic layer was washed with 1 M HCl and brine and dried over anhydrous MgSO_4_. The solvent was removed under reduced pressure, and the crude product was purified by column chromatography using DCM/MeOH 200:1 as an eluent. The product **11** was crystallized from DCM/hexane. Orange powder (324 mg, 91%), mp 93.1–94.6 °C. ^1^H NMR (600 MHz, CDCl_3_): δ 5.17 (br s, 1H), 4.78 (t, *J* = 1.7 Hz, 2H), 4.52 (t, *J =* 1.8 Hz, 2H), 4.20 (s, 5H), 3.50 (q, *J* = 5.7 Hz, 2H), 2.95 (t, *J* = 5.3 Hz, 2H), 1.43 (s, 9H). ^13^C{^1^H} NMR (151 MHz, CDCl_3_): δ 203.6, 156.1, 79.4, 78.9, 72.6, 70.0, 69.4, 39.6, 35.6, 28.6. IR (KBr, cm^–1^): 3424, 3377, 3359, 3130, 2972, 2936, 1702, 1655, 1499, 1452, 1367, 1288, 1247, 1164, 1085, 973, 824. MS (ESI) *m*/*z*: M^+^ calcd for C_18_H_23_FeNO_3_357.1; found, 357.3. Anal. Calcd for C_18_H_23_FeNO_3_: C, 60.52; H, 6.49; N, 3.92. Found: C, 60.64; H, 6.38; N, 3.87.

### 4.5 ^1^H and ^19^F NMR Experiments (**8a–g** and **9**)

The NMR test tube was charged with an appropriate amino acid (0.07 mmol, 1 equiv), TFAA (30 μL, 0.22 mmol, 3.1 equiv) was added, and the tube was closed with a stopper and left for 15 min to generate *N*-trifluoroacetylaminoacyl trifluoroacetate. Then, CD_2_Cl_2_ (0.5 mL) was added to the tube, and ^1^H, ^13^C{^1^H}, and ^19^F NMR spectra were recorded. All compounds were generated in situ and have not been separated from the reaction mixture.

#### 4.5.1 8a

^1^H NMR (600 MHz, CD_2_Cl_2_): δ 7.09 (s, 1H), 4.44 (d, *J* = 5.8 Hz, 2H). ^13^C{^1^H} NMR (151 MHz, CD_2_Cl_2_): δ 161.8, 160.9, 160.6, 160.3, 160.0, 152.5, 152.2, 151.9, 151.6, 117.9, 117.1, 116.0, 115.18, 115.14, 114.2, 113.2, 112.3, 42.6. ^19^F NMR (565 MHz, CD_2_Cl_2_): δ −76.44, −76.74. HRMS (ESI-TOF) *m*/*z*: [M – H]^−^ calcd for C_6_H_2_F_6_NO_4_^–^ 265.9894; found, 265.9891.

#### 4.5.2 **8b**

^1^H NMR (600 MHz, CD_2_Cl_2_): δ 7.11 (s, 1H), 3.76 (q, *J* = 6.1 Hz, 2H), 3.00 (t, *J* = 6.0 Hz, 2H). ^13^C{^1^H} NMR (151 MHz, CD_2_Cl_2_):δ 165.4, 159.8, 159.5, 159.2, 159.0.153.0, 152.7, 152.4, 152.1, 117.9, 117.1, 116.0, 115.25, 115.20, 114.1, 113.3, 112,3, 35.5, 34.6. ^19^F NMR (565 MHz, CD_2_Cl_2_): δ −76.58, −76.85. HRMS (ESI-TOF) *m*/*z*: [M – H]^−^ calcd for C_7_H_4_F_6_NO_4_^–^ 279.0204; found, 279.0204.

#### 4.5.3 **8c** and **9** (mixture) **8c**

^1^H NMR (600 MHz, CD_2_Cl_2_): δ 6.87 (s, 1H),3.52 (q, *J* = 6.7 Hz, 2H), 2.73 (m, 2H), 2.04 (quint, *J* = 7.0 Hz, 2H). ^13^C{^1^H} NMR (151 MHz, CD_2_Cl_2_): δ 166.0, 159.7, 159.4, 159.2, 153.3, 153.0, 152.7, 152.4, 117.8, 117.2, 116.0, 115.3, 115.2, 114.1, 113.3, 112.2, 32.6, 30.8, 23.4, 18.2. ^19^F NMR (565 MHz, CD_2_Cl_2_): δ −76.61, −76.78. HRMS (ESI-TOF) *m*/*z*: [M – H]^−^ calcd for C_8_H_6_F_6_NO_4_^–^ 294.0207; found, 294.0203. **9**(83)^1^H NMR (600 MHz, CD_2_Cl_2_): δ 3.93 (t, *J* = 7.2 Hz, 2H), 2.72 (m, 2H), 2.19 (m, 2H). ^13^C{^1^H} NMR (151 MHz, CD_2_Cl_2_): δ 160.2, 159.9, 159.6, 159.3, 116.7, 114.8, 47.3, 45.3, 39.7. ^19^F NMR (565 MHz, CD_2_Cl_2_): δ −73.34. HRMS (ESI-TOF) *m*/*z*: [M + H]^+^ calcd for C_6_H_7_F_3_NO_2_^+^ 182.0423; found, 182.0425.

#### 4.5.4 8d

^1^H NMR (600 MHz, CD_2_Cl_2_): δ 6.79 (s, 1H), 3.45 (q, *J* = 6.6 Hz, 2H), 2.70 (t, *J* = 6.9 Hz, 2H), 1.74 (m, 4H). ^13^C{^1^H} NMR (151 MHz, CD_2_Cl_2_): δ 166.1, 159.4, 159.2, 158.9, 158.7, 153.5, 153.2, 152.9, 152.6, 117.9, 117.2, 116.0, 115.3, 115.2, 114.1, 113.3, 112.2, 40.4, 34.7, 28.1, 21.2. ^19^F NMR (565 MHz, CD_2_Cl_2_): δ −76.67, −76.81. HRMS (ESI-TOF) *m*/z: [M – H]– calcd for C_9_H_8_F_6_NO_4_^–^ 308.0363; found, 308.0357.

#### 4.5.5 8e

^1^H NMR (600 MHz, CD_2_Cl_2_): δ 6.72 (s, 1H), 3.42 (q, *J* = 6.8 Hz, 2H), 2.65 (t, *J* = 7.2 Hz, 2H), 1.75 (m, 2H), 1.65 (m, 2H), 1.45 (m, 2H). ^13^C{^1^H} NMR (151 MHz, CD_2_Cl_2_): δ 166.2, 159.8, 159.5, 159.2, 158.9, 153.6, 153.3, 153.0, 152.7, 117.9, 117.3, 116.0, 115.4, 115.3, 114.1, 113.4, 111.5, 40.7, 35.2, 28.7, 26.1, 23.7. ^19^F NMR (565 MHz, CD_2_Cl_2_): δ −76.70, −76.84. HRMS (ESI-TOF) *m*/*z*: [M – H]^−^ calcd for C_10_H_10_F_6_NO_4_^–^ 322.0520; found, 322.0523.

#### 4.5.6 8f

^1^H NMR (600 MHz, CD_2_Cl_2_): δ 6.66 (s, 1H), 3.39 (q, *J* = 6.8 Hz, 2H), 2.62 (t, *J* = 7.4 Hz, 2H), 1.70 (quint, *J* = 7.4 Hz, 2H), 1.60 (quint, *J* = 7.2 Hz, 2H), 1.35 (m, 12H). ^13^C{^1^H} NMR (151 MHz, CD_2_Cl_2_): δ 166.4, 159.2, 158.9, 158.7, 158.4, 153.7, 153.4, 153.1, 152.8, 119.2, 117.4, 116.0, 115.4, 115.3, 114.1, 113.4, 111.6, 41.1, 35.5, 29.8, 29.7, 29.52, 29.50, 29.12, 29.08, 27.1, 24.3. ^19^F NMR (565 MHz, CD_2_Cl_2_): δ −76.74, −76.89. HRMS (ESI-TOF) *m*/*z*: [M – H]^−^ calcd for C_15_H_20_F_6_NO_4_^–^ 392.1302; found, 392.1302.

#### 4.5.7 8g

^1^H NMR (600 MHz, CD_2_Cl_2_): δ 6.65 (s, 1H), 3.39 (q, *J* = 6.8 Hz, 2H), 2.62 (t, *J* = 7.4 Hz, 2H), 1.70 (quint, *J* = 7.4 Hz, 2H), 1.60 (quint, *J* = 7.3 Hz, 2H), 1.35 (m, 14H). ^13^C{^1^H} NMR (151 MHz, CD_2_Cl_2_): δ 166.4, 159.2, 159.0, 158.7, 158.5, 153.8, 153.5, 153.2, 152.9, 119.3, 117.4, 166.0, 115.5, 115.4, 114.2, 113.6, 113.5, 41.2, 35.5, 29.9, 29.8, 29.60, 29.57, 29.18, 29.14, 27.1, 24.4. ^19^F NMR (565 MHz, CD_2_Cl_2_): δ −76.75, −76.90. HRMS (ESI-TOF) *m*/*z*: [M – H]^−^ calcd for C_16_H_22_F_6_NO_4_^–^ 406.1459; found, 406.1452.

### 4.6 X-ray Diffraction Experiments

Crystals of **3a**–**g** and **11** were obtained by *n*-pentane vapor diffusion into a dichloromethane solution in NMR-like thin glass sample tubes. Data for **3a** were collected using a SuperNova diffractometer with a microfocus sealed source of MoKα X-ray radiation (λ = 0.71073 Å) and a CCD Eos detector. Data for the remaining data sets were collected using a SuperNova diffractometer with a microfocus sealed source of CuKα X-ray radiation (λ = 1.54184 Å) and a Hybrid Pixel Array detector. Low temperature was achieved and maintained by keeping crystals in a cold nitrogen stream using an Oxford Cryosystems liquid nitrogen device. Data reduction was performed with CrysAlisPro.^81^ Gaussian absorption correction was applied using spherical harmonics with the SCALE3 ABSPACK algorithm. Crystal fae indexing was performed for all data sets but **3c**, **3d**, and **3f**. The structure was solved with SHELXT and refined with SHELXL in Olex2.^84−86^ Detailed information about data processing, structure solution, and refinement is shown in Table 1. Crystal structures were deposited with the Cambridge Crystallographic Data Centre (**3a**—deposition number 2370728, **3b**—deposition number 2370783, **3c**—deposition number 2370787, **3d**—deposition number 2370786, **3e**—deposition number 2370729, **3f**—deposition number 2370784, **3g**—deposition number 2370785, and **11**—deposition number 2370730).

## Data Availability

The data underlying this study are available in the published article and its Supporting Information The crystallographic data are openly available in CCDC at https://www.ccdc.cam.ac.uk/structures/.

## Supporting Information Available

^1^H, ^13^C{^1^H}, ^19^F NMR, and IR spectra for compounds **3a**–**g**; ^1^H, ^13^C{^1^H} NMR, and IR spectra for compounds **10** and **11**; ^1^H, ^13^C{^1^H}, and ^19^F NMR spectra for experiments **8a**–**g** and **9**; HRMS spectra for experiments **8a**–**g**, **9**, and **10**; and X-ray diffraction data for compounds **3a**–**g** and **11** (PDF)

## References

[ref1] Allen L. A. T.; Raclea R.-C.; Natho P.; Parsons P. J. Recent advances in the synthesis of α-amino ketones. Org. Biomol. Chem. 2021, 19, 498–513. 10.1039/D0OB02098B. 33325975

[ref2] Hammouda M. M.; Elattar K. M. Recent progress in the chemistry of β-aminoketones. RSC Adv. 2022, 12, 24681–24712. 10.1039/D2RA03864A. 36128366 PMC9428906

[ref3] Blough B. E.; Landavazo A.; Partilla J. S.; Baumann M. H.; Decker A. M.; Page K. M.; Rothman R. B. Hybrid Dopamine Uptake Blocker–Serotonin Releaser Ligands: A New Twist on Transporter-Focused Therapeutics. ACS Med. Chem. Lett. 2014, 5 (6), 623–627. 10.1021/ml500113s. 24944732 PMC4060932

[ref4] Meltzer P. C.; Butler D.; Deschamps J. R.; Madras B. K. 1-(4-Methylphenyl)-2-pyrrolidin-1-yl-pentan-1-one (Pyrovalerone) Analogues: A Promising Class of Monoamine Uptake Inhibitors. J. Med. Chem. 2006, 49 (4), 1420–1432. 10.1021/jm050797a. 16480278 PMC2602954

[ref5] Myers M. C.; Wang J.; Iera J. A.; Bang J.; Hara T.; Saito S.; Zambetti G. P.; Appella D. H. A New Family of Small Molecules To Probe the Reactivation of Mutant p53. J. Am. Chem. Soc. 2005, 127 (17), 6152–6153. 10.1021/ja045752y. 15853303

[ref6] Foley K. F.; Cozzi N. V. Novel aminopropiophenones as potential antidepressants. Drug Dev. Res. 2003, 60 (4), 252–260. 10.1002/ddr.10297.

[ref7] Kolanos R.; Partilla J. S.; Baumann M. H.; Hutsell B. A.; Banks M. L.; Negus S. S.; Glennon R. A. Stereoselective Actions of Methylenedioxypyrovalerone (MDPV) To Inhibit Dopamine and Norepinephrine Transporters and Facilitate Intracranial Self-Stimulation in Rats. ACS Chem. Neurosci. 2015, 6, 771–777. 10.1021/acschemneuro.5b00006. 25688761 PMC5349769

[ref8] Hou F. F.; Zhang X.; Zhang G. H.; Xie D.; Chen P. Y.; Zhang W. R.; Jiang J. P.; Liang M.; Wang G. B.; Liu Z. R.; et al. Efficacy and Safety of Benazepril for Advanced Chronic Renal Insufficiency. N. Engl. J. Med. 2006, 354 (2), 131–140. 10.1056/NEJMoa053107. 16407508

[ref9] Sham H. L.; Kempf D. J.; Molla A.; Marsh K. C.; Kumar G. N.; Chen C. M.; Kati W.; Stewart K.; Lal R.; Hsu A.; et al. ABT-378, a Highly Potent Inhibitor of the Human Immunodeficiency Virus Protease. Antimicrob. Agents Chemother. 1998, 42 (12), 3218–3224. 10.1128/aac.42.12.3218. 9835517 PMC106025

[ref10] Du Y.; Li Q.; Xiong B.; Hui X.; Wang X.; Feng Y.; Meng T.; Hu D.; Zhang D.; Wang M. Aromatic β-amino-ketone derivatives as novel selective non-steroidal progesterone receptor antagonists. Bioorg. Med. Chem. 2010, 18 (12), 4255–4268. 10.1016/j.bmc.2010.04.092. 20510622

[ref11] Makarova N.; Boreko E.; Moiseev I.; Pavlova N.; Zemtsova M.; Nikolaeva S.; Vladyko G. Antiviral Activity of Adamantyl-Containing β-Aminoketones, Enaminoketones, and Related Compounds. Pharm. Chem. J. 2001, 35 (9), 480–484. 10.1023/A:1014086507352.

[ref12] Altmeyer M.; Amtmann E.; Heyl C.; Marschner A.; Scheidig A. J.; Klein C. D. Beta-aminoketones as prodrugs for selective irreversible inhibitors of type-1 methionine aminopeptidases. Bioorg. Med. Chem. Lett. 2014, 24, 5310–5314. 10.1016/j.bmcl.2014.09.047. 25293447

[ref13] Perrine D. M.; Ross J. T.; Nervi S. J.; Zimmerman R. H. A Short, One-Pot Synthesis of Bupropion (Zyban, Wellbutrin). J. Chem. Educ. 2000, 77 (11), 1479 10.1021/ed077p1479.

[ref14] Silverstone T. Appetite Suppressants. Drugs 1992, 43, 820–836. 10.2165/00003495-199243060-00003. 1379155

[ref15] Nchinda T.; Chibale K.; Redelinghuys P.; Sturrock E. D. Synthesis of novel keto-ACE analogues as domain-selective angiotensin I-converting enzyme inhibitors. Bioorg. Med. Chem. Lett. 2006, 16 (17), 4612–4615. 10.1016/j.bmcl.2006.06.003. 16784850

[ref16] Almquist R. G.; Chao W.-R.; Ellis M. E.; Johnson H. L. Synthesis and biological activity of a ketomethylene analog of a tripeptide inhibitor of angiotensin converting enzyme. J. Med. Chem. 1980, 23 (12), 1392–1398. 10.1021/jm00186a020. 6256550

[ref17] Shan J.; Sun H. The discovery and development of prasugrel. Expert Opin. Drug Discovery 2013, 8 (7), 897–905. 10.1517/17460441.2013.793668. 23614463

[ref18] Krechetov S.; Nifontova G.; Dolotova O.; Veselov M. Solubility and Stability of Proroxan at Various PH Values. Pharm. Chem. J. 2018, 52, 236–240. 10.1007/s11094-018-1798-1.

[ref19] Nifontova G.; Krechetov S.; Dolotova O.; Buyukli S.; Akhmetzyanova A.; Krasnyuk I. Granulation of Effervescent Ingredients for Optimization of Gastroretentive Properties of Floating Proroxan Prolonged-Release Tablets. Pharm. Chem. J. 2018, 52, 361–365. 10.1007/s11094-018-1822-5.

[ref20] Hofer D.; Lohberger B.; Steinecker B.; Schmidt K.; Quasthoff S.; Schreibmayer W. A. A comparative study of the action of tolperisone on seven different voltage dependent sodium channel isoforms. Eur. J. Pharmacol. 2006, 538, 5–14. 10.1016/j.ejphar.2006.03.034. 16650844

[ref21] Kaski J. C.; Araujo L.; Maseri A. Effects of oxyfedrine on regional myocardial blood flow in patients with coronary artery disease. Cardiovasc. Drugs Ther. 1991, 5, 991–996. 10.1007/BF00143526. 1801897

[ref22] Concellón J. M.; Rodríguez-Solla H. Synthesis and Synthetic Applications of α-Amino Ketones Derived from Natural α-Amino Acids. Curr. Org. Chem. 2008, 12 (7), 524–543. 10.2174/138527208784245996.

[ref23] Nguyen N. H.; Hughes A. B.; Sleebs B. E. Stereoselective Synthesis and Application of β-Amino Ketones. Curr. Org. Chem. 2014, 18 (2), 260–289. 10.2174/138527281802140129104218.

[ref24] Deacon C. F. Dipeptidyl peptidase 4 inhibitors in the treatment of type 2 diabetes mellitus. Nat. Rev. Endocrinol. 2020, 16, 642–653. 10.1038/s41574-020-0399-8. 32929230

[ref25] Langer P.; Bodtke A. Sequential cyclizations of 2-isothiocyanatobenzonitrile and 2-isocyanatobenzonitrile with α-aminoketones. Tetrahedron Lett. 2003, 44 (32), 5965–5967. 10.1016/S0040-4039(03)01489-8.

[ref26] Sorrell T. N.; Allen W. E. A regiospecific synthesis of 1,4-disubstituted imidazoles. J. Org. Chem. 1994, 59 (6), 1589–1590. 10.1021/jo00085a056.

[ref27] Frantz D. E.; Morency L.; Soheili A.; Murry J. A.; Grabowski E. J. J.; Tillyer R. D. Synthesis of Substituted Imidazoles via Organocatalysis. Org. Lett. 2004, 6 (5), 843–846. 10.1021/ol0498803. 14986989

[ref28] Huang J.; Luo L.; Xing N.; Gu L.; Li C.; Han Q.; Zheng S.; He L. Novel synthesis of divergent aryl imidazoles from ketones involving copper-catalyzed α-amination and oxidative C–C bond cleavage. RSC Adv. 2020, 10, 13815–13819. 10.1039/D0RA01408G. 35492973 PMC9051655

[ref29] Iida H.; Hayashida K.; Yamada M.; Takahashi K.; Yamada K. Synthesis of 2,5-Dimethylpyrazine-3,6-Dicarboxylic Acid Derivatives. Synth. Commun. 1973, 3 (3), 225–230. 10.1080/00397917308062041.

[ref30] Chiba T.; Sakagami H.; Murata M.; Okimoto M. Electrolytic Oxidation of Ketones in Ammoniacal Methanol in the Presence of Catalytic Amounts of KI. J. Org. Chem. 1995, 60 (21), 6764–6770. 10.1021/jo00126a027.

[ref31] Knorr L. Synthese von Pyrrolderivaten. Ber. Dtsch. Chem. Ges. 1884, 17 (2), 1635–1642. 10.1002/cber.18840170220.

[ref32] Knorr L. Synthetische Versuche mit dem Acetessigester. II. Mittheilung: Ueberführung des Diacetbernsteinsäureesters und des Acetessigesters in Pyrrolderivate. Adv. Cycloaddit. 1886, 236 (3), 290–332. 10.1002/jlac.18862360303.

[ref33] Klingler F. D. Asymmetric Hydrogenation of Prochiral Amino Ketones to Amino Alcohols for Pharmaceutical Use. Acc. Chem. Res. 2007, 40 (12), 1367–1376. 10.1021/ar700100e. 18052332

[ref34] Gediya S. K.; Clarkson G. J.; Wills M. Asymmetric Transfer Hydrogenation: Dynamic Kinetic Resolution of α-Amino Ketones. J. Org. Chem. 2020, 85 (17), 11309–11330. 10.1021/acs.joc.0c01438. 32786626

[ref35] Wu W.; You C.; Yin C.; Liu Y.; Dong X. Q.; Zhang X. Enantioselective and Diastereoselective Construction of Chiral Amino Alcohols by Iridium–f-Amphox-Catalyzed Asymmetric Hydrogenation via Dynamic Kinetic Resolution. Org. Lett. 2017, 19 (10), 2548–2551. 10.1021/acs.orglett.7b00844. 28448152

[ref36] Ager D. J.; Prakash I.; Schaad D. R. 1,2-Amino Alcohols and Their Heterocyclic Derivatives as Chiral Auxiliaries in Asymmetric Synthesis. Chem. Rev. 1996, 96 (2), 835–876. 10.1021/cr9500038. 11848773

[ref37] Bartoli G.; Cimarelli C.; Palmieri G. Convenient procedure for the reduction of β-enamino ketones: synthesis of γ-amino alcohols and tetrahydro-1,3-oxazines. J. Chem. Soc., Perkin Trans. 1994, 1 (5), 537–543. 10.1039/P19940000537.

[ref38] Bates R. W.; Sa-Ei K. Syntheses of the sedum and related alkaloids. Tetrahedron 2002, 58 (30), 5957–5978. 10.1016/S0040-4020(02)00584-7.

[ref39] Davis F. A.; Prasad K. R.; Nolt M. B.; Wu Y. *N*-Sulfinyl β-Amino Weinreb Amides: Synthesis of Enantiopure β-Amino Carbonyl Compounds. Asymmetric Synthesis of (+)-Sedridine and (−)-Allosedridine. Org. Lett. 2003, 5 (6), 925–927. 10.1021/ol034119z. 12633107

[ref40] Jefford C. W.; Wang J. B. An enantiospecific synthesis of solenopsin A. Tetrahedron Lett. 1993, 34 (18), 2911–2914. 10.1016/S0040-4039(00)60479-3.

[ref41] Keck G. E.; Truong A. P. Directed Reduction of β-Amino Ketones to Syn or Anti 1,3-Amino Alcohol Derivatives. Org. Lett. 2002, 4 (18), 3131–3134. 10.1021/ol026456y. 12201734 PMC1480404

[ref42] Wanner K. T.; Höfner G. Chelat- und nicht-chelat-kontrollierte reduktionen von β-amido-ketonen: Synthese nicht-racemischer 1,3-aminoalkohole mit pyrrolidinstruktur. Tetrahedron 1991, 47 (10), 1895–1910. 10.1016/S0040-4020(01)96102-2.

[ref43] Barluenga J.; Tomas M.; Kouznetsov V.; Jardon J.; Rubio E. Reduction of the Pyrimidine Ring: Regio- and Stereoselective Synthesis of 1,3-Diamine Derivatives. Synlett 1991, 1991 (11), 821–823. 10.1055/s-1991-20890.

[ref44] Couty F.; David O.; Durrat F.; Evano G.; Lakhdar S.; Marrot J.; Vargas-Sanchez M. Nucleophilic Ring-Opening of Azetidinium Ions: Insights into Regioselectivity. Eur. J. Org Chem. 2006, 2006 (15), 3479–3490. 10.1002/ejoc.200600200.

[ref45] Shustov G.; Denisenko S.; Chervin I.; Asfandiarov N.; Kostyanovsky R. Asymmetric nitrogen—41: Stereochemistry of bicyclic 1,2-cis-diaziridines. Tetrahedron 1985, 41 (23), 5719–5731. 10.1016/S0040-4020(01)91378-X.

[ref46] Colyer J. T.; Andersen N. G.; Tedrow J. S.; Soukup T. S.; Faul M. M. Reversal of Diastereofacial Selectivity in Hydride Reductions of *N*-tert-Butanesulfinyl Imines. J. Org. Chem. 2006, 71 (18), 6859–6862. 10.1021/jo0609834. 16930038

[ref47] Denmark S. E.; Weber T.; Piotrowski D. W. Organocerium additions to SAMP-hydrazones: general synthesis of chiral amines. J. Am. Chem. Soc. 1987, 109 (7), 2224–2225. 10.1021/ja00241a073.

[ref48] González-Sabín J.; Gotor V.; Rebolledo F. CAL-B-catalyzed resolution of some pharmacologically interesting β–substituted isopropylamines. Tetrahedron: Asymmetry 2002, 13 (12), 1315–1320. 10.1016/S0957-4166(02)00336-1.

[ref49] Nechab M.; Azzi N.; Vanthuyne N.; Bertrand M.; Gastaldi S.; Gil G. Highly Selective Enzymatic Kinetic Resolution of Primary Amines at 80 °C: A Comparative Study of Carboxylic Acids and Their Ethyl Esters as Acyl Donors. J. Org. Chem. 2007, 72 (18), 6918–6923. 10.1021/jo071069t. 17676806

[ref50] C Cole D. Recent stereoselective synthetic approaches to β-amino acids. Tetrahedron 1994, 50 (32), 9517–9582. 10.1016/S0040-4020(01)85527-7.

[ref51] Liu M.; Sibi M. P. Recent advances in the stereoselective synthesis of β-amino acids. Tetrahedron 2002, 58 (40), 7991–8035. 10.1016/S0040-4020(02)00991-2.

[ref52] Davis F. A.; Song M.; Qiu H.; Chai J. Total synthesis of (5*R*,6*R*,8*R*,9*S*)-(−)-5,9*Z*-indolizidine 221T using sulfinimine-derived *N*-sulfinyl β-amino ketones. Org. Biomol. Chem. 2009, 7 (24), 5067–5073. 10.1039/b915796d. 20024099

[ref53] Klix R. C.; Chamberlin S. A.; Bhatia A. V.; Davis D. A.; Hayes T. K.; Rojas F. G.; Koops R. W. A practical, large-scale procedure for the preparation of N-protected α-amino ketones from α-amino acids. Tetrahedron Lett. 1995, 36 (11), 1791–1794. 10.1016/0040-4039(95)00144-2.

[ref54] Florjancic A.; Sheppard G. S. A Practical Synthesis of α-Amino Ketones via Aryllithium Addition to N-Boc-α-Amino Acids. Synthesis 2003, 11, 1653–1656. 10.1055/s-2003-40875.

[ref55] De Luca L.; Giacomelli G.; Porcheddu A. A. Simple Preparation of Ketones. N-Protected α-Amino Ketones from α-Amino Acids. Org. Lett. 2001, 3, 1519–1521. 10.1021/ol015840c. 11388856

[ref56] Zhou Z. H.; Tang Y. L.; Li K. Y.; Liu B.; Tang C. C. Synthesis of optically active N-protected α-aminoketones and α-amino alcohols. Heteroat. Chem. 2003, 14, 603–606. 10.1002/hc.10195.

[ref57] Lv Y.; Li Y.; Xiong T.; Lu Y.; Liu Q.; Zhang Q. *n*Bu_4_NI-Catalyzed oxidative imidation of ketones with imides: synthesis of α-amino ketones. Chem. Commun. 2014, 50, 2367–2369. 10.1039/c3cc48887j. 24448553

[ref58] Buckley T. F. III; Rapoport H. .alpha.-Amino acids as chiral educts for asymmetric products. Amino acylation with N-acylamino acids. J. Am. Chem. Soc. 1981, 103 (20), 6157–6163. 10.1021/ja00410a030.

[ref59] McClure D. E.; Arison B. H.; Jones J. H.; Baldwin J. J. Chiral.alpha.-amino ketones from the Friedel-Crafts reaction of protected amino acids. J. Org. Chem. 1981, 46 (11), 2431–2433. 10.1021/jo00324a057.

[ref60] McClure D. E.; Lumma P. K.; Arison B. H.; Jones J. H.; Baldwin J. J. 1,4-Oxazines via intramolecular ring closure of.beta.-hydroxydiazoacetamides: phenylalanine to tetrahydroindeno[1,2-b]-1,4-oxazin-3(2H)-ones. J. Org. Chem. 1983, 48 (16), 2675–2679. 10.1021/jo00164a009.

[ref61] Nordlander J. E.; Payne M. J.; Njoroge F. G.; Balk M. A.; Laikos G. D.; Vishwanath V. M. Friedel-Crafts acylation with N-(trifluoroacetyl)-.alpha.-amino acid chlorides. Application to the preparation of.beta.-arylalkylamines and 3-substituted 1,2,3,4-tetrahydroisoquinolines. J. Org. Chem. 1984, 49 (22), 4107–4111. 10.1021/jo00196a001.

[ref62] Nordlander J. E.; Njoroge F. G.; Payne M. J.; Warman D. N-(Trifluoroacetyl)-.alpha.-amino acid chlorides as chiral reagents for Friedel-Crafts synthesis. J. Org. Chem. 1985, 50 (19), 3481–3484. 10.1021/jo00219a012.

[ref63] Itoh O.; Honnami T.; Amano A.; Murata K.; Koichi Y.; Sugita T. Friedel-Crafts.alpha.-aminoacylation of alkylbenzene with a chiral N-carboxy-.alpha.-amino acid anhydride without loss of chirality. J. Org. Chem. 1992, 57 (26), 7334–7338. 10.1021/jo00052a059.

[ref64] Di Gioia M. L.; Leggio A.; Liguori A.; Napoli A.; Siciliano C.; Sindona G. Facile Approach to Enantiomerically Pure α-Amino Ketones by Friedel–Crafts Aminoacylation and Their Conversion into Peptidyl Ketones. J. Org. Chem. 2001, 66 (21), 7002–7007. 10.1021/jo010414q. 11597220

[ref65] Katritzky A. R.; Jiang R.; Suzuki K. N-Tfa- and N-Fmoc-(α-aminoacyl)benzotriazoles as Chiral C-Acylating Reagents under Friedel–Crafts Reaction Conditions. J. Org. Chem. 2005, 70 (13), 4993–5000. 10.1021/jo050226q. 15960497

[ref66] Plażuk D.; Zakrzewski J. Acylation of Ferrocene and a 1,1′-Diphosphaferrocene with Acyl Trifluoroacetates in the Presence of Trifluoromethanesulfonic (Triflic) Acid or Some Metal Triflates. Synth. Commun. 2004, 34 (1), 99–107. 10.1081/SCC-120027243.

[ref67] Plażuk D.; Zakrzewski J. Friedel–Crafts acylation of ferrocene with alkynoic acids. J. Organomet. Chem. 2009, 694 (12), 1802–1806. 10.1016/j.jorganchem.2009.01.007.

[ref68] Plażuk D.; Zakrzewski J.; Salmain M. Biotin as acylating agent in the Friedel–Crafts reaction. Avidin affinity of biotinyl derivatives of ferrocene, ruthenocene and pyrene and fluorescence properties of 1-biotinylpyrene. Org. Biomol. Chem. 2011, 9, 408–417. 10.1039/C0OB00319K. 20967359

[ref69] Plażuk D.; Zakrzewski J.; Salmain M.; Błauż A.; Rychlik B.; Strzelczyk P.; Bujacz A.; Bujacz G. Ferrocene–Biotin Conjugates Targeting Cancer Cells: Synthesis, Interaction with Avidin, Cytotoxic Properties and the Crystal Structure of the Complex of Avidin with a Biotin–Linker–Ferrocene Conjugate. Organometallics 2013, 32 (20), 5774–5783. 10.1021/om4003126.

[ref70] Murai Y.; Wang L.; Muto Y.; Sakihama Y.; Hashidoko Y.; Hatanaka Y.; Hashimoto M. Simple and Stereocontrolled Preparation of Benzoylated Phenylalanine Using Friedel–Crafts Reaction in Trifluoromethanesulfonic Acid for Photoaffinity Labeling. Heterocycles 2013, 87 (10), 2119–2126. 10.3987/COM-13-12815.

[ref71] Murashige R.; Hayashi Y.; Ohmori S.; Torii A.; Aizu Y.; Muto Y.; Murai Y.; Oda Y.; Hashimoto M. Comparisons of O-acylation and Friedel–Crafts acylation of phenols and acyl chlorides and Fries rearrangement of phenyl esters in trifluoromethanesulfonic acid: effective synthesis of optically active homotyrosines. Tetrahedron 2011, 67 (3), 641–649. 10.1016/j.tet.2010.11.047.

[ref72] Murai Y.; Hashidoko Y.; Hashimoto M. Novel Synthesis of Optically Active Bishomotyrosine Derivatives Using the Friedel-Crafts Reaction in Triflic Acid. Biosci., Biotechnol., Biochem. 2011, 75 (2), 352–354. 10.1271/bbb.100595. 21307587

[ref73] Wuts P. G. M. Greene’s Protective Groups in Organic Synthesis, 5th ed.; John Wiley & Sons, Inc., Hoboken, NJ, 2014; pp 993–994

[ref74] Carpino L. A. The 9-fluorenylmethyloxycarbonyl family of base-sensitive amino-protecting groups. Acc. Chem. Res. 1987, 20 (11), 401–407. 10.1021/ar00143a003.

[ref75] Anderson K. W.; Tepe J. J. Trifluoromethanesulfonic acid catalyzed Friedel–Crafts acylation of aromatics with β-lactams. Tetrahedron 2002, 58 (42), 8475–8481. 10.1016/S0040-4020(02)01026-8.

[ref76] Newman H. Trifluoroacetyl as a Protecting Group for 1-Halo Sugars. J. Org. Chem. 1965, 30 (4), 1287–1288. 10.1021/jo01015a533.

[ref77] Boger D. L.; Yohannes D. Total synthesis of K-13. J. Org. Chem. 1989, 54 (11), 2498–2502. 10.1021/jo00272a003.

[ref78] Tarbell D. S.; Yamamoto Y.; Pope B. M. New Method to Prepare N-t-Butoxycarbonyl Derivatives and the Corresponding Sulfur Analogs from di-t-Butyl Dicarbonate or di-t-Butyl Dithiol Dicarbonates and Amino Acids. Proc. Natl. Acad. Sci. U.S.A. 1972, 69 (3), 730–732. 10.1073/pnas.69.3.730. 16591972 PMC426545

[ref79] Stahl G. L.; Walter R.; Smith C. W. General procedure for the synthesis of mono-N-acylated 1,6-diaminohexanes. J. Org. Chem. 1978, 43 (11), 2285–2286. 10.1021/jo00405a045.

[ref80] Martin C. L.; Nakamura S.; Otte R.; Overman L. E. Total Synthesis of (+)-Condylocarpine, (+)-Isocondylocarpine, and (+)-Tubotaiwine. Org. Lett. 2011, 13 (1), 138–141. 10.1021/ol102709s. 21133399 PMC3014433

[ref81] Etter M. C.; MacDonald J. C.; Bernstein J. Graph-set analysis of hydrogen-bond patterns in organic crystals. Acta Crystallogr. 1990, 46, 256–262. 10.1107/S0108768189012929. 2344397

[ref82] Fulmer G. R.; Miller A. J. M.; Sherden N. H.; Gottlieb H. E.; Nudelman A.; Stoltz B. M.; Bercaw J. E.; Goldberg K. I. NMR Chemical Shifts of Trace Impurities: Common Laboratory Solvents, Organics, and Gases in Deuterated Solvents Relevant to the Organometallic Chemist. Organometallics 2010, 29 (9), 2176–2179. 10.1021/om100106e.

[ref83] Dales N.; Zhang Z.; Kamboj R.; Fu J.; Sun S.; Pokrovskaia N.; Sviridov S. Heterocyclic organic compounds. WO 2008036715 A1, 2008.

[ref84] Sheldrick G. M. A short history of SHELX. Acta Crystallogr. 2008, 64, 112–122. 10.1107/S0108767307043930. 18156677

[ref85] Sheldrick G. M. SHELXT – Integrated space-group and crystal-structure determination. Acta Crystallogr. 2015, 71, 3–8. 10.1107/S2053273314026370. PMC4283466 25537383

[ref86] Dolomanov O. V.; Bourhis L. J.; Gildea R. J.; Howard J. A. K.; Puschmann H. OLEX2: a complete structure solution, refinement and analysis program. J. Appl. Crystallogr. 2009, 42, 339–341. 10.1107/S0021889808042726.

