# Supplementary material for: Synthesis of Trifluoroacetamidoketones by Acylation of Ferrocene with In Situ Protected Amino Acids
Source: J Org Chem. 2025 Feb 18;90(8):2958–68. doi: 10.1021/acs.joc.4c02717 (PMC11877514; doi:10.1021/acs.joc.4c02717)
Supplement: Supplementary file 1 — jo4c02717_si_001.pdf [file jo4c02717_si_001.pdf]

Supporting Information

Synthesis of trifluoroacetamidoketones by acylation of ferrocene with *in situ* protected amino acids

Michał Piotrowicz,*[a] Natasza Masłowska, [a] Róża Dziewiątkowska, [b] Anna Makal,[b] and Bogna Rudolf [a]

[a]University of Lodz, Faculty of Chemistry, Department of Organic Chemistry, Tamka 12, 91-403 Lodz, Poland.

[b]Biological and Chemical Research Centre, Faculty of Chemistry, University of Warsaw, Żwirki i Wigury 101, 02-089 Warszawa, Poland.

Corresponding author: michal.piotrowicz@chemia.uni.lodz.pl

Table of contents

X-ray diffraction data S5

Table S1. The summary of crystal data and data processing and structure refinement statistics for structures of **3a-g** and **11**. S5

Crystal structure refinement details S6

Selected geometrical parameters for described structures S6

Table S2. Selected bond lengths for described compounds in [Å]. S6

Table S3. Selected interatomic torsion angles for described compounds in [°]. S7

Table S4. Selected geometric parameters describing ferrocene moieties – distances in [Å], torsion angles in [°]. S7

Table S5. Selected geometric parameters describing hydrogen bonds in [Å]/[°]. (D) refer to molecule acting as a donor and (A) – as an acceptor of hydrogen bond. S7

3a: S8

3b: S8

3c: S9

3d: S9

3e: S10

3f: S10

3g: S11

11: S11

Figure S1. Molecular structures of characterized compounds in ORTEP representation with the atom-numbering scheme (labels of H atoms omitted for clarity). Atomic Displacement Parameters represented at 50% probability level. S11

Figure S2. Hydrogen bond motifs in described structures: a) branched chain in **3a**, b) dimer in **3c**, c) non-parallel chain in **3g**, d) parallel chain in **3f**, e) chain in **11**. S12

Figure S3. Overlay of molecular structures **3a** – **3g**, performed so as the Fe ion and the substituted cyclopnetadienyl ring achieve the closest fit. Structure **3f**, the only one where the alkyl chain deviates out of the cyclpentadienyl plate, represented in green, the remaining structures in light gray. S13

NMR and IR spectra of 3a – g, 10 and 11 .... S14

Figure S4. ^{1}H NMR spectrum of **3a** in $CDCl_3$ (600 MHz). S14

Figure S5. $^{13}C\{^{1}H\}$ NMR spectrum of **3a** in $CDCl_3$ (151 MHz). S15

Figure S6. ^{19}F NMR spectrum of **3a** in $CDCl_3$ (565 MHz). S16

Figure S7. FT-IR spectrum of **3a**. S17

Figure S8. ^{1}H NMR spectrum of **3b** in $CDCl_3$ (600 MHz). S18

Figure S9. $^{13}C\{^{1}H\}$ NMR spectrum of **3b** in $CDCl_3$ (151 MHz). S19

Figure S10. ^{19}F NMR spectrum of **3b** in $CDCl_3$ (565 MHz). S20

Figure S11. FT-IR spectrum of **3b**. S21
Figure S12. ^{1}H NMR spectrum of **3c** in $CDCl_3$ (600 MHz). S22
Figure S13. $^{13}C\{^{1}H\}$ NMR spectrum of **3c** in $CDCl_3$ (151 MHz). S23
Figure S14. ^{19}F NMR spectrum of **3c** in $CDCl_3$ (565 MHz). S24
Figure S15. FT-IR spectrum of **3c**. S25
Figure S16. ^{1}H NMR spectrum of **3d** in $CDCl_3$ (600 MHz). S26
Figure S17. $^{13}C\{^{1}H\}$ NMR spectrum of **3d** in $CDCl_3$ (151 MHz). S27
Figure S18. ^{19}F NMR spectrum of **3d** in $CDCl_3$ (565 MHz). S28
Figure S19. FT-IR spectrum of **3d**. S29
Figure S20. ^{1}H NMR spectrum of **3e** in $CDCl_3$ (600 MHz). S30
Figure S21. $^{13}C\{^{1}H\}$ NMR spectrum of **3e** in $CDCl_3$ (151 MHz). S31
Figure S22. ^{19}F NMR spectrum of **3e** in $CDCl_3$ (565 MHz). S32
Figure S23. FT-IR spectrum of **3e**. S33
Figure S24. ^{1}H NMR spectrum of **3f** in $CDCl_3$ (600 MHz). S34
Figure S25. $^{13}C\{^{1}H\}$ NMR spectrum of **3f** in $CDCl_3$ (151 MHz). S35
Figure S26. ^{19}F NMR spectrum of **3f** in $CDCl_3$ (565 MHz). S36
Figure S27. FT-IR spectrum of **3f**. S37
Figure S28. ^{1}H NMR spectrum of **3g** in $CDCl_3$ (600 MHz). S38
Figure S29. $^{13}C\{^{1}H\}$ NMR spectrum of **3g** in $CDCl_3$ (151 MHz). S39
Figure S30. ^{19}F NMR spectrum of **3g** in $CDCl_3$ (565 MHz). S40
Figure S31. FT-IR spectrum of **3g**. S41
Figure S32. ^{1}H NMR spectrum of **10** in CD_3OD (600 MHz). S42
Figure S33. $^{13}C\{^{1}H\}$ NMR spectrum of **10** in CD_3OD (600 MHz). S43
Figure S34. FT-IR spectrum of **10**. S44
Figure S35. HRMS spectrum of **10**. S45
Figure S36. ^{1}H NMR spectrum of **11** in $CDCl_3$ (600 MHz). S46
Figure S37. $^{13}C\{^{1}H\}$ NMR spectrum of **11** in $CDCl_3$ (151 MHz). S47
Figure S38. FT-IR spectrum of **11**. S48
NMR and HRMS spectra of 8a – g and 9. .... S49
Figure S39. ^{1}H NMR spectrum of **8a** in CD_2Cl_2 (600 MHz). S49
Figure S40. $^{13}C\{^{1}H\}$ NMR spectrum of **8a** in CD_2Cl_2 (151 MHz). S50
Figure S41. ^{19}F NMR spectrum of **8a** in CD_2Cl_2 (565 MHz). S51
Figure S42. HRMS spectrum of **8a**. S51
Figure S43. ^{1}H NMR spectrum of **8b** in CD_2Cl_2 (600 MHz). S52
Figure S44. $^{13}C\{^{1}H\}$ NMR spectrum of **8b** in CD_2Cl_2 (151 MHz). S53
Figure S45. ^{19}F NMR spectrum of **8b** in CD_2Cl_2 (565 MHz). S54

Figure S46. HRMS spectrum of **8b**. S54
Figure S47. ^{1}H NMR spectrum of **8c** and **9** (mixture) in CD_2Cl_2 (600 MHz). S55
Figure S48. $^{13}C\{^{1}H\}$ NMR spectrum of **8c** and **9** (mixture) in CD_2Cl_2 (151 MHz). S56
Figure S49. ^{19}F NMR spectrum of **8c** and **9** (mixture) in CD_2Cl_2 (565 MHz). S57
Figure S50. HRMS spectrum of **8c**. S57
Figure S51. HRMS spectrum of **9**. S58
Figure S52. ^{1}H NMR spectrum of **8d** in CD_2Cl_2 (600 MHz). S59
Figure S53. $^{13}C\{^{1}H\}$ NMR spectrum of **8d** in CD_2Cl_2 (151 MHz). S60
Figure S54. ^{19}F NMR spectrum of **8d** in CD_2Cl_2 (565 MHz). S61
Figure S55. HRMS spectrum of **8d**. S61
Figure S56. ^{1}H NMR spectrum of **8e** in CD_2Cl_2 (600 MHz). S62
Figure S57. $^{13}C\{^{1}H\}$ NMR spectrum of **8e** in CD_2Cl_2 (151 MHz). S63
Figure S58. ^{19}F NMR spectrum of **8e** in CD_2Cl_2 (565 MHz). S64
Figure S59. HRMS spectrum of **8e**. S64
Figure S60. ^{1}H NMR spectrum of **8f** in CD_2Cl_2 (600 MHz). S66
Figure S61. $^{13}C\{^{1}H\}$ NMR spectrum of **8f** in CD_2Cl_2 (151 MHz). S66
Figure S62. ^{19}F NMR spectrum of **8f** in CD_2Cl_2 (565 MHz). S67
Figure S63. HRMS spectrum of **8f**. S67
Figure S64. ^{1}H NMR spectrum of **8g** in CD_2Cl_2 (600 MHz). S68
Figure S65. $^{13}C\{^{1}H\}$ NMR spectrum of **8g** in CD_2Cl_2 (151 MHz). S69
Figure S66. ^{19}F NMR spectrum of **8g** in CD_2Cl_2 (565 MHz). S70
Figure S67. HRMS spectrum of **8g**. S70
Figure S68. ^{1}H NMR spectrum of **trifluoroacetic acid** in CD_2Cl_2 (600 MHz). S71
Figure S69. $^{13}C\{^{1}H\}$ NMR spectrum of **trifluoroacetic acid** in CD_2Cl_2 (151 MHz). S72
Figure S70. ^{19}F NMR spectrum of **trifluoroacetic acid** in CD_2Cl_2 (565 MHz). S73
Figure S71. $^{13}C\{^{1}H\}$ NMR spectrum of **trifluoroacetic anhydride** in CD_2Cl_2 (151 MHz). S74
Figure S72. ^{19}F NMR spectrum of **trifluoroacetic anhydride** in CD_2Cl_2 (565 MHz). S75

X-ray diffraction data

Table S1. The summary of crystal data and data processing and structure refinement statistics for structures of **3a-g** and **11**.

Code	**3a**	**3b**	**3c**	**3d**	**3e**	**3f**	**3g**	**11**
Formula	$C_{14}H_{12}F_3FeNO_2$	$C_{15}H_{14}F_3FeNO_2$	$C_{16}H_{16}F_3FeNO_2$	$C_{17}H_{18}F_3FeNO_2$	$C_{18}H_{20}F_3FeNO_2$	$C_{23}H_{30}F_3FeNO_2$	$C_{24}H_{32}F_3FeNO_2$	$C_{18}H_{23}FeNO_3$
Weight [g/mol]	339.10	353.12	367.15	381.17	395.20	465.33	479.35	357.22
Temperature [K]	100.01(10)	100.01(10)	120.15	119.99(12)	100.00(10)	119.97(14)	120.15	100.00(10)
Crystal system	orthorhombic	triclinic	monoclinic	monoclinic	triclinic	monoclinic	monoclinic	triclinic
Space Group	P 21 21 21	P -1	P 1 21/n 1	C 1 2/c 1	P -1	P 1 21 1	C 1 2/c 1	P -1
a [Å]	5.62565(10)	7.1589(2)	7.16750(10)	20.0865(2)	10.2204(3)	7.2624(2)	41.548(2)	5.57770(10)
b [Å]	20.9077(4)	9.8772(2)	10.2208(2)	8.84720(10)	10.7458(4)	7.7676(4)	10.8280(10)	9.5650(2)
c [Å]	34.5282(5)	10.0729(2)	20.8188(3)	18.5201(2)	15.7078(2)	19.5523(8)	10.2777(15)	15.6422(4)
α [°]	90	81.241(2)	90	90	89.708(2)	90	90	96.603(2)
β [°]	90	88.123(2)	97.6070(10)	101.3570(10)	89.302(2)	98.896(4)	95.961(10)	90.324(2)
ɣ [°]	90	78.347(2)	90	90	87.672(3)	90	90	98.564(2)
Volume [$Å^3$]	4061.19(13)	689.44(3)	1511.71(4)	3226.75(6)	1723.56(8)	1089.72(8)	4598.8(8)	819.52(3)
Z	12	2	4	8	4	2	8	2
ϱ_{calc} [g/cm^3]	1.664	1.701	1.613	1.569	1.523	1.418	1.385	1.448
μ [mm^{-1}]	1.152	9.176	8.394	7.888	7.405	5.940	5.646	7.497
F(000)	2064	360	752	1568	816	488	2016	376
Crystal size [mm^3]	0.39 x 0.12 x 0.07	0.43 x 0.23 x 0.1	0.801 x 0.324 x 0.017	0.26 x 0.16 x 0.12	0.32 x 0.21 x 0.07	0.26 x 0.09 x 0.02	0.335 x 0.094 x 0.02	0.3 x 0.18 x 0.4
Radiation source	Mo Kα	Cu Kα	CuKα	CuKα	Cu Kα	CuKα	CuKα	CuKα
Wavelength [Å]	0.71073	1.54184	1.54184	1.54184	1.54184	1.54184	1.54184	1.54184
2Θ range	3.06 to 56.162	8.882 to 155.25	8.57 to 155.306	8.98 to 155.548	8.234 to 156.306	9.156 to 157.044	8.442 to 156.048	9.416 to 156.458
Reflections #	20808	7765	19646	26249	34574	17455	12009	18841

R_{int}	0.0273	0.0521	0.0443	0.0432	0.0561	0.0843	0.146	0.0760
Resolution [Å]	0.7549	0.7893	0.7892	0.7888	0.7877	0.7867	0.7881	0.7875
Completeness	0.999	0.999	1.000	1.000	0.998	0.995	0.997	0.998
Data; restr.; param.	8967; 36; 740	2913; 0; 199	3211; 48; 240	3420; 0; 221	7228; 0; 455	4371; 85; 263	12009; 126; 281	3421; 0; 215
R_1 (I > 2 σ(I))	0.0285	0.0394	0.0285	0.0361	0.0845	0.1426	0.2059	0.0389
wR_2 (I > 2 σ(I))	0.0652	0.1023	0.0681	0.0955	0.2706	0.3696	0.4726	0.0986
R_1 (all data)	0.0301	0.0409	0.0344	0.0379	0.0880	0.1467	0.2550	0.0430
wR_2 (all data)	0.0659	0.1039	0.0710	0.0969	0.2723	0.3735	0.5048	0.1022
$\Delta\varrho_{max}$ [eÅ^{-3}]	0.313	0.651	0.308	0.821	2.377	3.063	3.591	1.131
$\Delta\varrho_{min}$ [eÅ^{-3}]	-0.492	-0.584	-0.197	-0.582	-0.579	-1.282	-0.799	-0.446
Flack parameter	0.017(7)					0.14(3)		

Crystal structure refinement details

A dynamic disorder in CF_3 group was observed in structures of **3a** and **3c** with a refined second variant content of 11% and 44%, respectively. Restrains used for disordered atoms from CF_3 moiety refer to the unification of C – F bond lengths, angles between them and atomic displacement parameters of fluorine atoms in both variants. In the case of **3f** and **3g** some restraints were used for carbon atoms in ferrocene in order to model their thermal motions as similar (treating cyclopentadienyl rings as rigid objects). Structure of **3g** was solved and refined against twinned data. Consequently, some restraints were used for carbon atoms of aliphatic chain to avoid non-physical atomic displacement parameters. Hydrogen atoms in amide group, which are involved in hydrogen bonds, were refined freely in structures of **3a-e**, **11** (because they location was possible to find on the Fourier difference map) and constrained in **3f-g**. The remaining hydrogen atoms in all structures were placed in idealized positions.

Selected geometrical parameters for described structures

Table S2. Selected bond lengths for described compounds in [Å].

	**3a**			**3b**	**3c**	**3d**	**3e**		**3f**	**3g**	**11**
	I	II	III				I	II			
C1 – C11	1.458(4)	1.461(4)	1.473(4)	1.472(3)	1.472(2)	1.476(3)	1.483(8)	1.470(8)	1.46(2)	1.475(19)	1.473(3)
C11 – O1	1.224(4)	1.221(4)	1.216(4)	1.230(2)	1.230(2)	1.225(2)	1.218(8)	1.221(8)	1.25(2)	1.21(2)	1.224(3)

Table S3. Selected interatomic torsion angles for described compounds in [°].

	3a			3b	3c	3d	3e		3f	3g	11
	I	II	III				I	II			
C5 – C1 – C11 – O1	11.7(5)	7.4(5)	179.1(3)	1.8(3)	8.8(3)	5.7(3)	2.1(9)	-1.9(10)	4(3)	-1(2)	178.4(2)
C1 – C11 – C12 – C13	-	-	-	161.3(2)	-176.3(1)	-173.5(2)	-173.0(5)	-178.1(15)	-75(2)	-178(1)	-166.7(2)
C(12+n) – N – C(11+n) – C(10+n)	-77.5(4)	-73.7(4)	69.4(4)	-88.2(2)	-150.3(2)	76.9(2)	-87.6(7)	168.2(5)	172(2)	178(2)	82.1(3)

Table S4. Selected geometric parameters describing ferrocene moieties – distances in [Å], torsion angles in [°].

	3a			3b	3c	3d	3e		3f	3g	11
	I	II	III				I	II			
Fe … Cp1	1.650	1.656	1.654	1.647	1.648	1.647	1.649	1.645	1.627	1.644	1.648
Fe … Cp2	1.666	1.658	1.658	1.657	1.656	1.653	1.649	1.648	1.635	1.637	1.652
Cp1 … Cp2	3.316	3.314	3.312	3.303	3.304	3.300	3.297	3.292	3.262	3.280	3.300
C1 … Cp1 … Cp2 … C6	-5.61	1.83	1.40	-7.18	-1.50	0.14	-2.43	-4.14	-23.13	-2.07	6.47

Table S5. Selected geometric parameters describing hydrogen bonds in [Å]/[°]. (D) refer to molecule acting as a donor and (A) – as an acceptor of hydrogen bond.

	3a			3b	3c	3d	3e		3f	3g	11
	I(D) - I(A)	II(D) - I(A)	III(D) - II(A)				I(D) - II(A)	II(D) - I(A)			
N - H	0.83(4)	0.82(4)	0.78(4)	0.77(4)	0.83(2)	0.83(3)	0.96(9)	0.85(6)	0.88 (fixed)	0.88 (fixed)	0.79(3)
H ··· O	2.09(4)	2.10(4)	2.13(4)	2.08(4)	2.09(2)	2.04(3)	1.95(8)	2.09(6)	2.08	2.08	2.70(3)
N ··· O	2.832(3)	2.839(4)	2.888(4)	2.835(2)	2.906(2)	2.835(2)	2.847(7)	2.915(7)	2.93(2)	2.90(2)	3.429(3)
N - H ··· O	149(3)	150(4)	167(4)	166(4)	169(2)	160(3)	155(7)	163(6)	164	155	153(3)

3a:

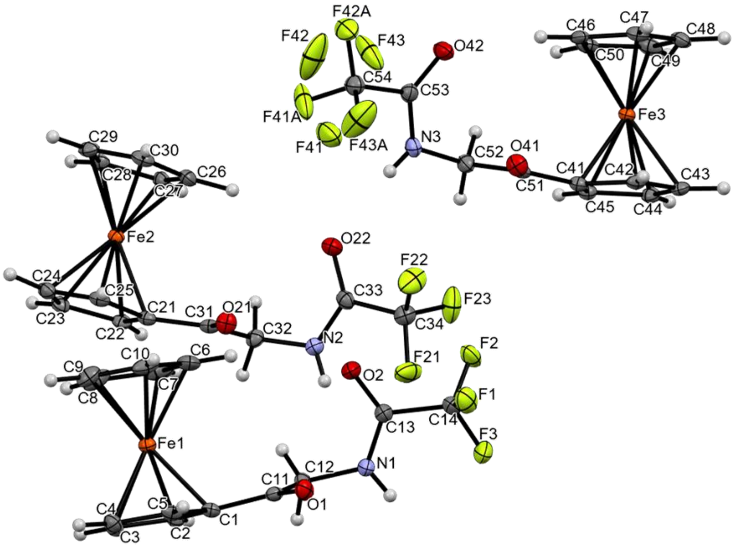
F42A
F42
F43
O42
C46
C47
C48
C50
C49
C54
C53
F41A
Fe3
F41
F43A
N3
C52
O41
C41
C42
C43
C51
C45
C44
C29
C30
C28
C26
C27
Fe2
O22
F22
C33
F23
C24
C25
C34
C21
C31
O21
C23
C32
C22
N2
F2
C10
C6
F21
C9
O2
C7
F1
C8
C14
C13
F3
Fe1
C12
N1
C11
C4
C5
O1
C3
C2
C1

3b:

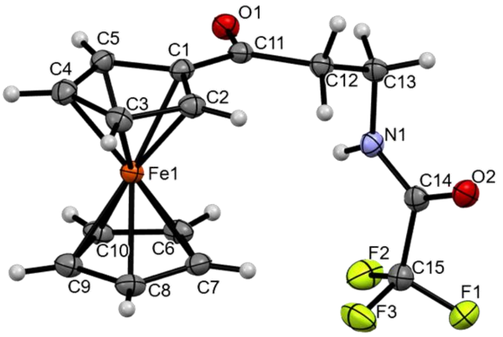
O1
C5
C1
C11
C4
C12
C13
C3
C2
N1
Fe1
C14
O2
C10
C6
C15
F2
C9
C8
C7
F1
F3

3c:

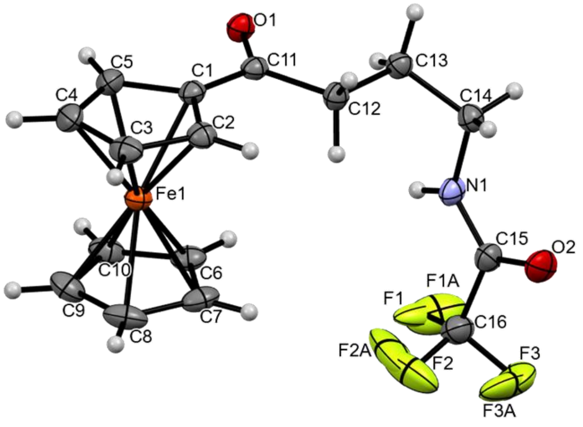
O1
C5
C11
C13
C1
C4
C14
C12
C2
C3
Fe1
N1
C15
O2
C10
C6
F1A
F1
C16
C9
C7
C8
F2A
F2
F3
F3A

3d:

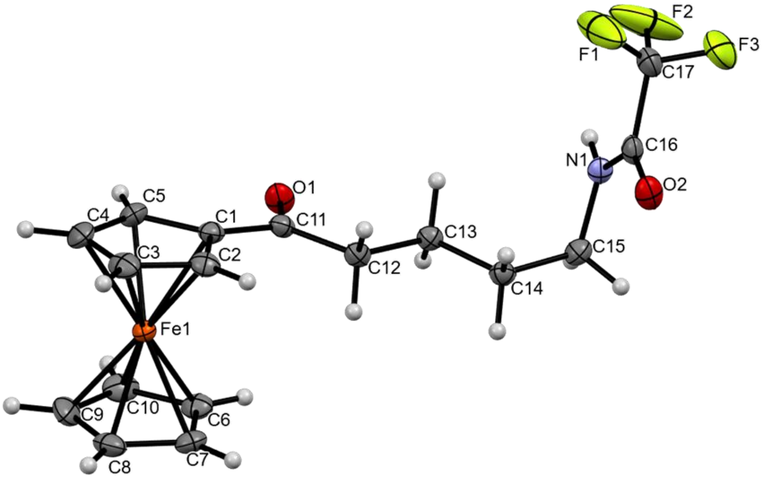
F2
F3
F1
C17
C16
N1
O2
O1
C5
C1
C4
C11
C13
C3
C2
C15
C12
C14
Fe1
C9
C10
C6
C8
C7

3e:

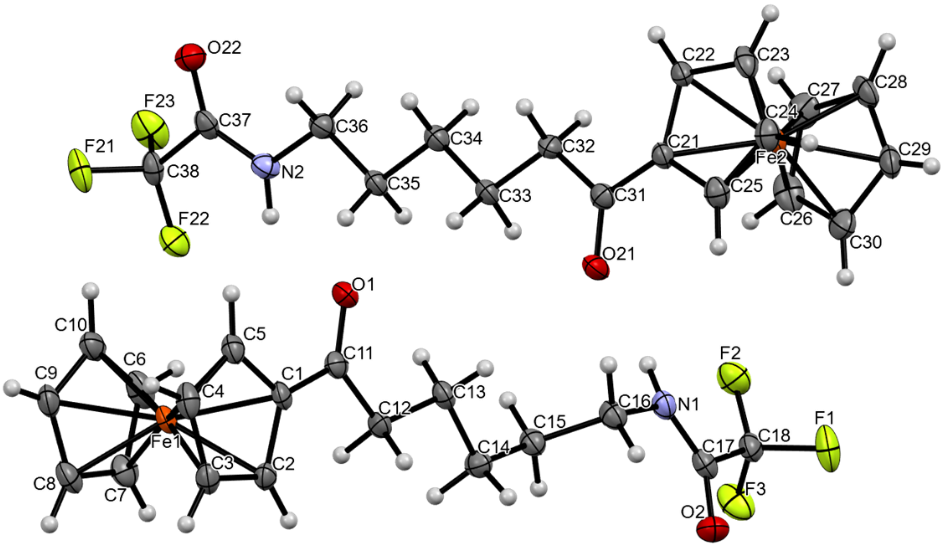
O22
F23
C37
F21
C38
N2
F22
C36
C34
C35
C33
C32
C31
O21
C21
C22
C23
C27
C28
C24
Fe2
C29
C25
C26
C30
O1
C10
C5
C6
C9
C4
C1
C11
C13
C12
Fe1
C16
N1
F2
C15
C14
C17
C18
F1
C8
C7
C3
C2
F3
O2

3f:

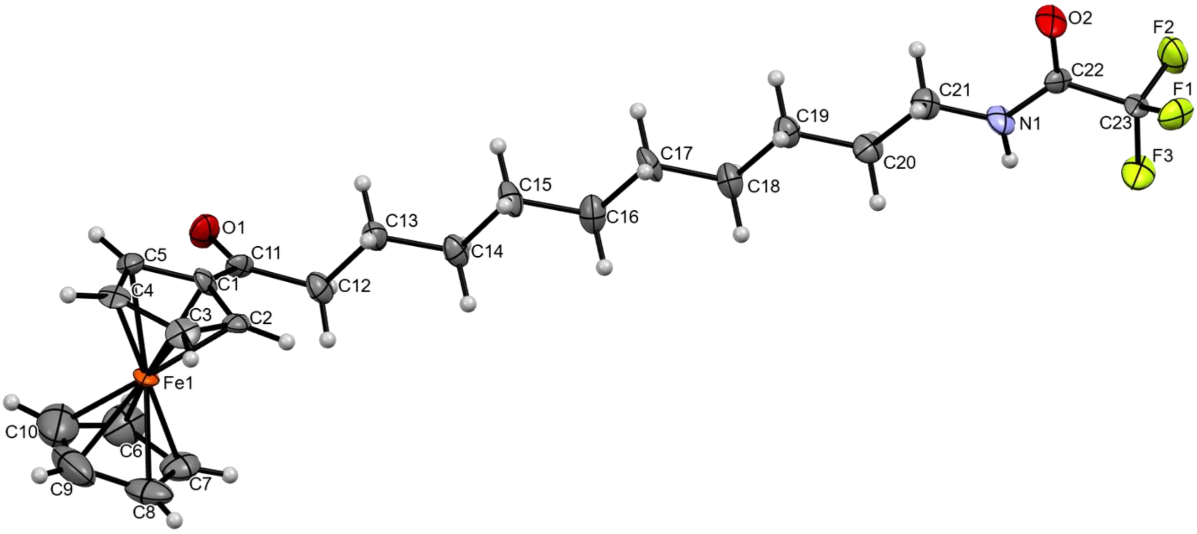
O2
F2
C22
F1
C21
N1
C23
F3
C19
C20
C17
C18
C15
C16
C13
C14
O1
C11
C5
C4
C1
C12
C3
C2
Fe1
C10
C6
C9
C7
C8

3g:

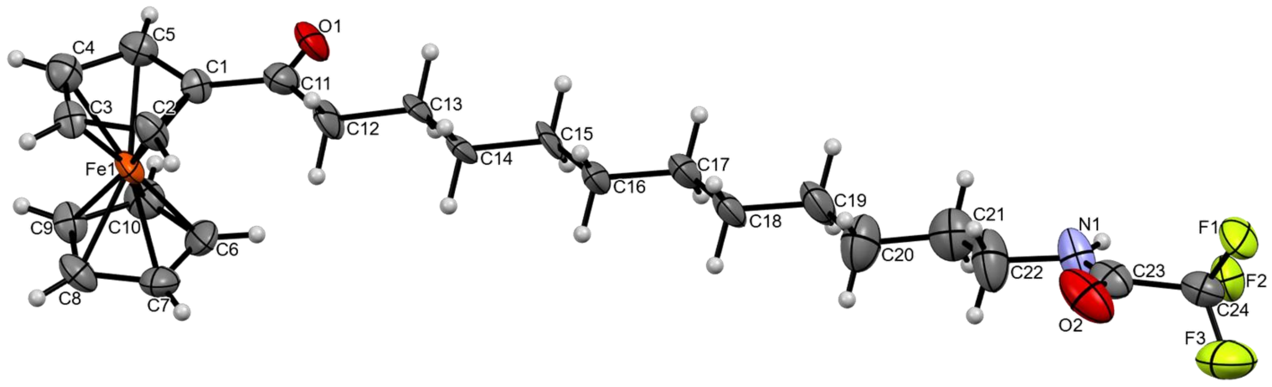

11:

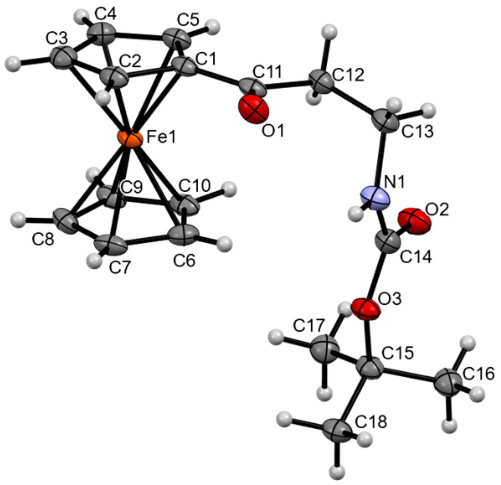

Figure S1. Molecular structures of characterized compounds in ORTEP representation with the atom-numbering scheme (labels of H atoms omitted for clarity). Atomic Displacement Parameters represented at 50% probability level.

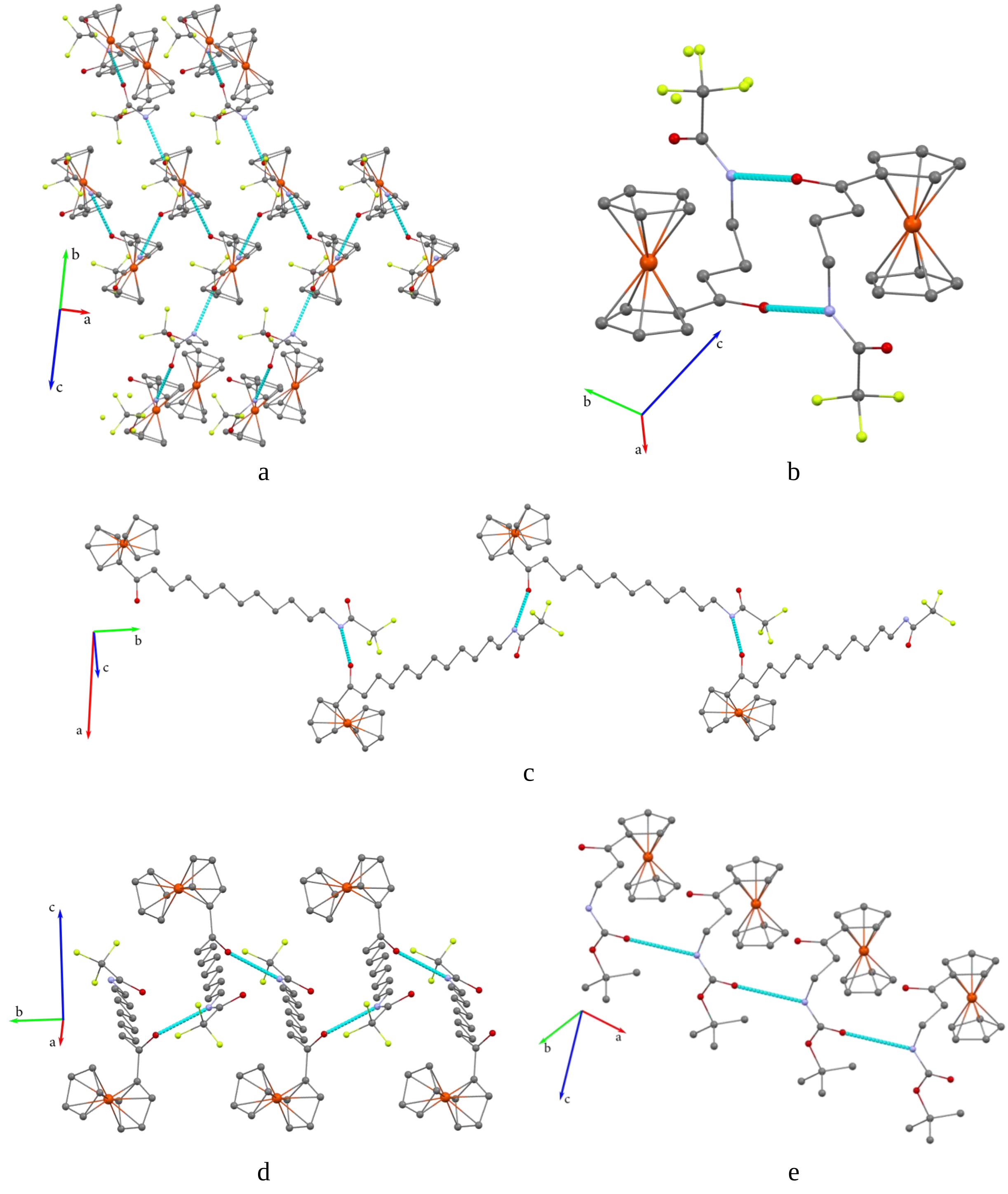

Figure S2. Hydrogen bond motifs in described structures: a) branched chain in **3a**, b) dimer in **3c**, c) non-parallel chain in **3g**, d) parallel chain in **3f**, e) chain in **11**.

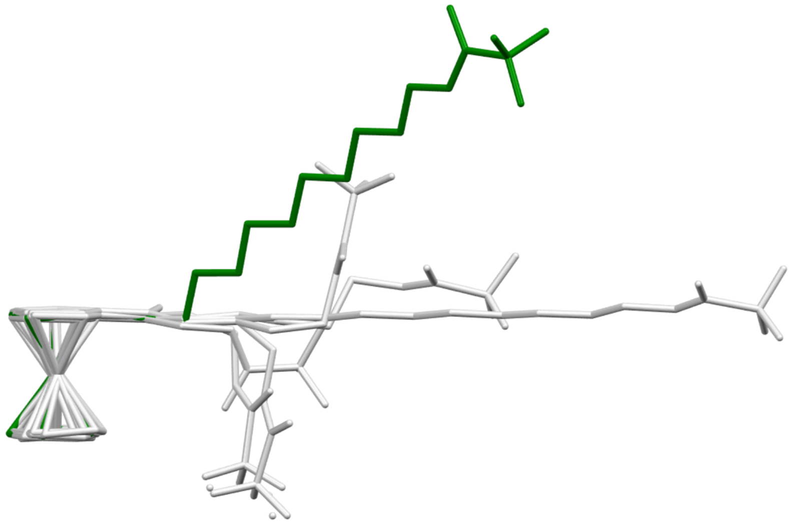

Figure S3. Overlay of molecular structures **3a** – **3g**, performed so as the Fe ion and the substituted cyclopnetadienyl ring achieve the closest fit. Structure **3f**, the only one where the alkyl chain deviates out of the cyclpentadienyl plate, represented in green, the remaining structures in light gray.

NMR and IR spectra of 3a – g, 10 and 11.

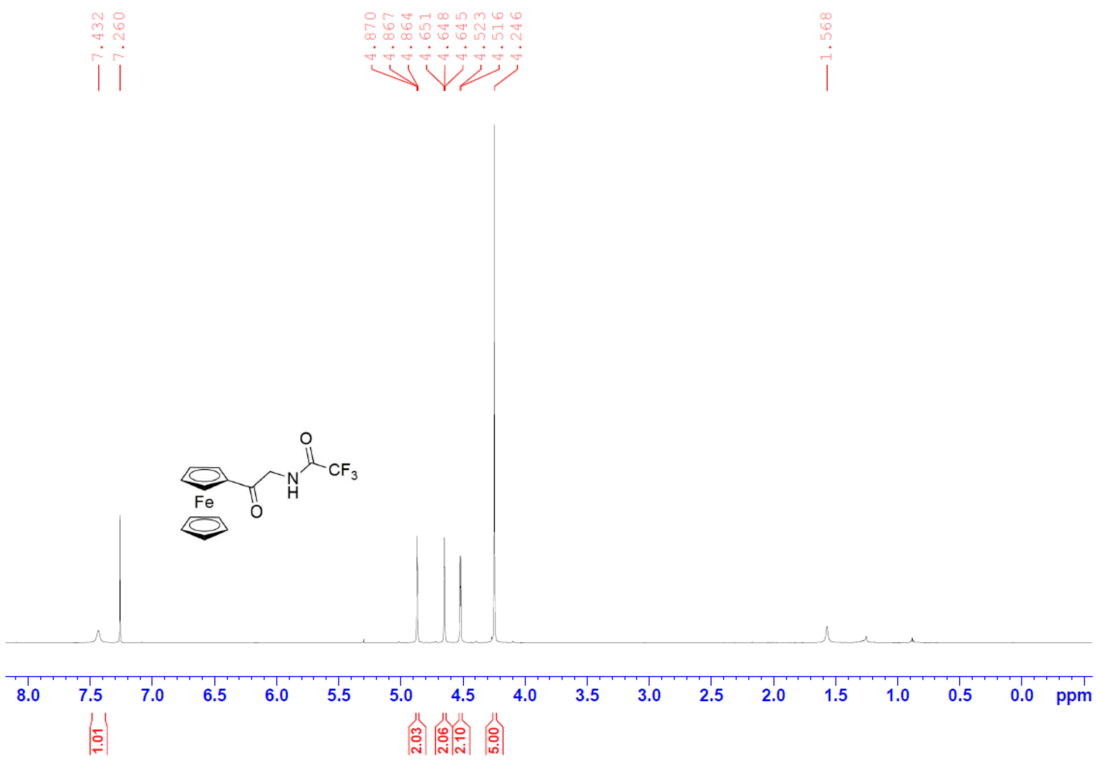

Figure S4. ^{1}H NMR spectrum of **3a** in $CDCl_3$ (600 MHz).

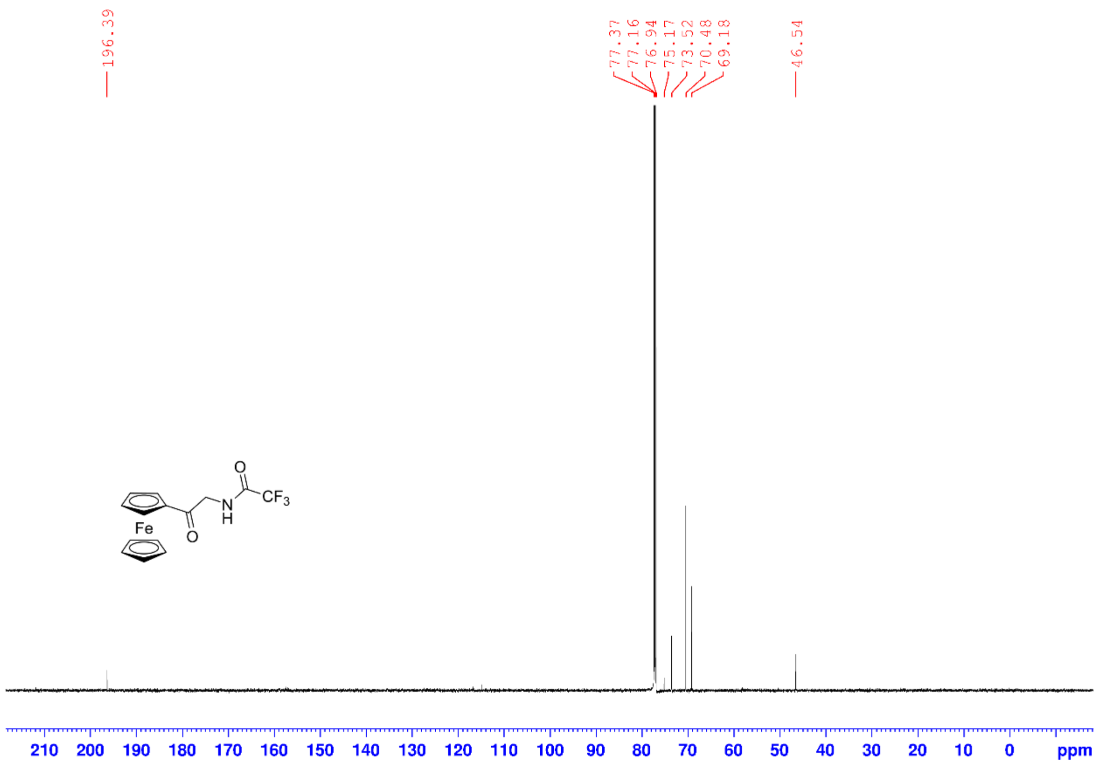

Figure S5. $^{13}C\{^{1}H\}$ NMR spectrum of **3a** in $CDCl_3$ (151 MHz).

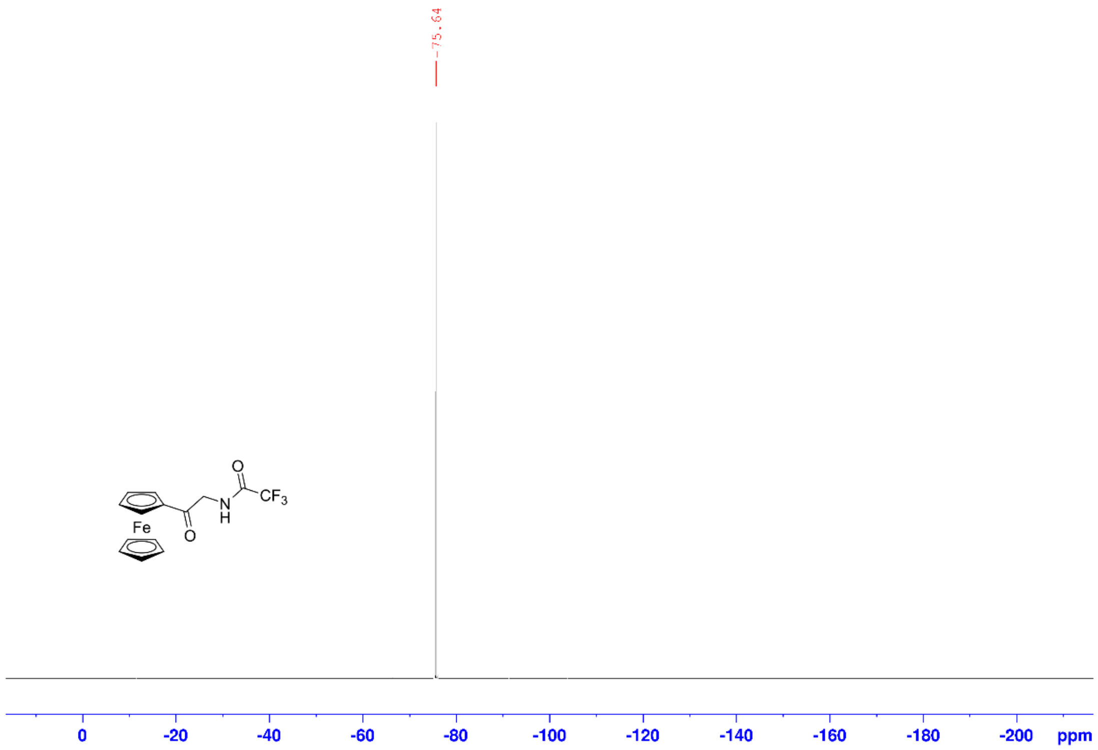

Figure S6. ^{19}F NMR spectrum of **3a** in $CDCl_3$ (565 MHz).

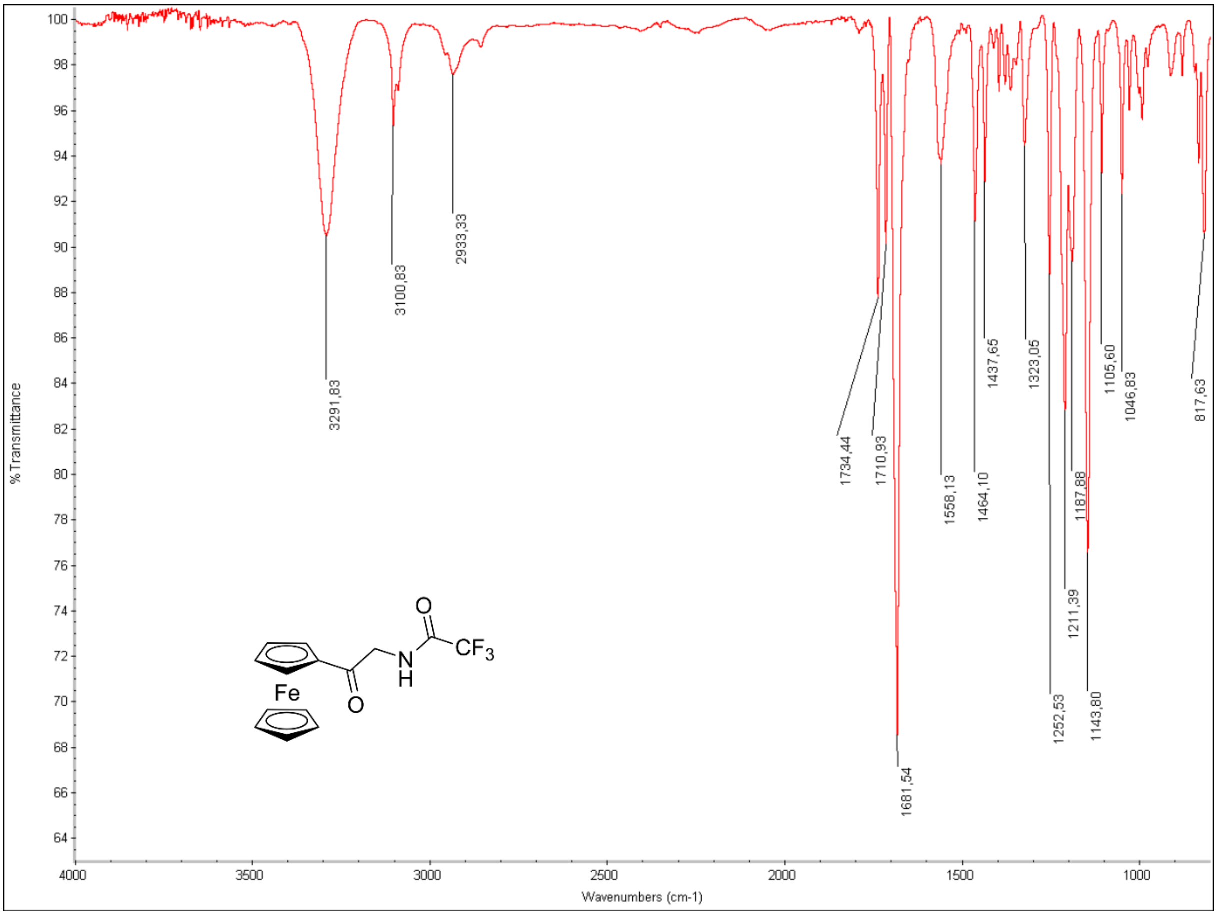

Figure S7. FT-IR spectrum of **3a**.

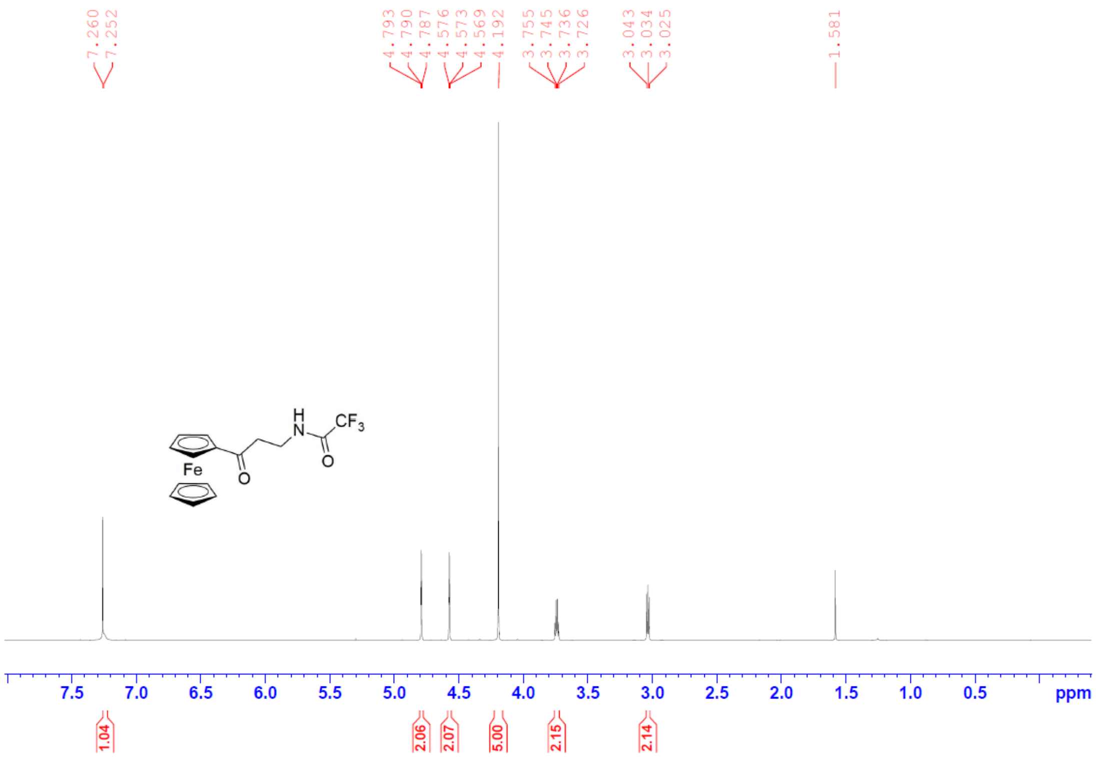

Figure S8. ^{1}H NMR spectrum of **3b** in $CDCl_3$ (600 MHz).

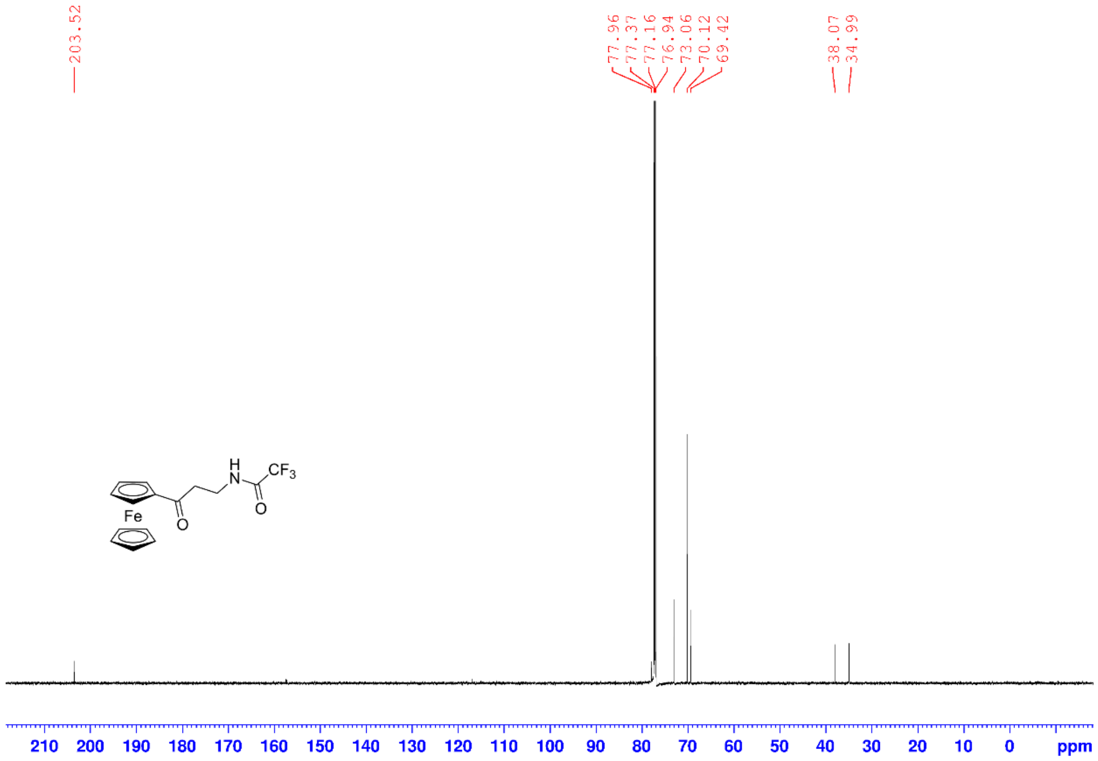

Figure S9. $^{13}C\{^{1}H\}$ NMR spectrum of **3b** in $CDCl_3$ (151 MHz).

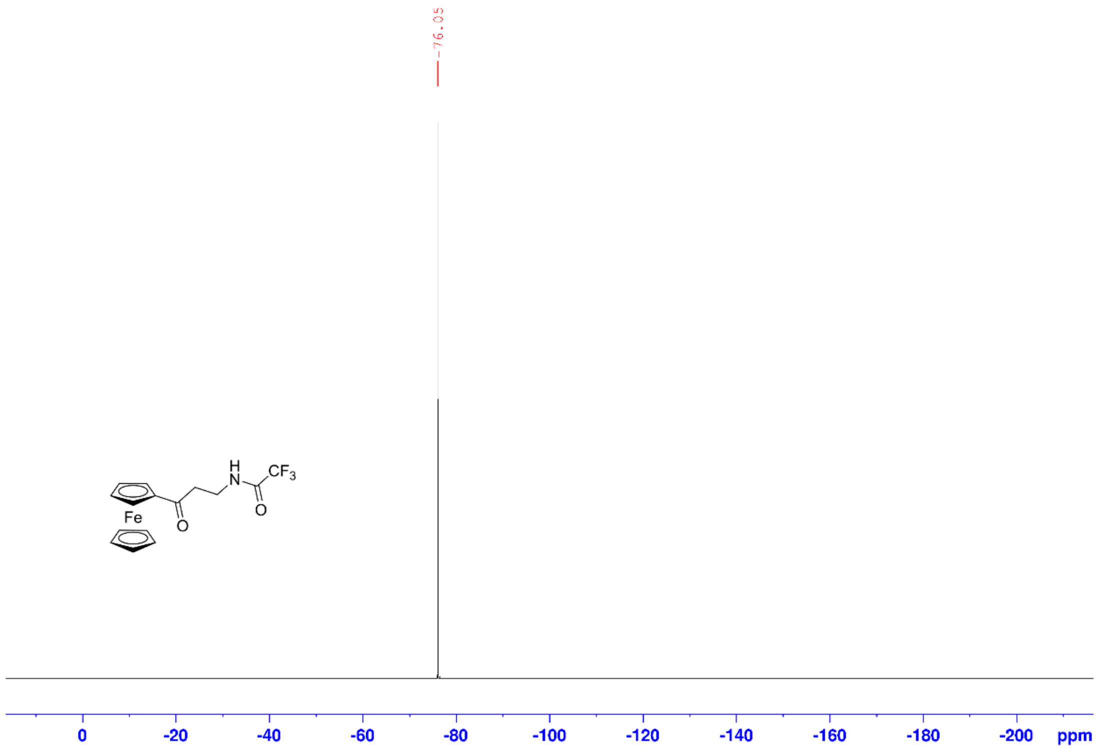

Figure S10. ^{19}F NMR spectrum of **3b** in $CDCl_3$ (565 MHz).

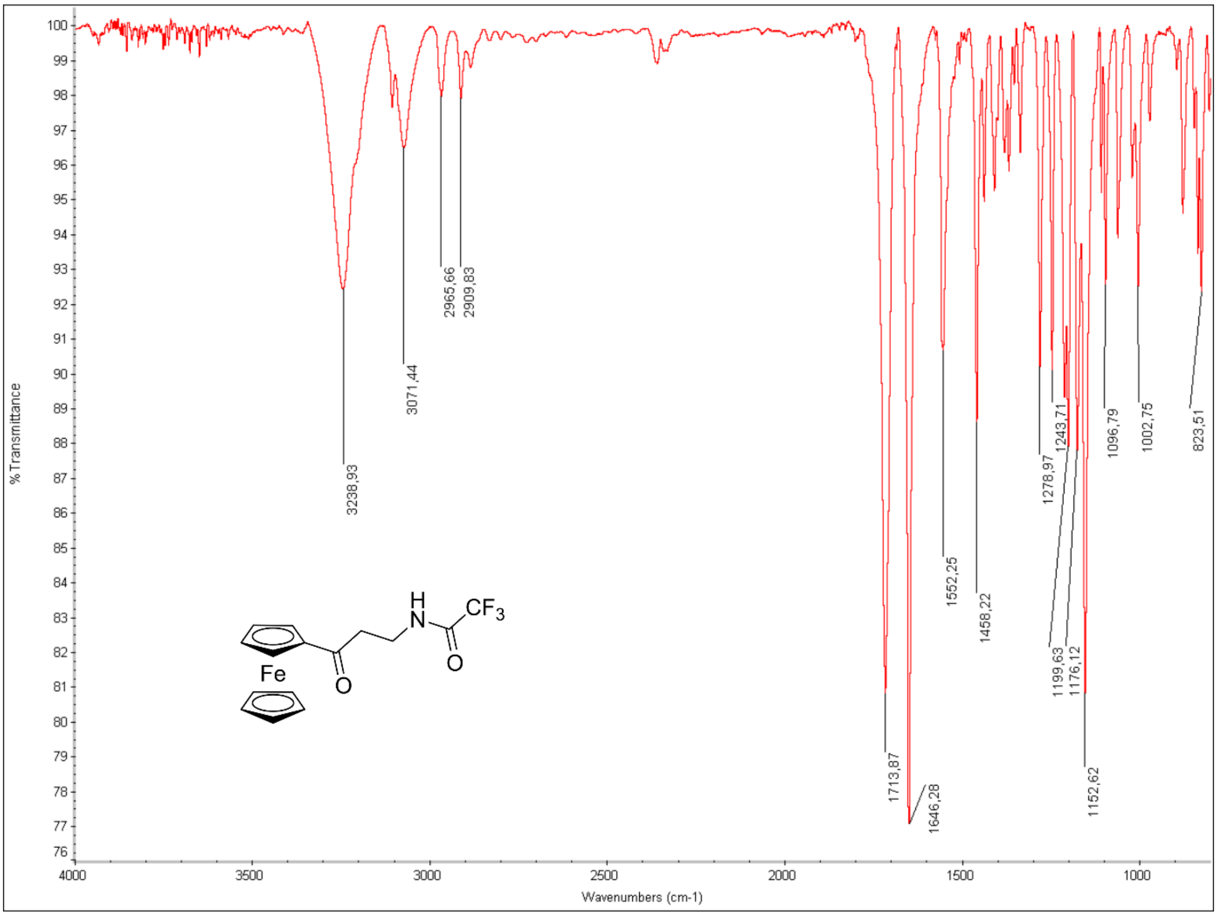

Figure S11. FT-IR spectrum of **3b**.

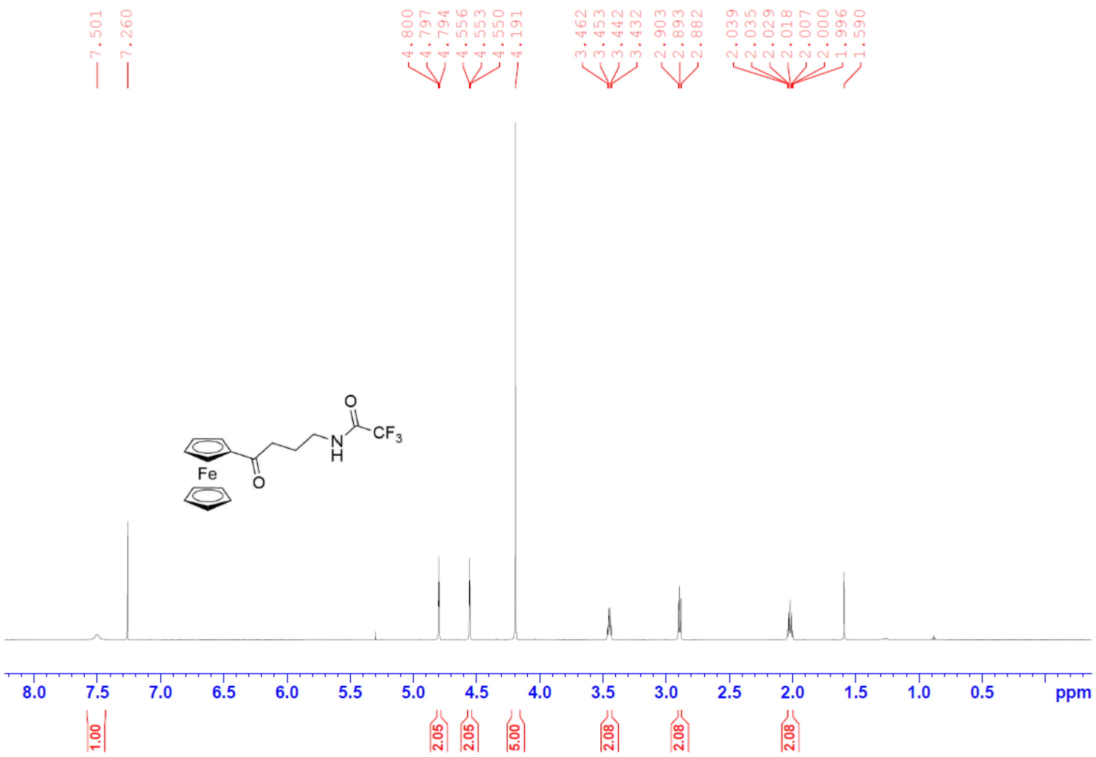

Figure S12. ^{1}H NMR spectrum of **3c** in $CDCl_3$ (600 MHz).

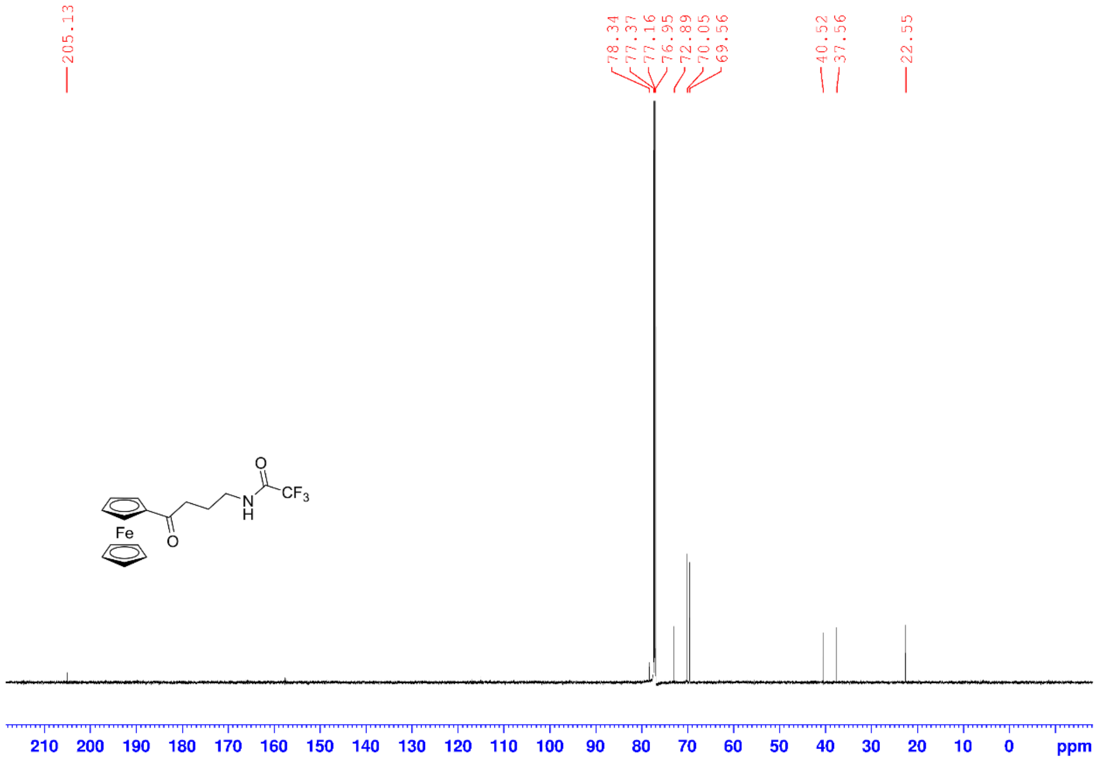

Figure S13. $^{13}C\{^{1}H\}$ NMR spectrum of **3c** in $CDCl_3$ (151 MHz).

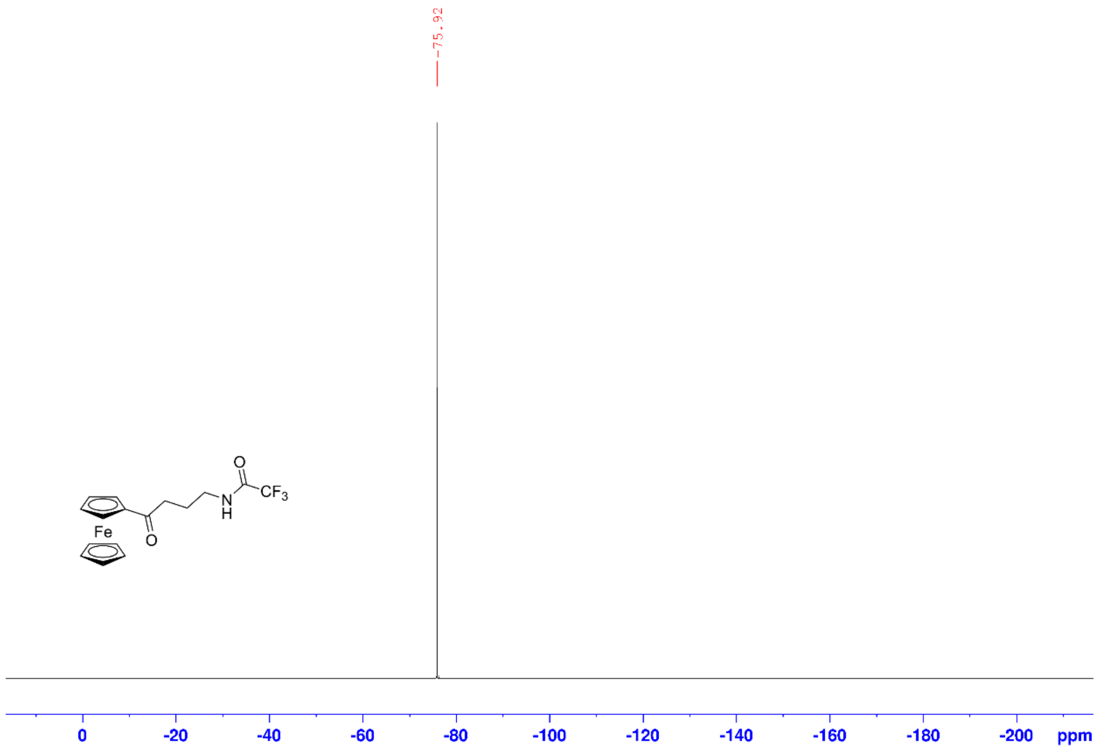

Figure S14. ^{19}F NMR spectrum of **3c** in $CDCl_3$ (565 MHz).

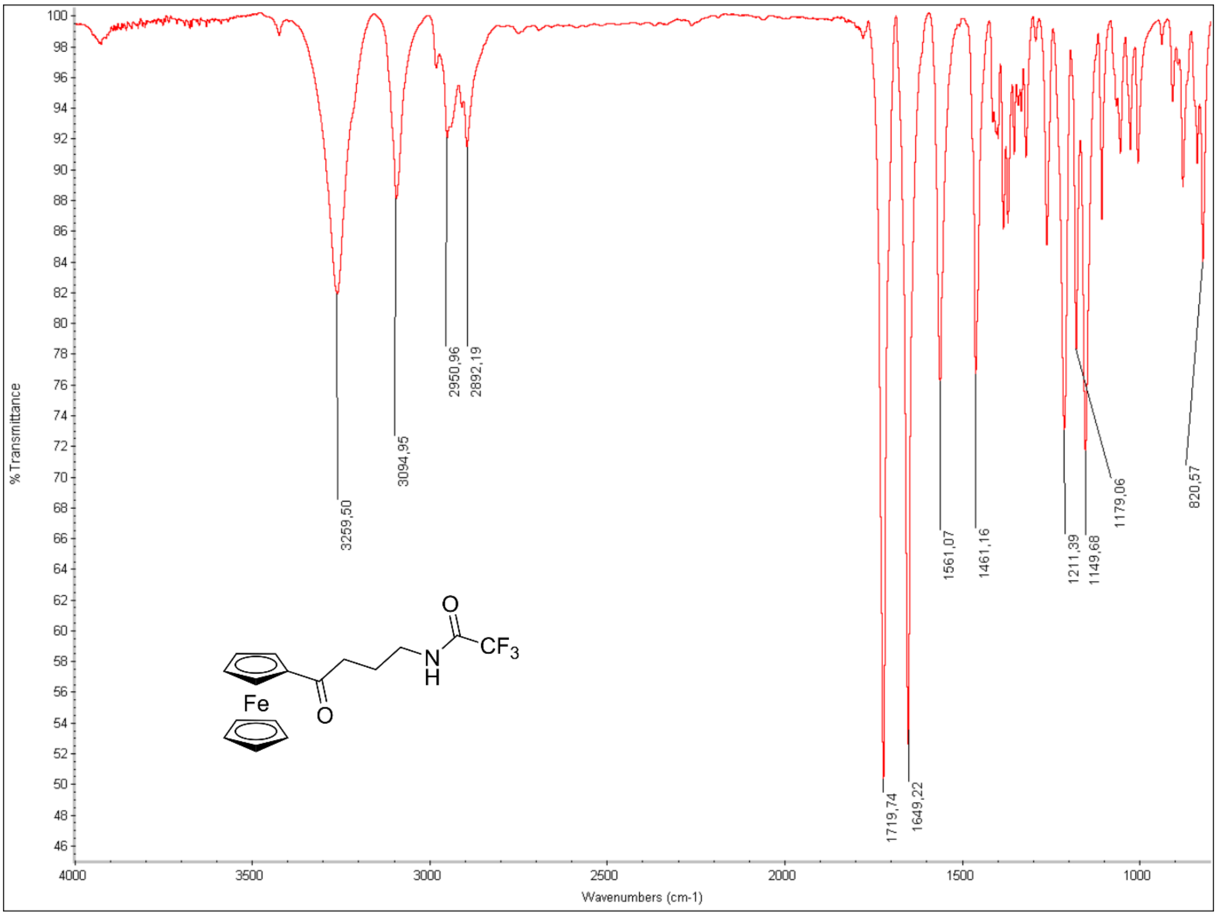

Figure S15. FT-IR spectrum of **3c**.

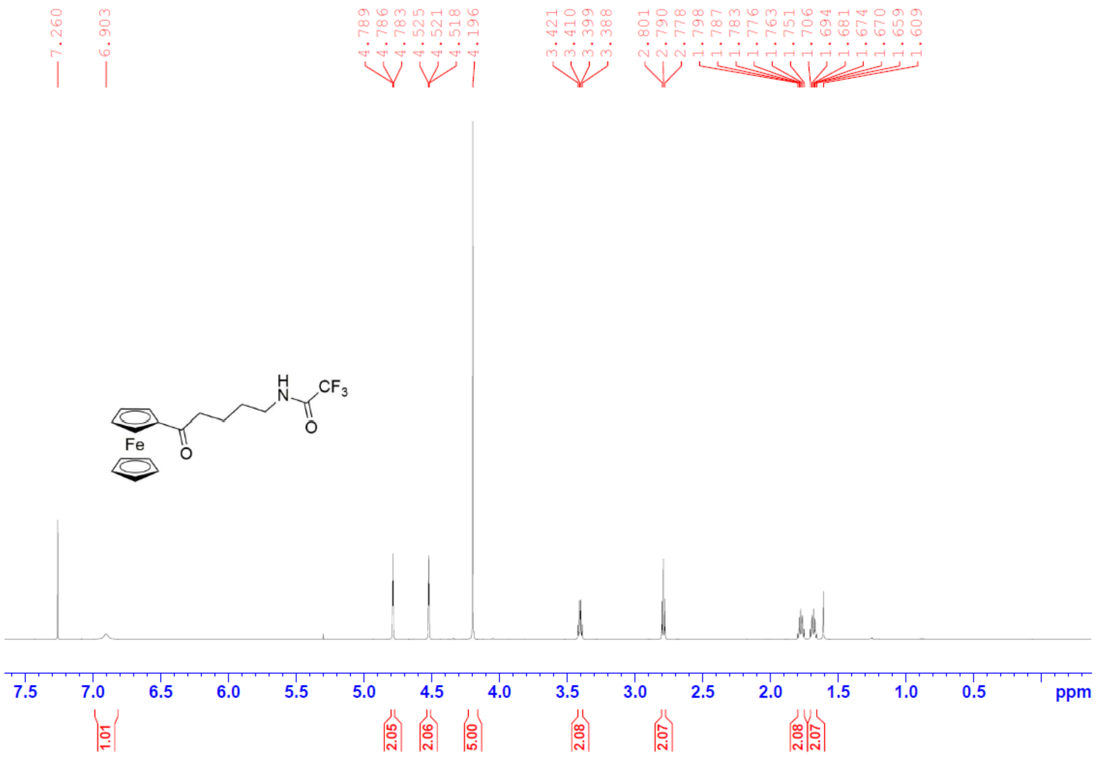

Figure S16. ^{1}H NMR spectrum of **3d** in $CDCl_3$ (600 MHz).

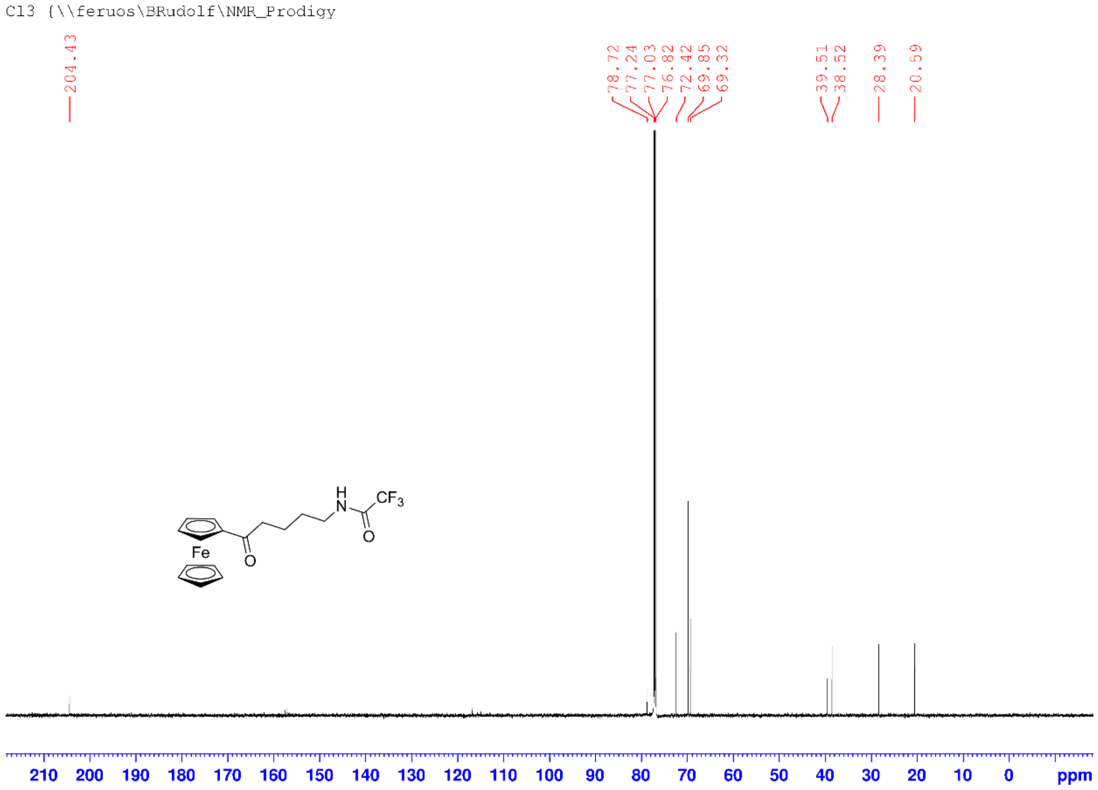

Figure S17. $^{13}C\{^1H\}$ NMR spectrum of **3d** in $CDCl_3$ (151 MHz).

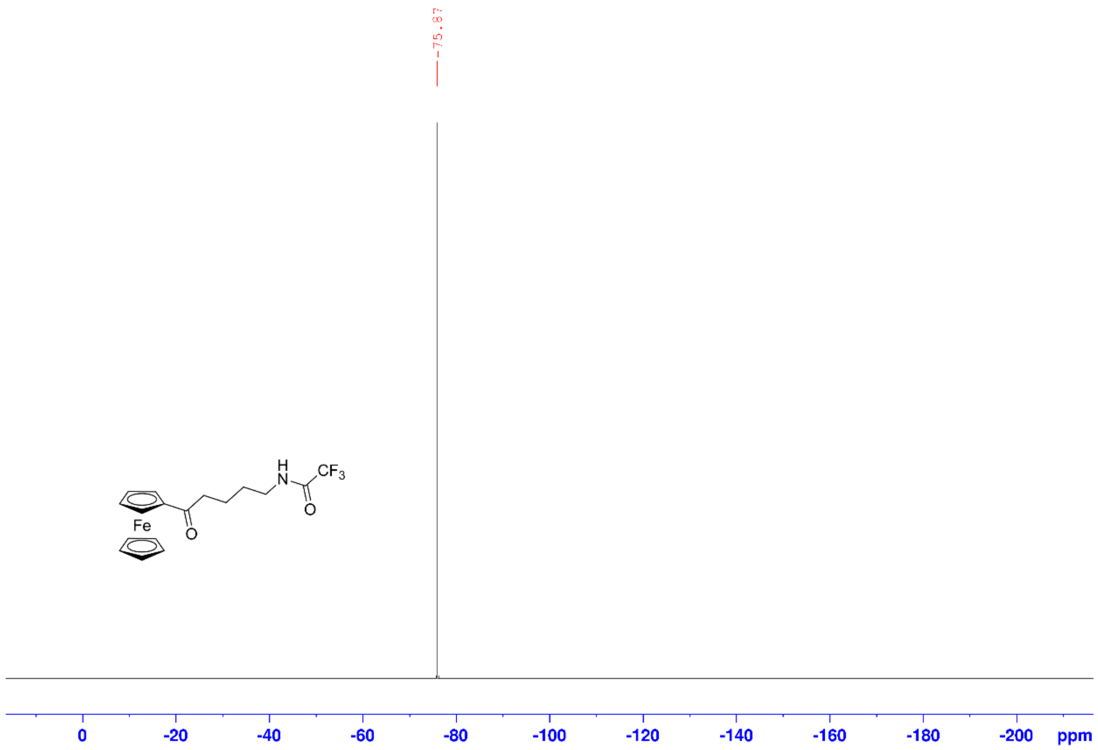

Figure S18. ^{19}F NMR spectrum of **3d** in $CDCl_3$ (565 MHz).

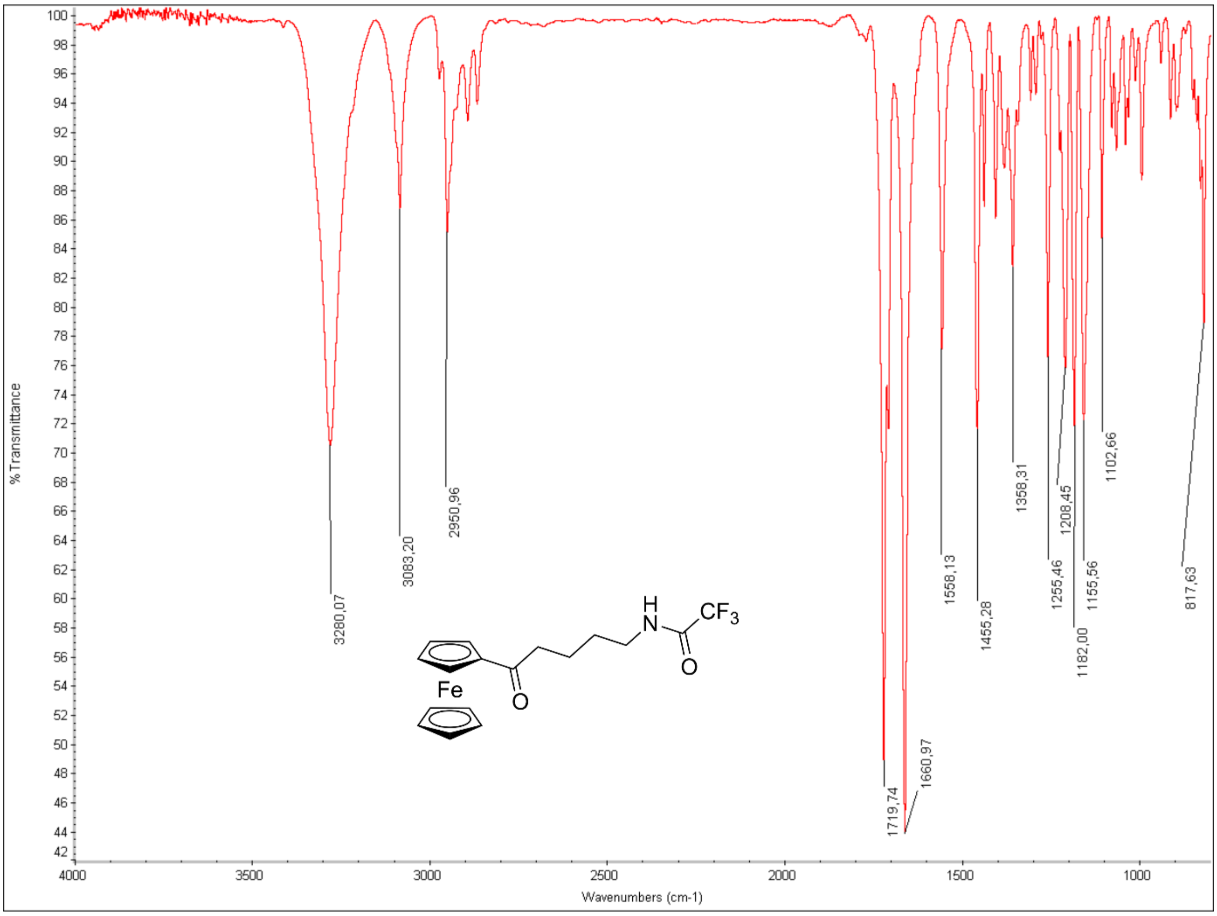

Figure S19. FT-IR spectrum of **3d**.

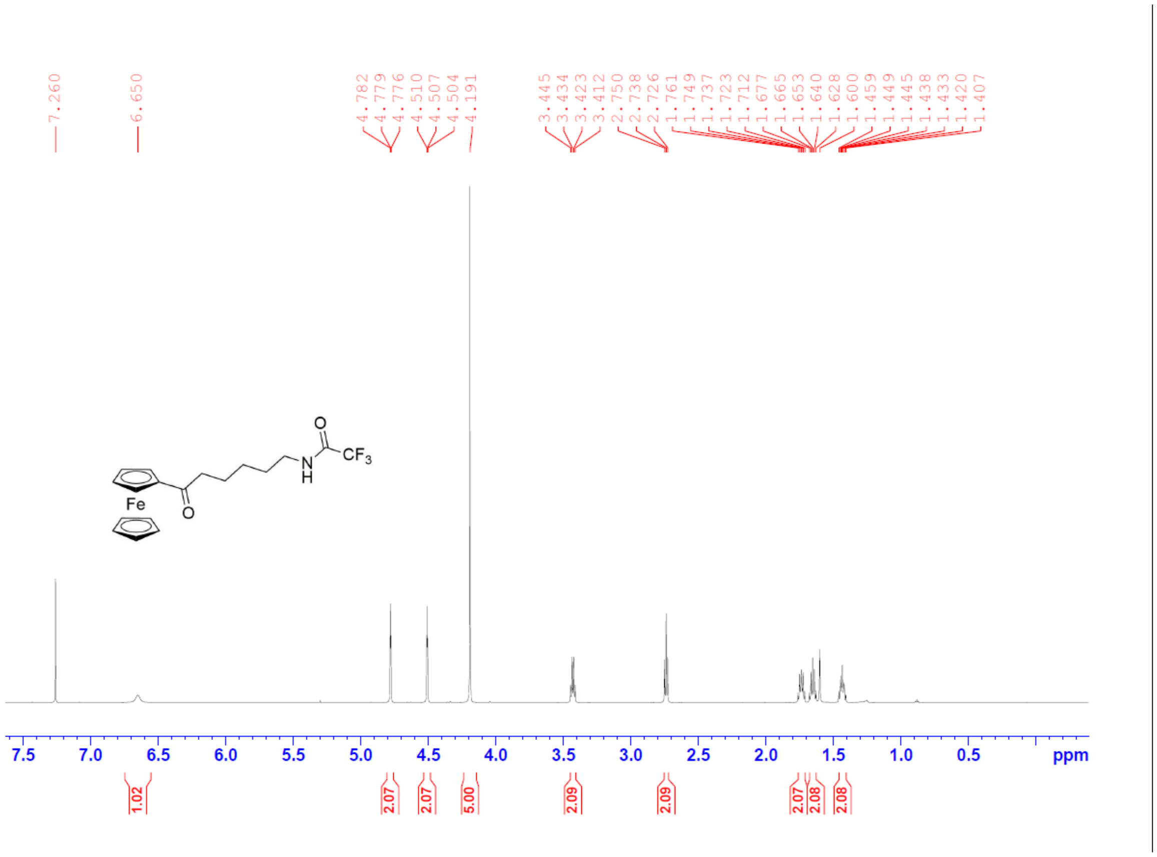

Figure S20. ^{1}H NMR spectrum of **3e** in $CDCl_3$ (600 MHz).

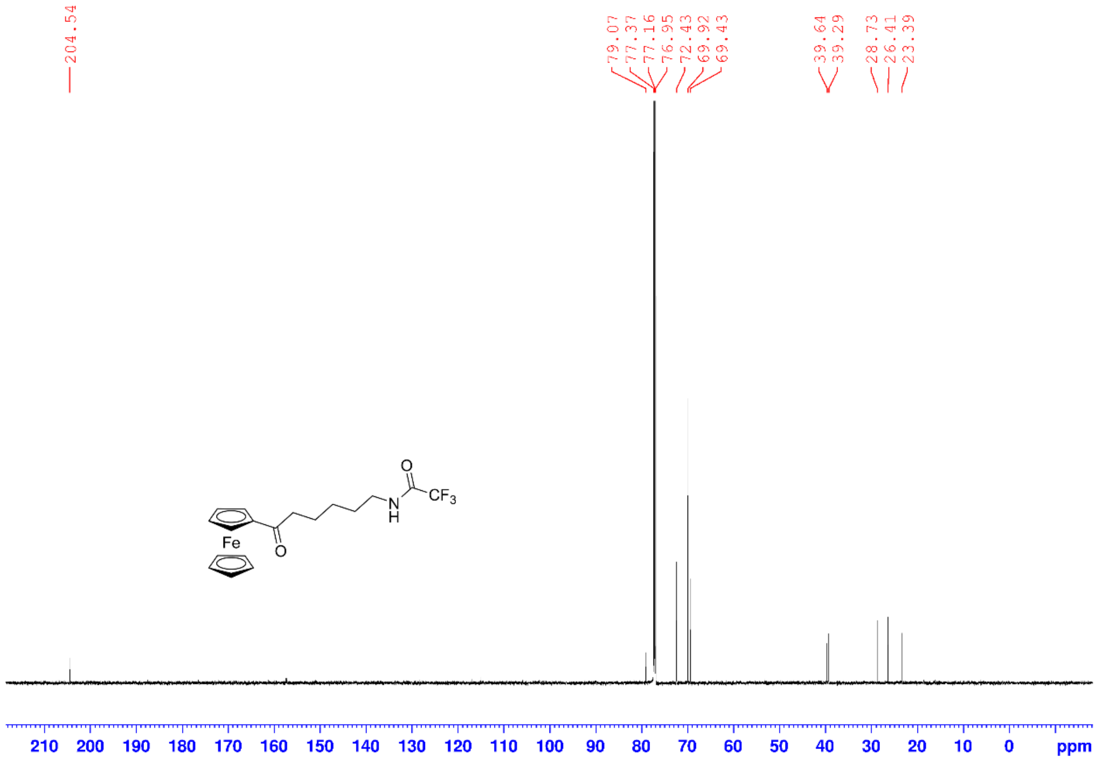

Figure S21. $^{13}C\{^1H\}$ NMR spectrum of **3e** in $CDCl_3$ (151 MHz).

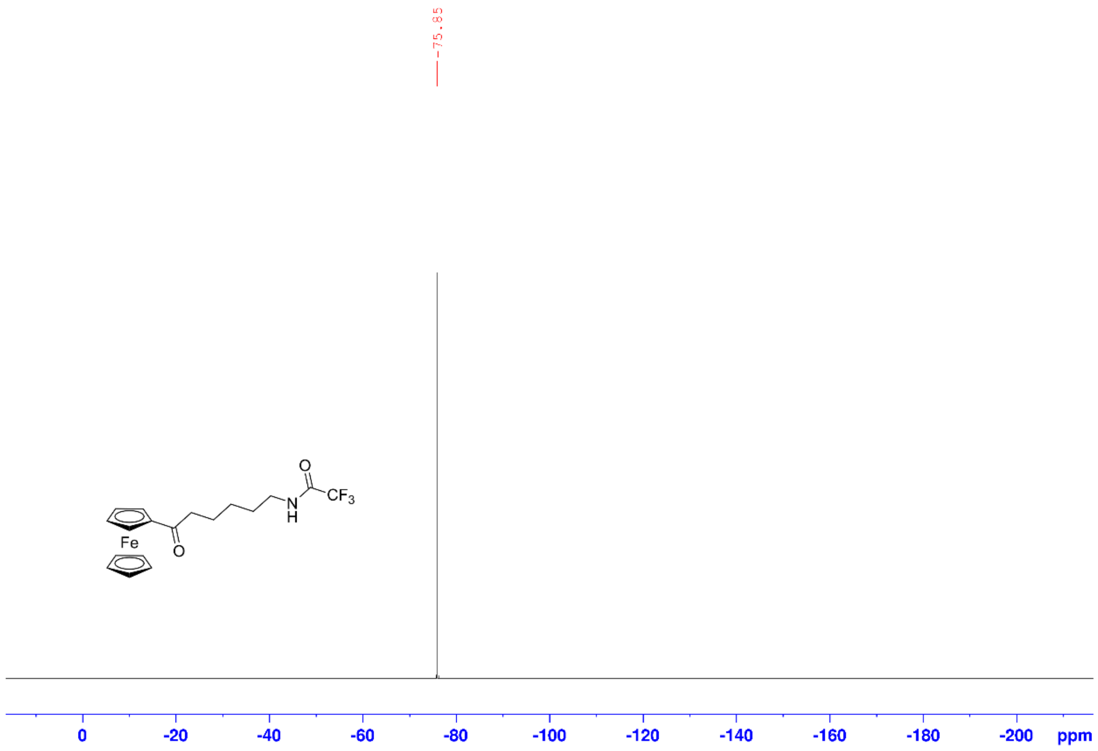

Figure S22. ^{19}F NMR spectrum of **3e** in $CDCl_3$ (565 MHz).

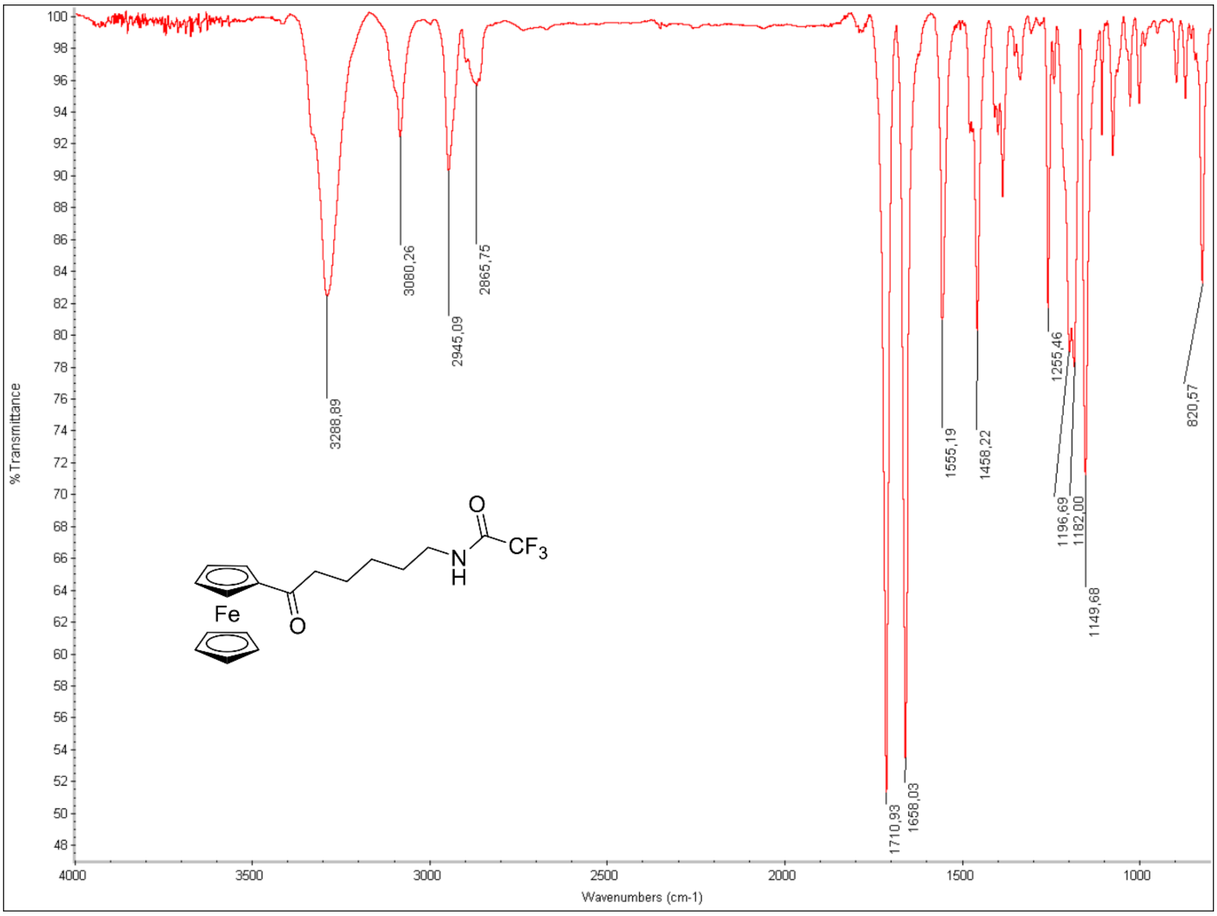

Figure S23. FT-IR spectrum of **3e**.

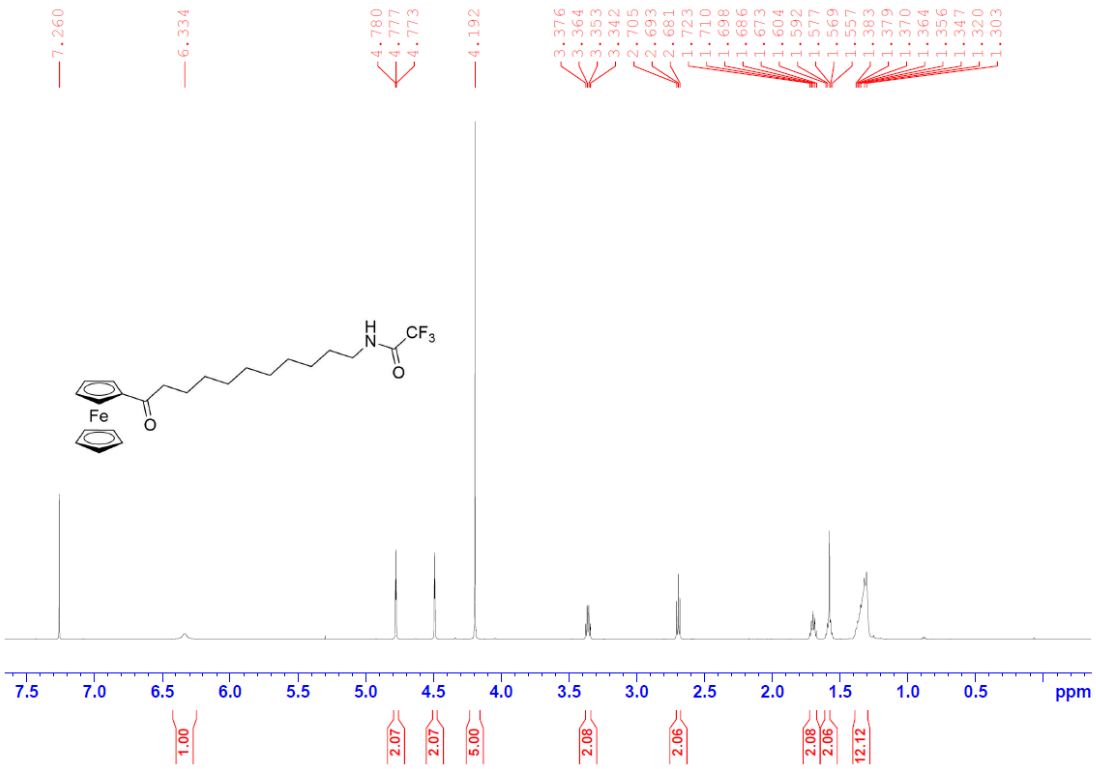

Figure S24. ^{1}H NMR spectrum of **3f** in $CDCl_3$ (600 MHz).

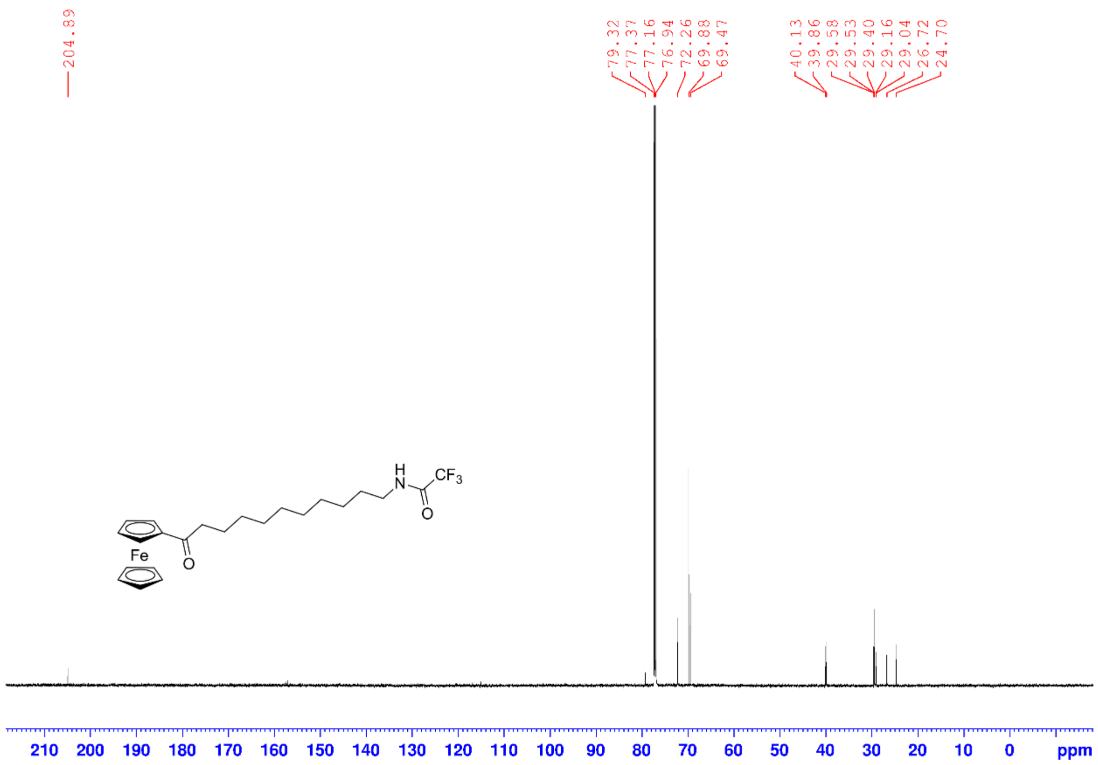

Figure S25. $^{13}C\{^{1}H\}$ NMR spectrum of **3f** in $CDCl_3$ (151 MHz).

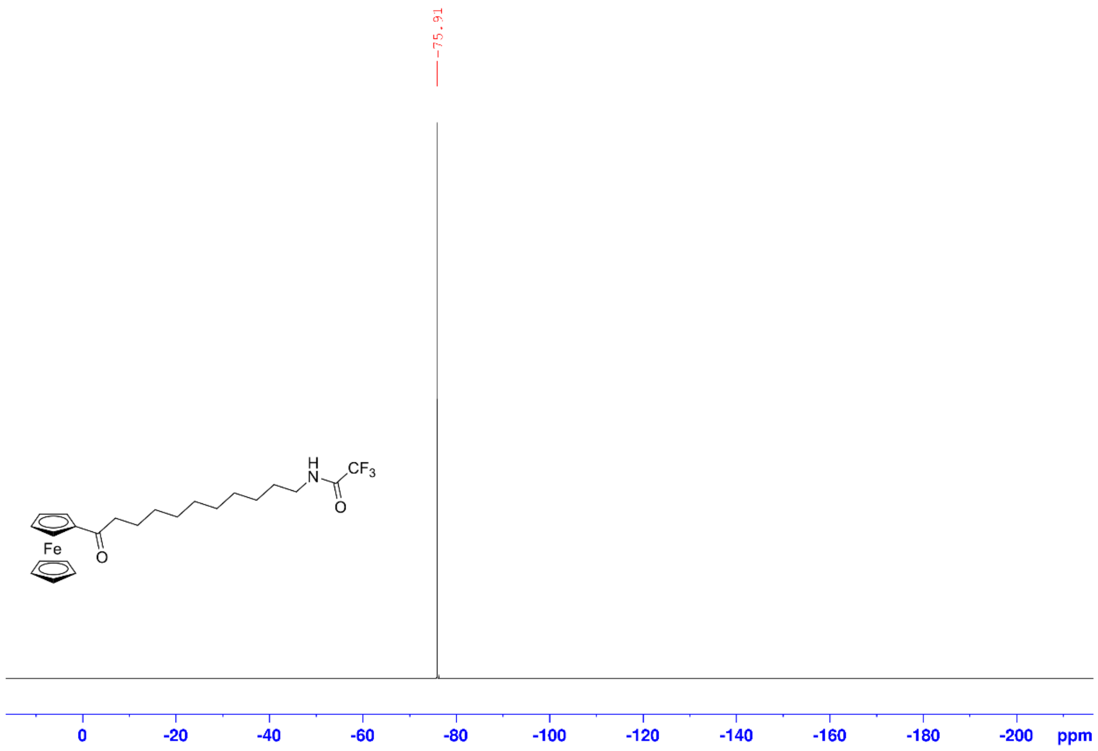

Figure S26. ^{19}F NMR spectrum of **3f** in $CDCl_3$ (565 MHz).

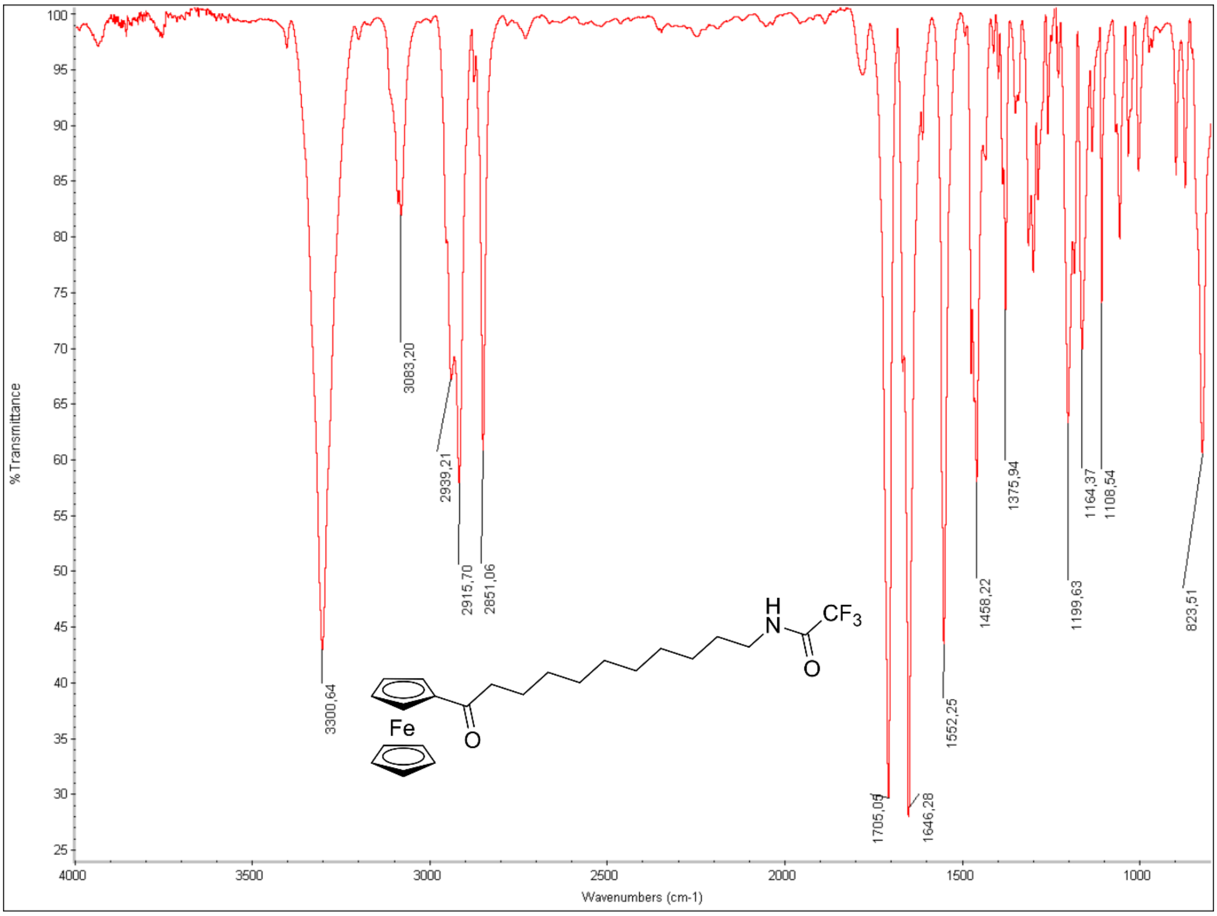

Figure S27. FT-IR spectrum of **3f**.

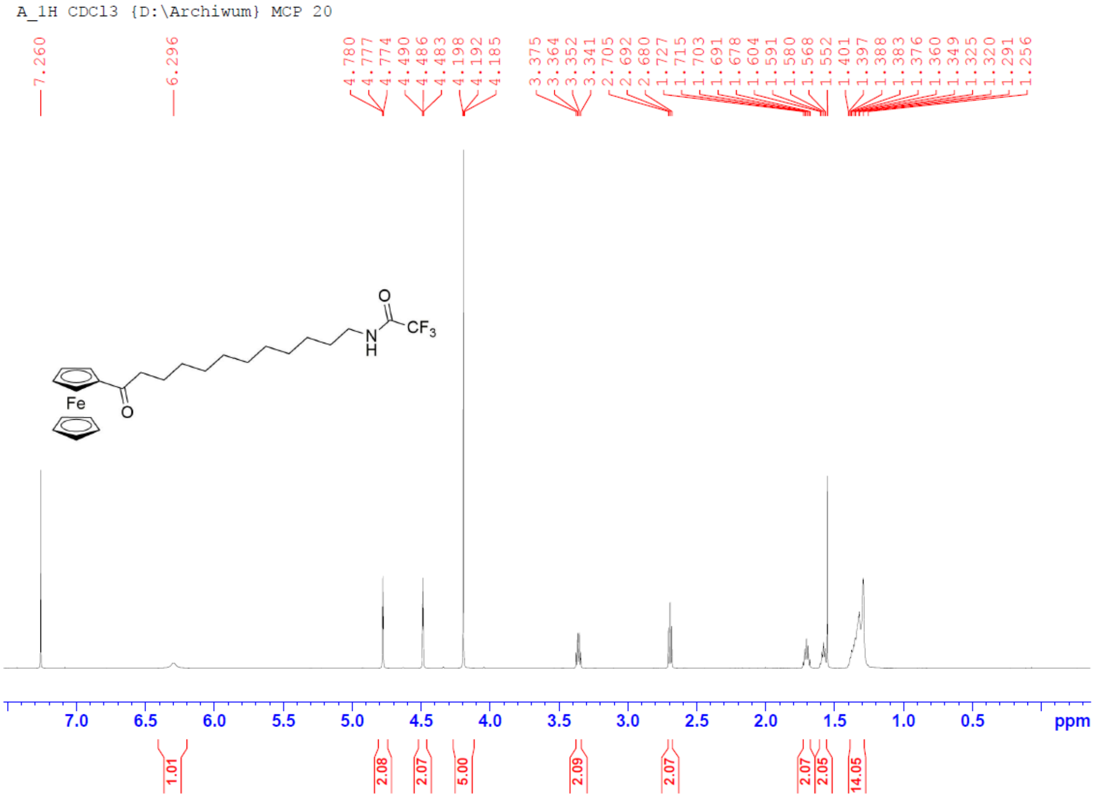

Figure S28. ^{1}H NMR spectrum of **3g** in $CDCl_3$ (600 MHz).

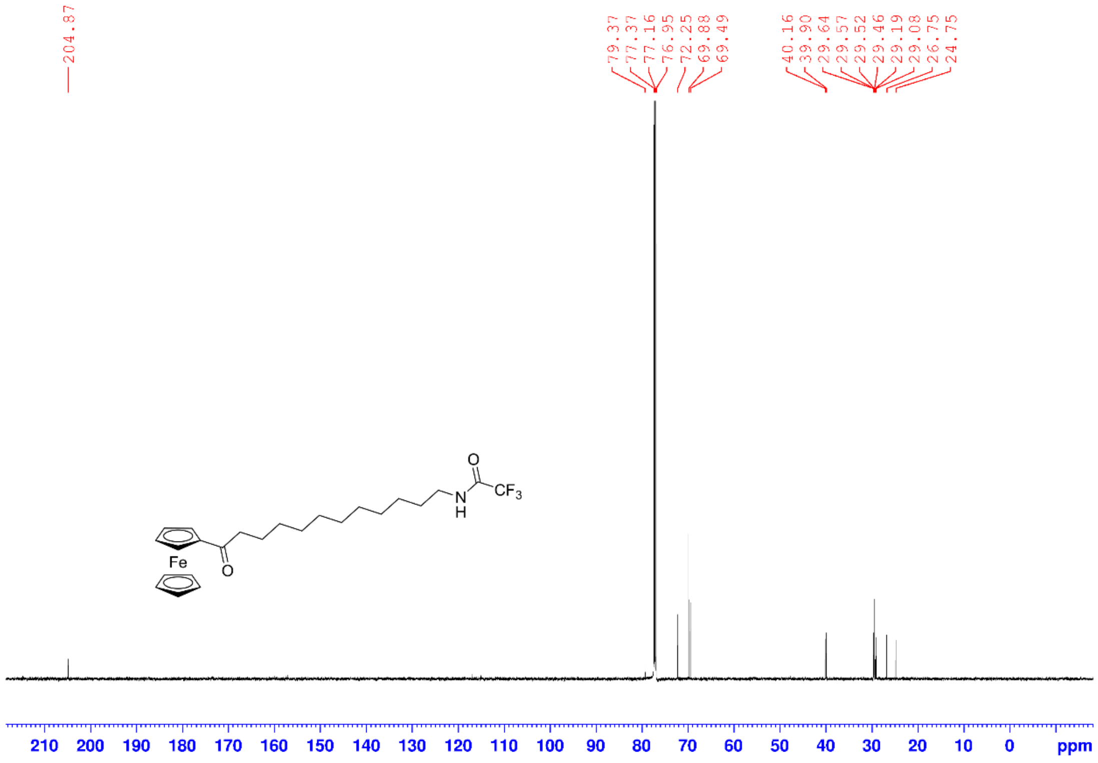

Figure S29. $^{13}C\{^{1}H\}$ NMR spectrum of **3g** in $CDCl_3$ (151 MHz).

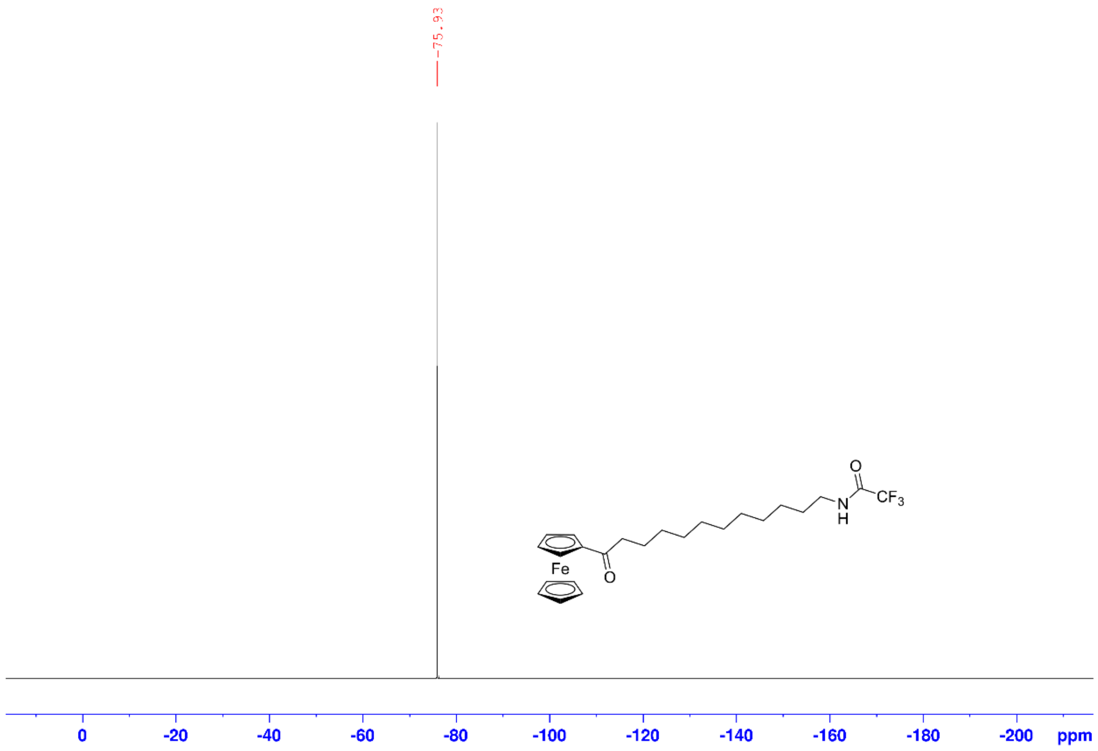

Figure S30. ^{19}F NMR spectrum of **3g** in $CDCl_3$ (565 MHz).

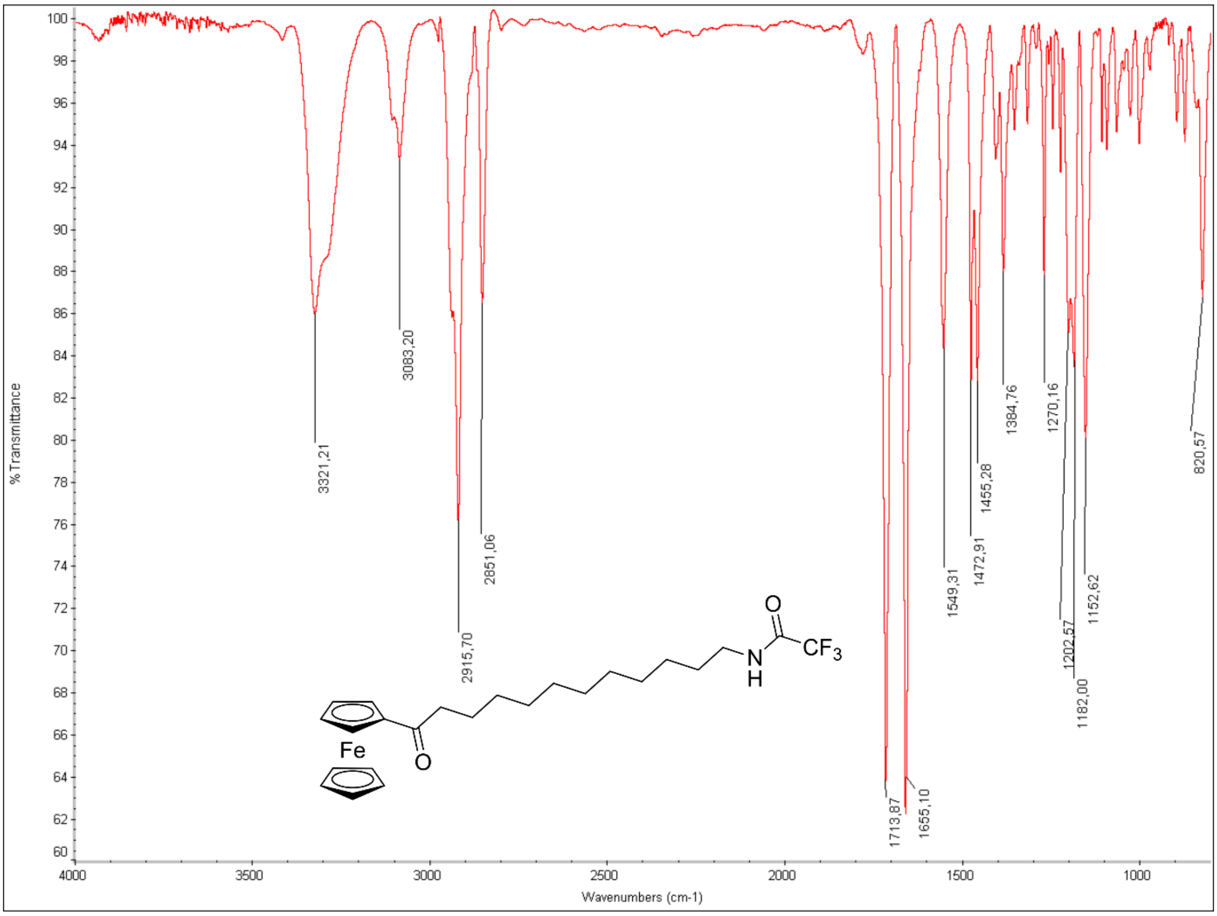

Figure S31. FT-IR spectrum of **3g**.

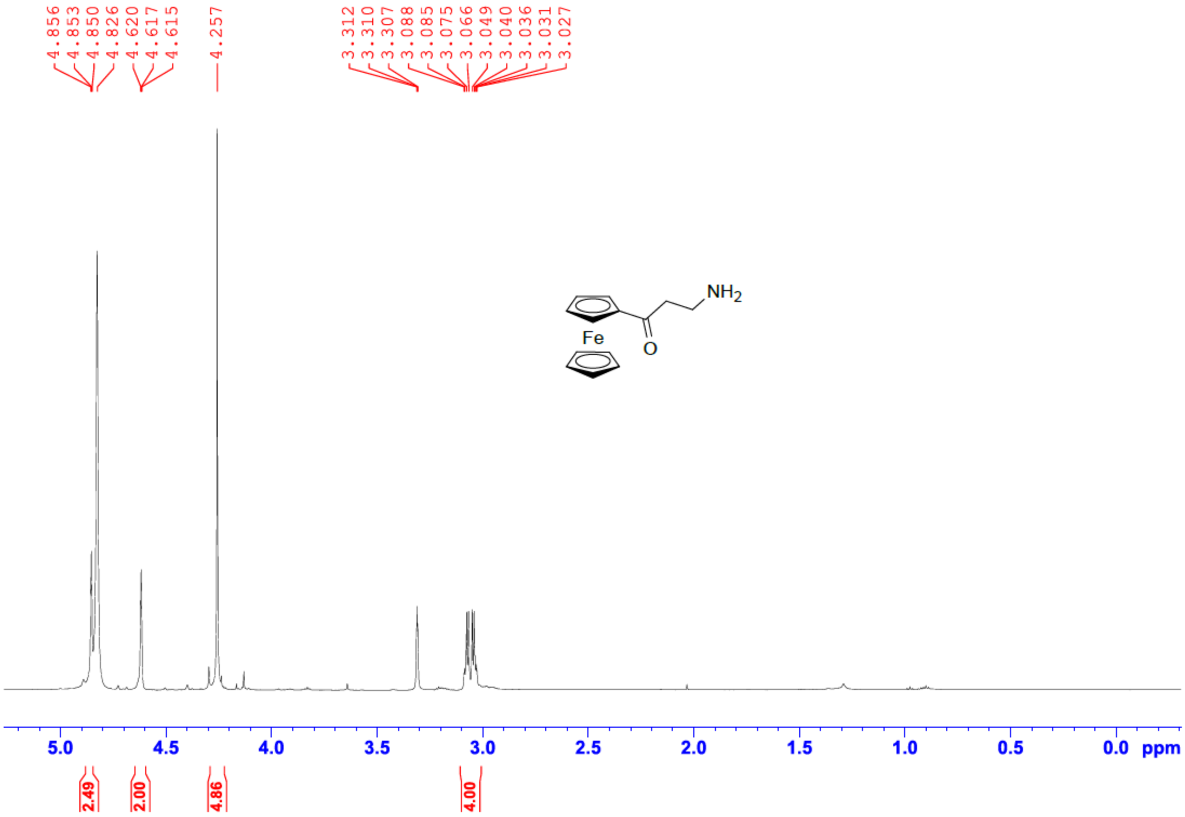

Figure S32. ^{1}H NMR spectrum of **10** in CD_3OD (600 MHz).

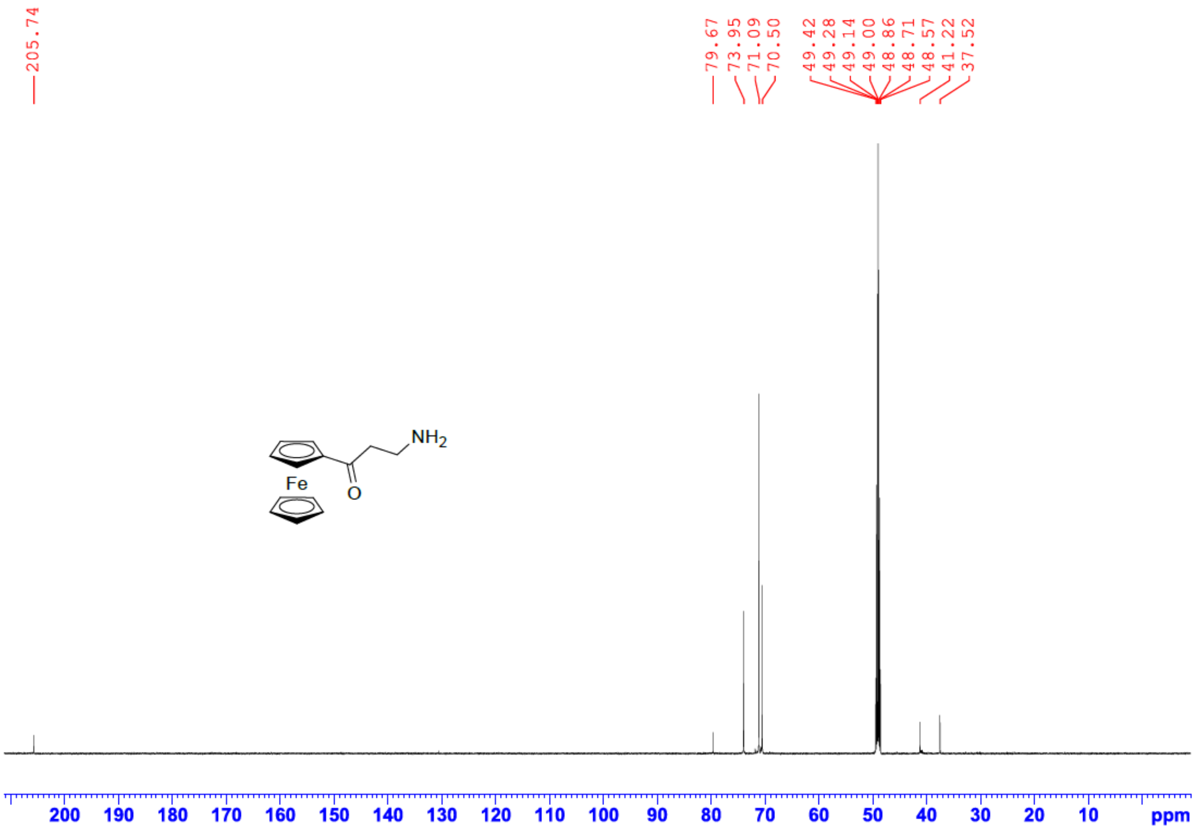

Figure S33. $^{13}C\{^{1}H\}$ NMR spectrum of **10** in CD_3OD (600 MHz).

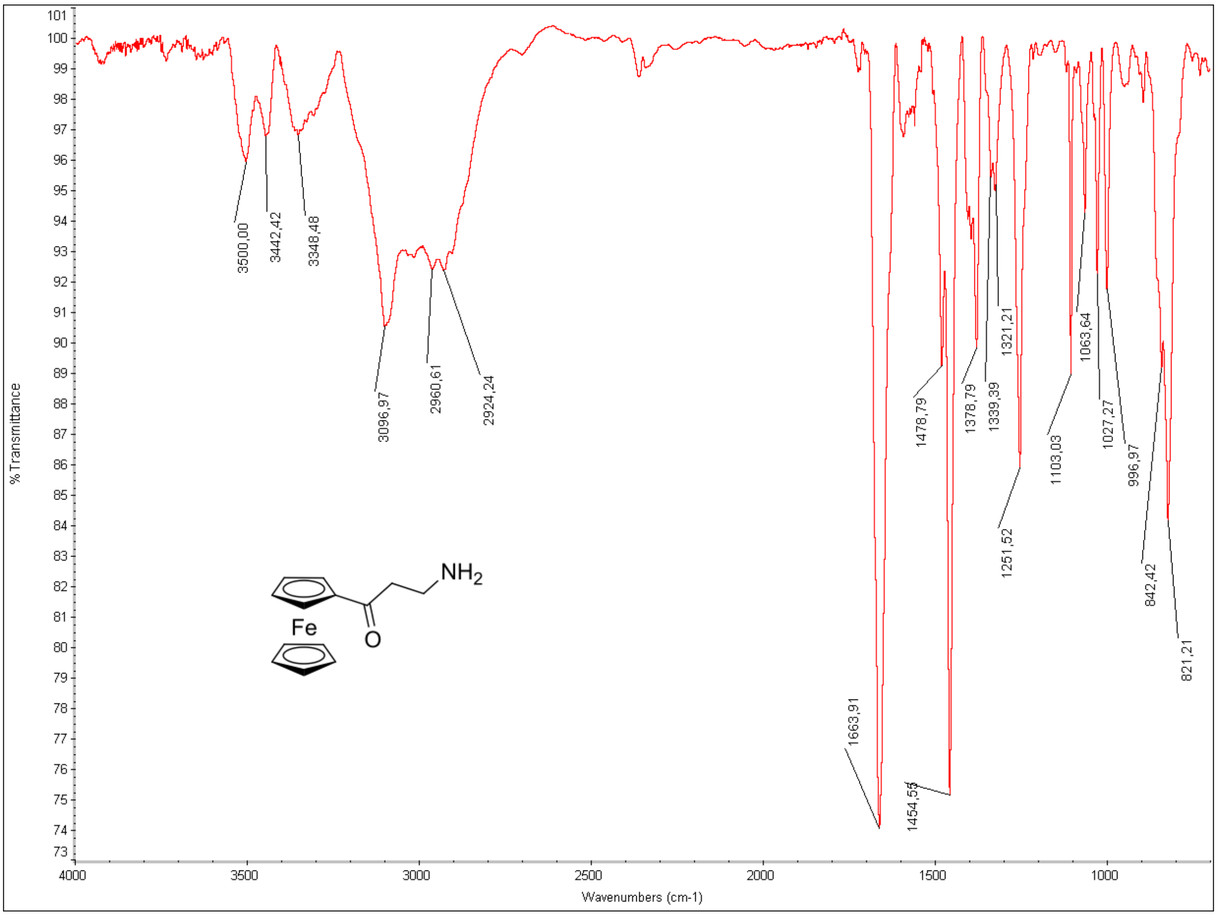

Figure S34. FT-IR spectrum of **10**.

Single Mass Analysis
Tolerance = 5.0 PPM / DBE: min = -1.5, max = 60.0
Element prediction: Off
Number of isotope peaks used for i-FIT = 9

Monoisotopic Mass, Even Electron Ions
155 formula(e) evaluated with 1 results within limits (all results (up to 1000) for each mass)
Elements Used:
C: 0-70 H: 0-60 N: 0-4 O: 0-4 Fe: 0-1

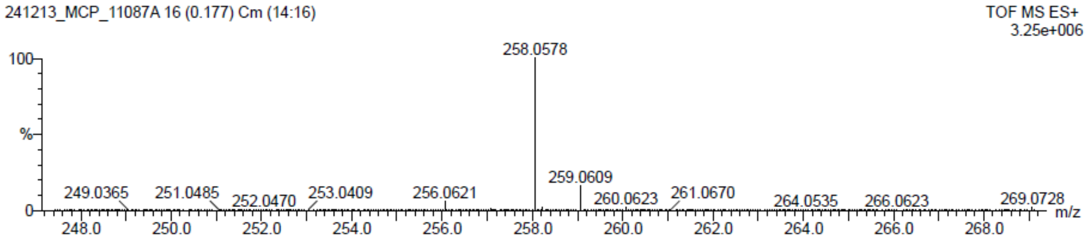

Minimum:				-1.5				
Maximum:		5.0	5.0	60.0				
Mass	Calc. Mass	mDa	PPM	DBE	i-FIT	Norm	Conf(%)	Formula
258.0578	258.0581	-0.3	-1.2	6.5	1834.0	n/a	n/a	C13 H16 N O Fe

Figure S35. HRMS spectrum of **10**.

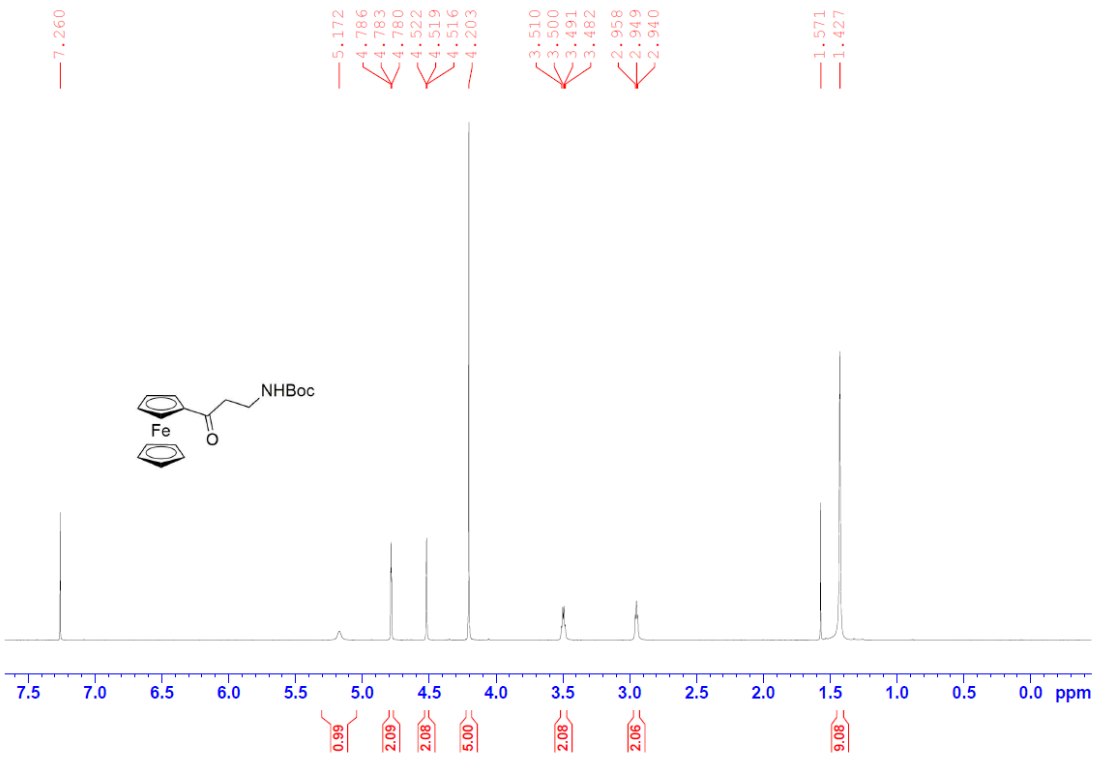

Figure S36. ^{1}H NMR spectrum of **11** in $CDCl_3$ (600 MHz).

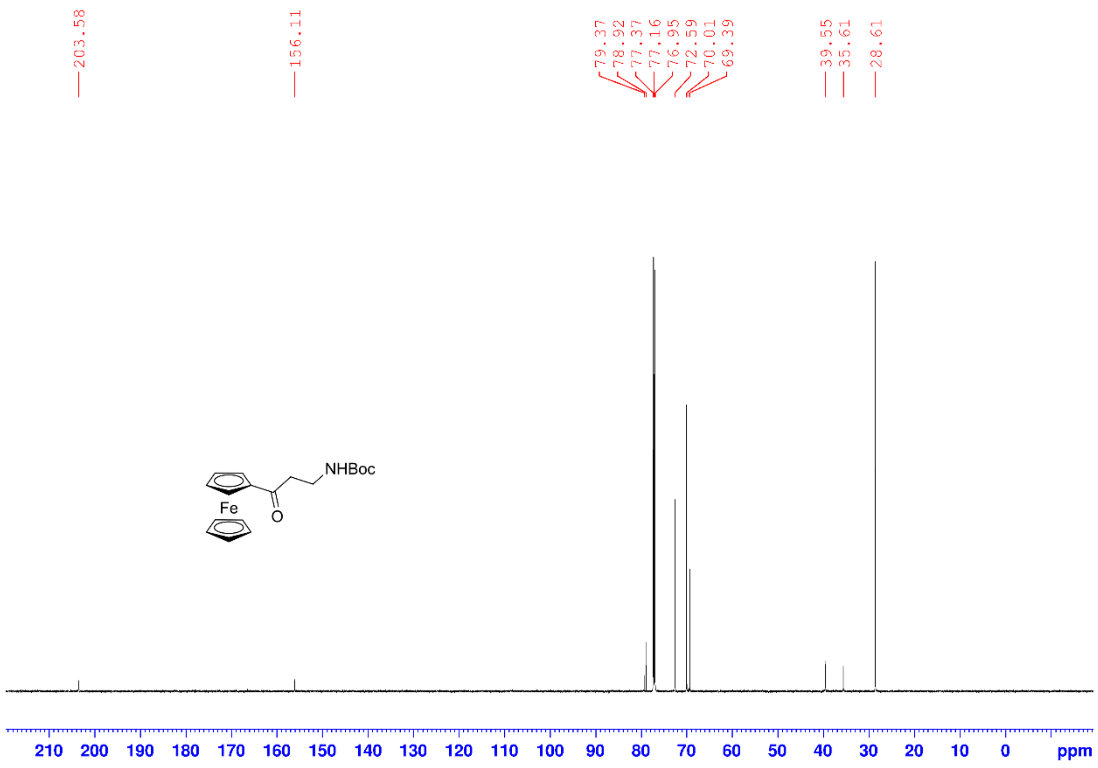

Figure S37. $^{13}C\{^{1}H\}$ NMR spectrum of **11** in $CDCl_3$ (151 MHz).

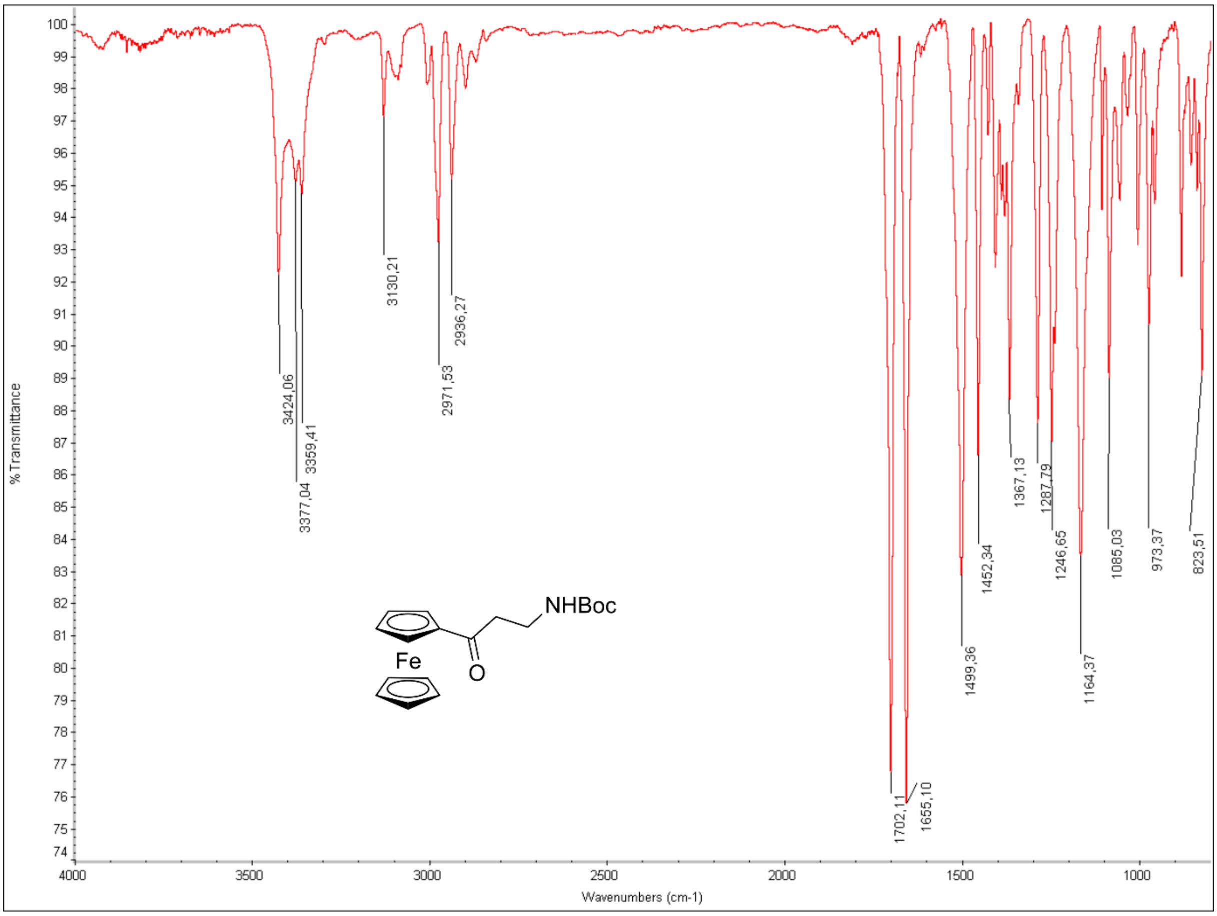

Figure S38. FT-IR spectrum of **11**.

NMR and HRMS spectra of 8a – g and 9.

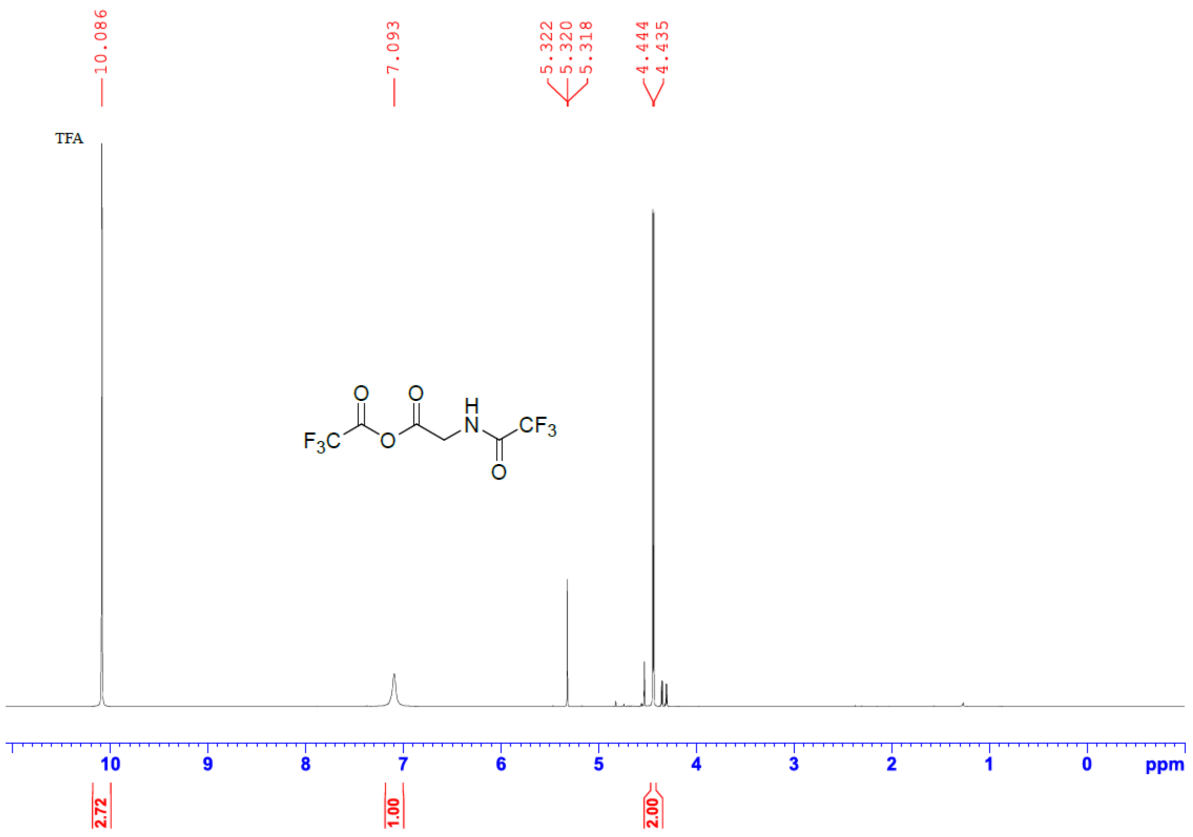

Figure S39. ^{1}H NMR spectrum of **8a** in CD_2Cl_2 (600 MHz).

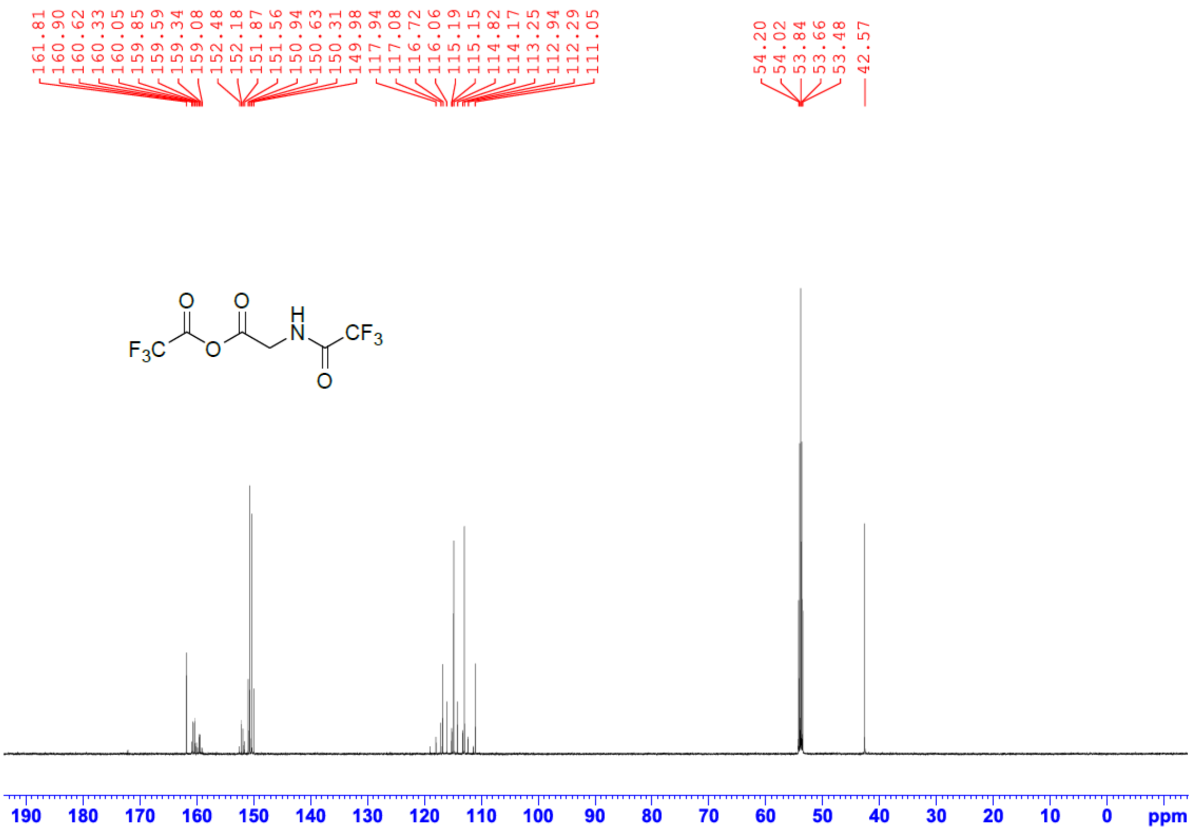

Figure S40. $^{13}C\{^1H\}$ NMR spectrum of **8a** in CD_2Cl_2 (151 MHz).

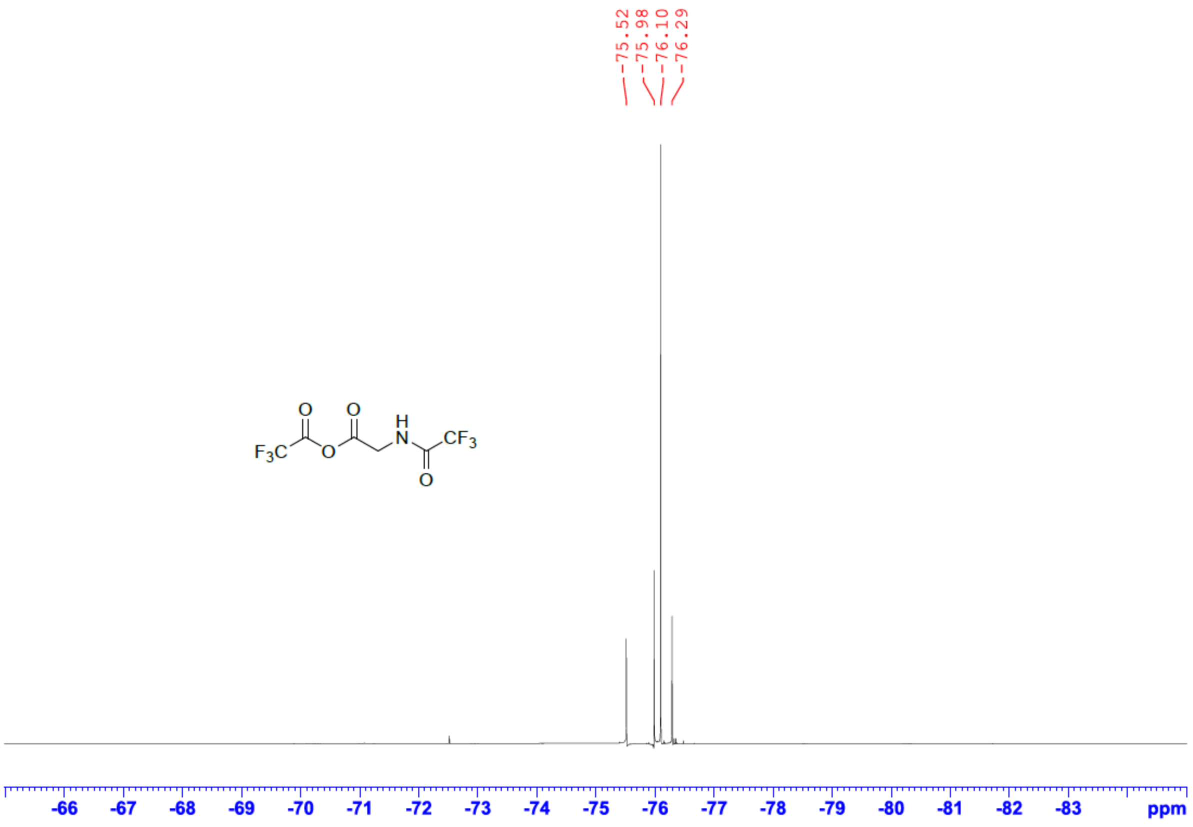

Figure S41. ^{19}F NMR spectrum of **8a** in CD_2Cl_2 (565 MHz).

Elemental Composition Report **Page 1**

Single Mass Analysis
Tolerance = 5.0 PPM / DBE: min = -1.5, max = 60.0
Element prediction: Off
Number of isotope peaks used for i-FIT = 9

Monoisotopic Mass, Even Electron Ions
201 formula(e) evaluated with 4 results within limits (all results (up to 1000) for each mass)
Elements Used:
C: 0-20 H: 0-30 N: 0-1 O: 0-5 F: 0-6

250110_MCP_11062aA 25 (0.277) Cm (25:31-38:43) TOF MS ES- 4.88e+005

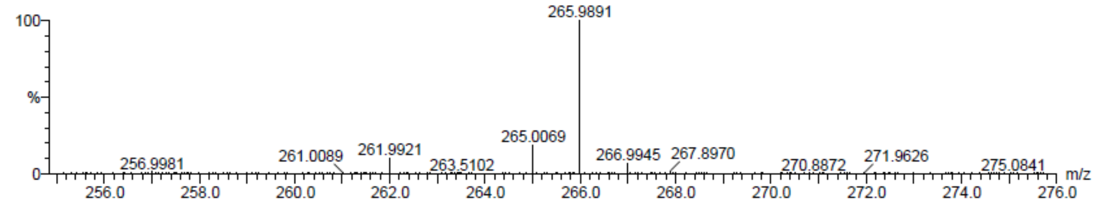

Minimum:				-1.5				
Maximum:		5.0	5.0	60.0				
Mass	Calc. Mass	mDa	PPM	DBE	i-FIT	Norm	Conf(%)	Formula
265.9891	265.9888	0.3	1.1	3.5	570.3	0.002	99.75	C6 H2 N O4 F6
	265.9901	-1.0	-3.8	10.5	576.8	6.508	0.15	C11 H2 N O5 F2
	265.9890	0.1	0.4	14.5	577.6	7.325	0.07	C14 H N O4 F
	265.9878	1.3	4.9	18.5	578.3	7.975	0.03	C17 N O3

Figure S42. HRMS spectrum of **8a**.

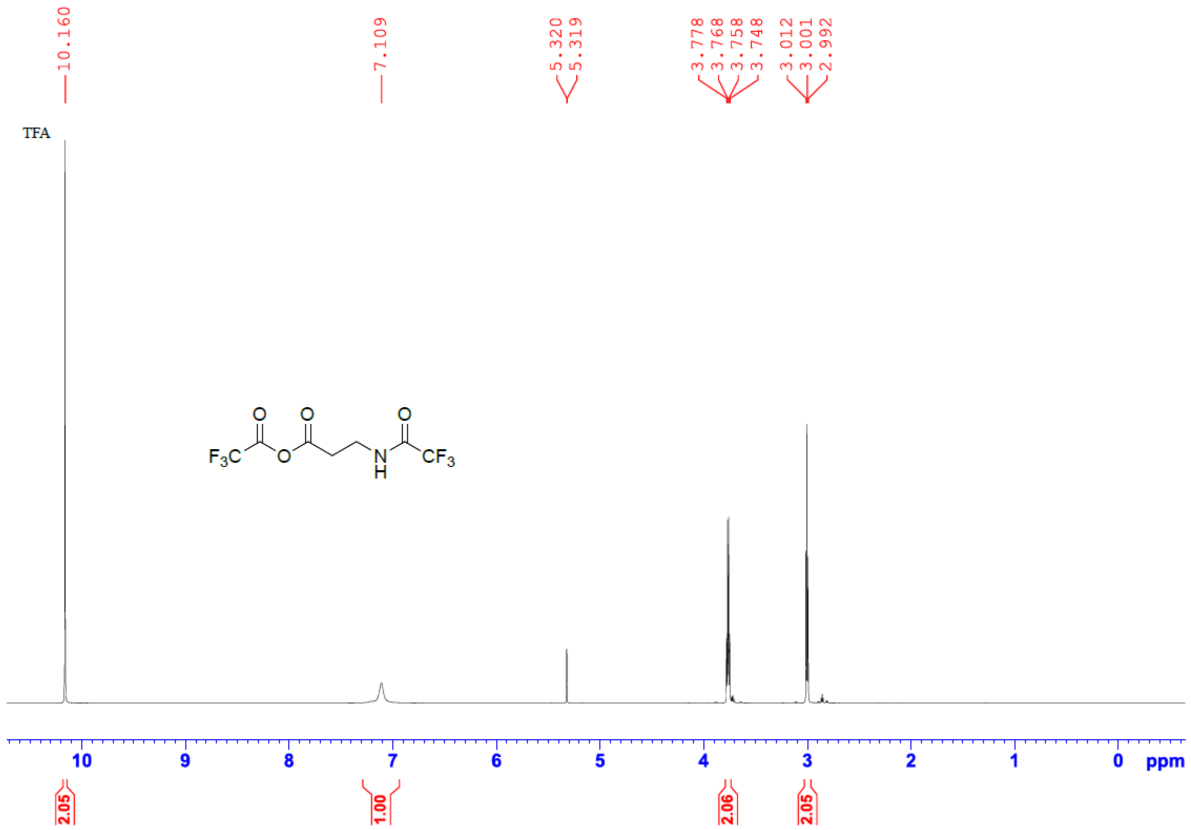

Figure S43. ^{1}H NMR spectrum of **8b** in CD_2Cl_2 (600 MHz).

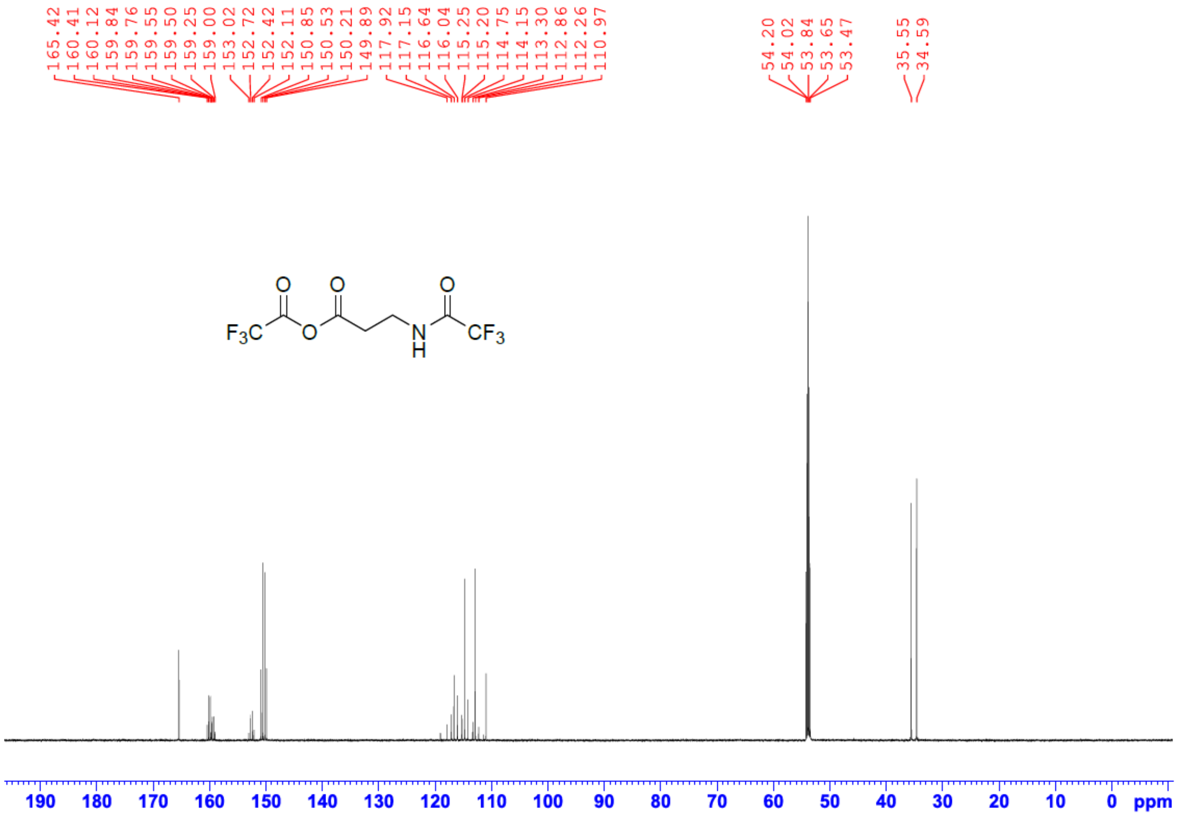

Figure S44. $^{13}C\{^1H\}$ NMR spectrum of **8b** in CD_2Cl_2 (151 MHz).

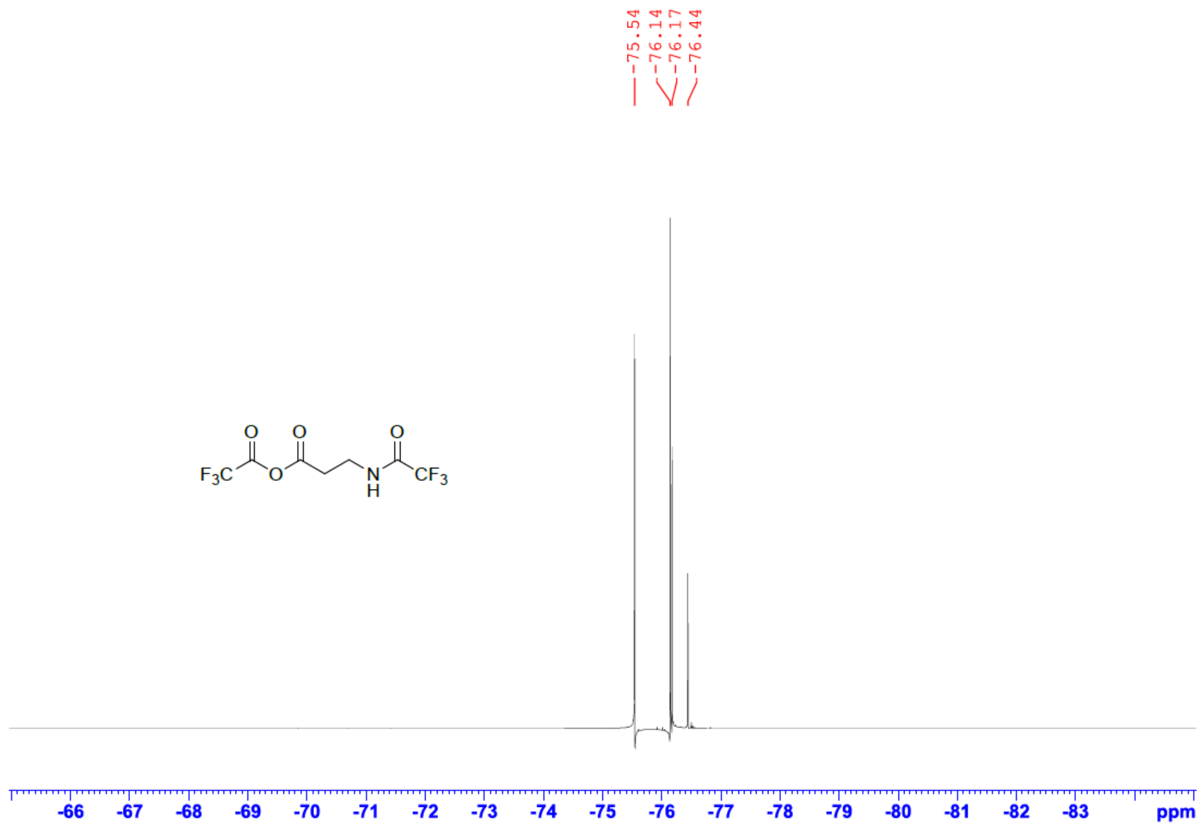

Figure S45. ^{19}F NMR spectrum of **8b** in CD_2Cl_2 (565 MHz).

Elemental Composition Report **Page 1**

Single Mass Analysis
Tolerance = 5.0 PPM / DBE: min = -1.5, max = 60.0
Element prediction: Off
Number of isotope peaks used for i-FIT = 9

Monoisotopic Mass, Even Electron Ions
215 formula(e) evaluated with 4 results within limits (all results (up to 1000) for each mass)
Elements Used:
C: 0-40 H: 0-30 N: 0-2 O: 0-3 F: 0-6
250114_MCP_11062b_new_drugaA 29 (0.311) Cm (29:36) TOF MS ES-
8.75e+006

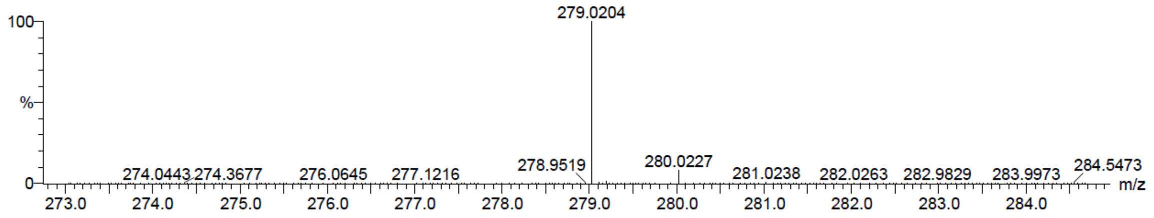

		mDa	PPM	DBE				
Minimum:				-1.5				
Maximum:		5.0	5.0	60.0				

Mass	Calc. Mass	mDa	PPM	DBE	i-FIT	Norm	Conf(%)	Formula
279.0204	279.0204	0.0	0.0	3.5	1063.5	0.000	100.00	C7 H5 N2 O3 F6
	279.0193	1.1	3.9	7.5	1078.6	15.105	0.00	C10 H4 N2 O2 F5
	279.0206	-0.2	-0.7	14.5	1087.3	23.837	0.00	C15 H4 N2 O3 F
	279.0195	0.9	3.2	18.5	1089.6	26.127	0.00	C18 H3 N2 O2

Figure S46. HRMS spectrum of **8b**.

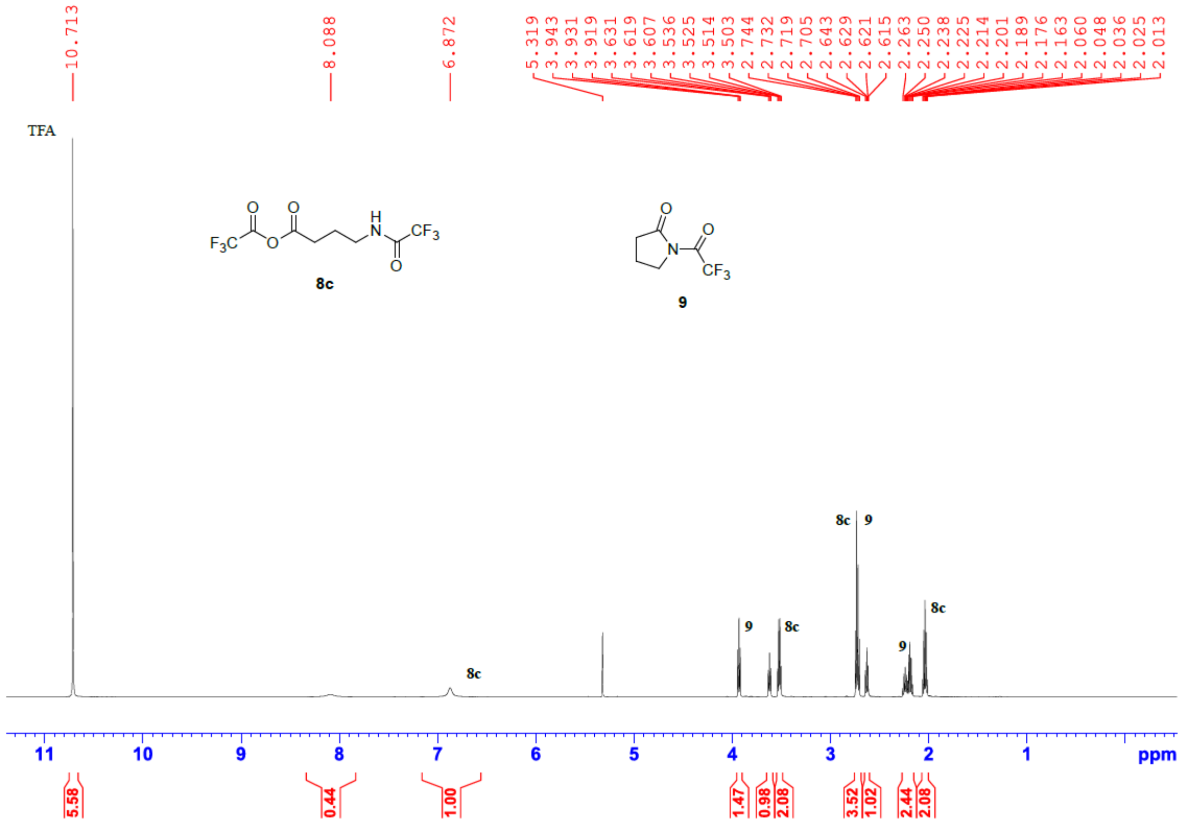

Figure S47. ^{1}H NMR spectrum of **8c** and **9** (mixture) in CD_2Cl_2 (600 MHz).

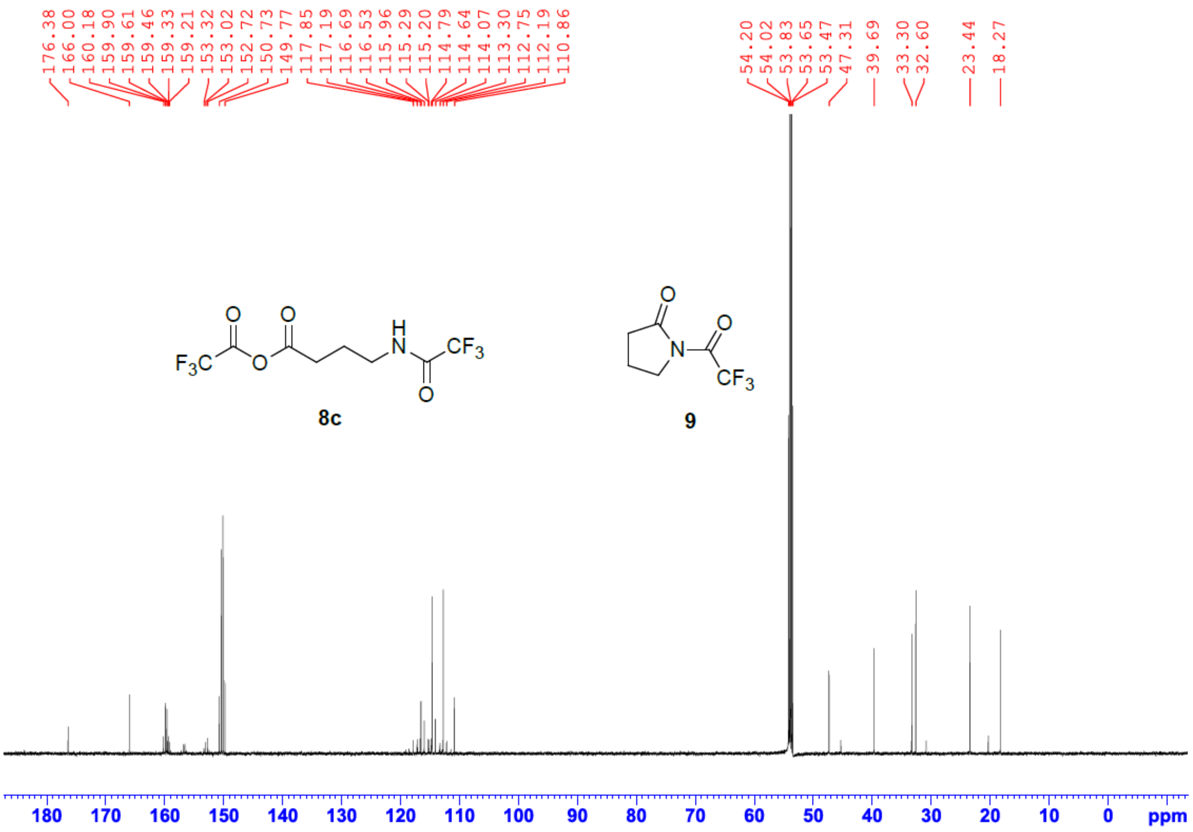

Figure S48. $^{13}C\{^{1}H\}$ NMR spectrum of **8c** and **9** (mixture) in CD_2Cl_2 (151 MHz).

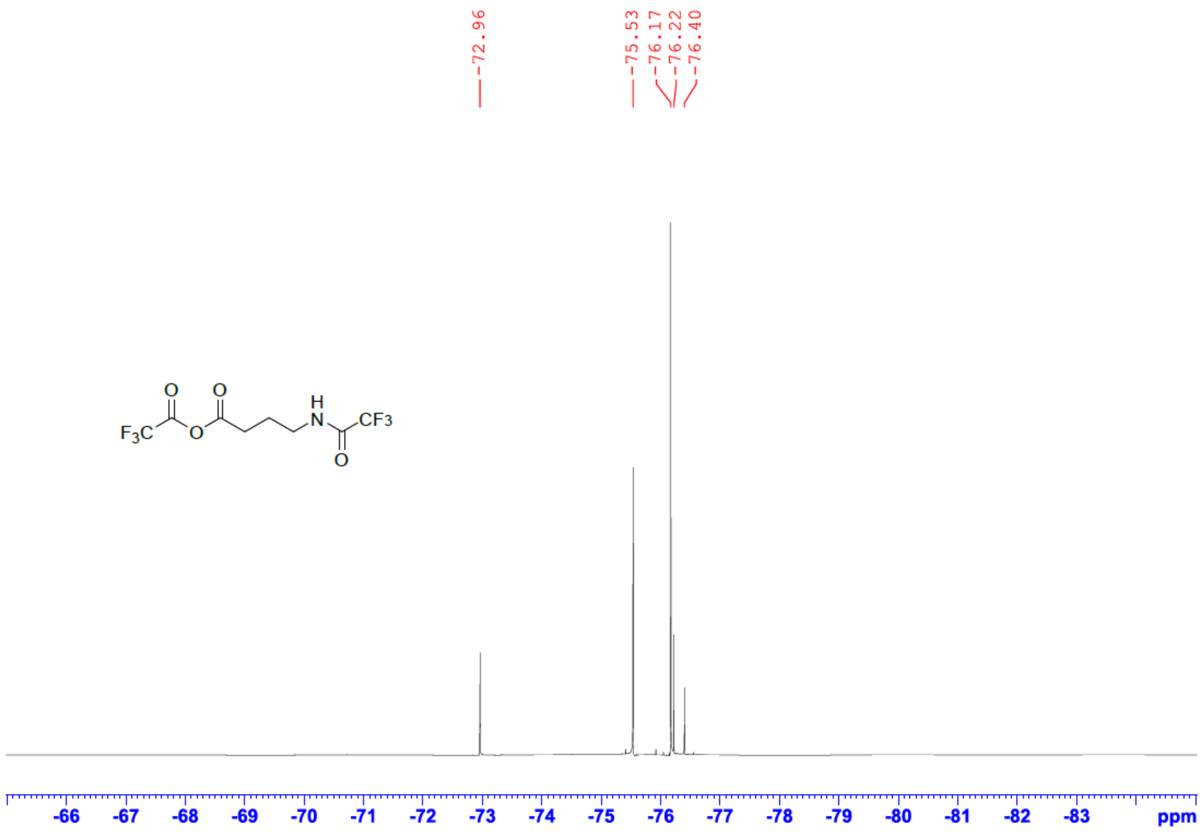

Figure S49. ^{19}F NMR spectrum of **8c** and **9** (mixture) in CD_2Cl_2 (565 MHz).

Elemental Composition Report **Page 1**

Single Mass Analysis
Tolerance = 5.0 PPM / DBE: min = -1.5, max = 60.0
Element prediction: Off
Number of isotope peaks used for i-FIT = 9

Monoisotopic Mass, Even Electron Ions
129 formula(e) evaluated with 3 results within limits (all results (up to 1000) for each mass)
Elements Used:
C: 0-15 H: 0-25 N: 0-1 O: 0-5 F: 0-6

250110_MCP_11062cA 24 (0.257) Cm (22:24-(7:9+67:70)) TOF MS ES- 7.72e+004

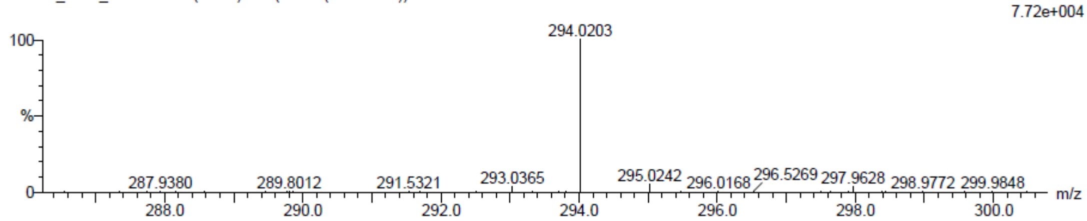

Mass	Calc. Mass	mDa	PPM	DBE	i-FIT	Norm	Conf(%)	Formula
Minimum:				-1.5				
Maximum:		5.0	5.0	60.0				
294.0203	294.0201	0.2	0.7	3.5	131.9	0.203	81.62	C8 H6 N O4 F6
	294.0190	1.3	4.4	7.5	133.5	1.755	17.29	C11 H5 N O3 F5
	294.0214	-1.1	-3.7	10.5	136.3	4.520	1.09	C13 H6 N O5 F2

Figure S50. HRMS spectrum of **8c**.

Elemental Composition Report

Page 1

Single Mass Analysis

Tolerance = 5.0 PPM / DBE: min = -1.5, max = 60.0
Element prediction: Off
Number of isotope peaks used for i-FIT = 9

Monoisotopic Mass, Even Electron Ions
284 formula(e) evaluated with 2 results within limits (all results (up to 1000) for each mass)
Elements Used:
C: 0-20 H: 0-30 N: 0-3 O: 0-6 F: 0-6

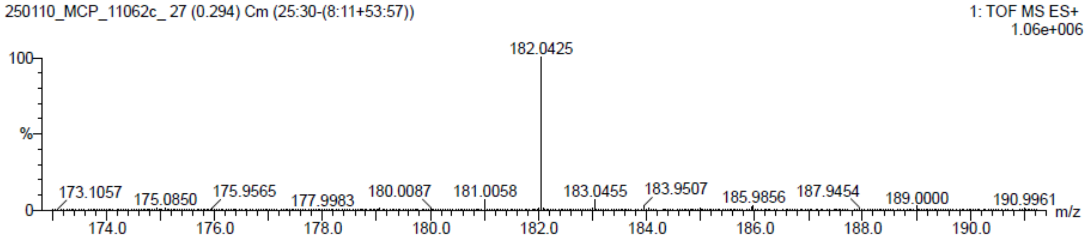

Minimum:				-1.5				
Maximum:		5.0	5.0	60.0				
Mass	Calc. Mass	mDa	PPM	DBE	i-FIT	Norm	Conf(%)	Formula
182.0425	182.0429	-0.4	-2.2	2.5	2082.5	0.006	99.39	C6 H7 N O2 F3
	182.0417	0.8	4.4	6.5	2087.5	5.092	0.61	C9 H6 N O F2

Figure S51. HRMS spectrum of **9**.

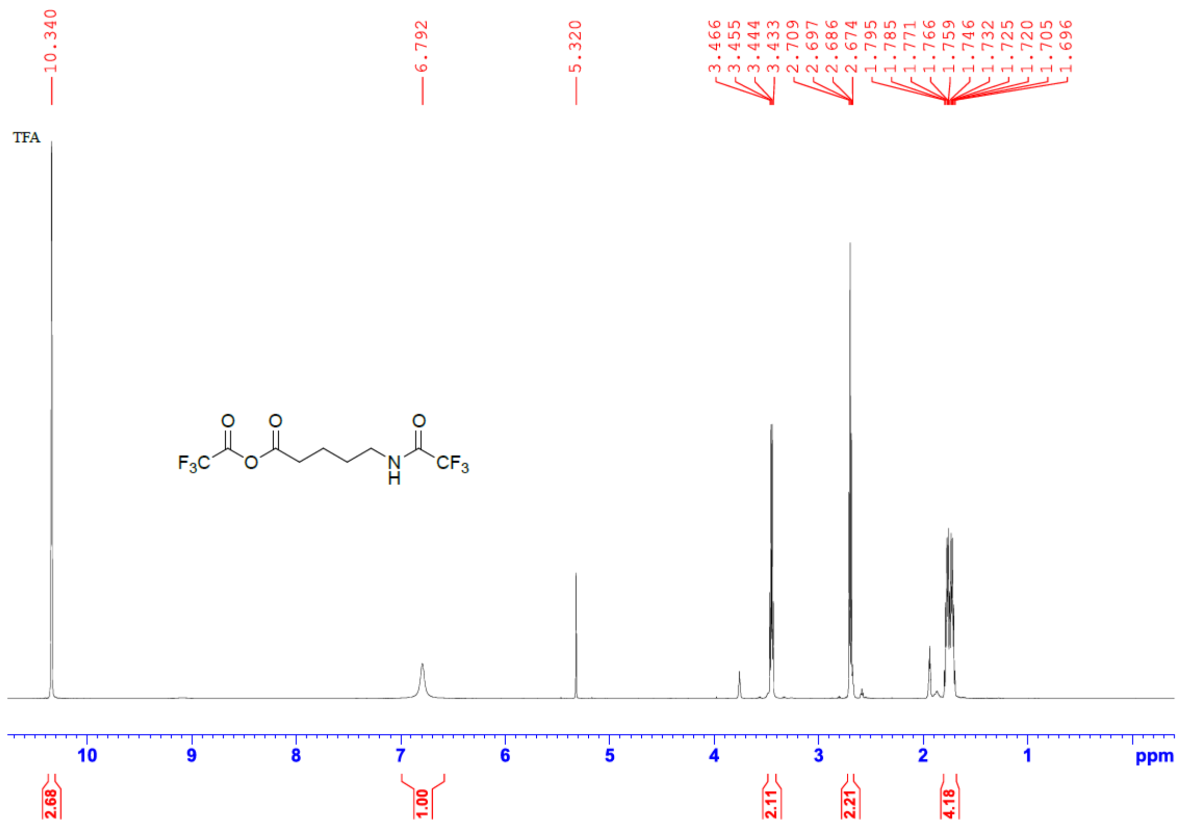

Figure S52. ^{1}H NMR spectrum of **8d** in CD_2Cl_2 (600 MHz).

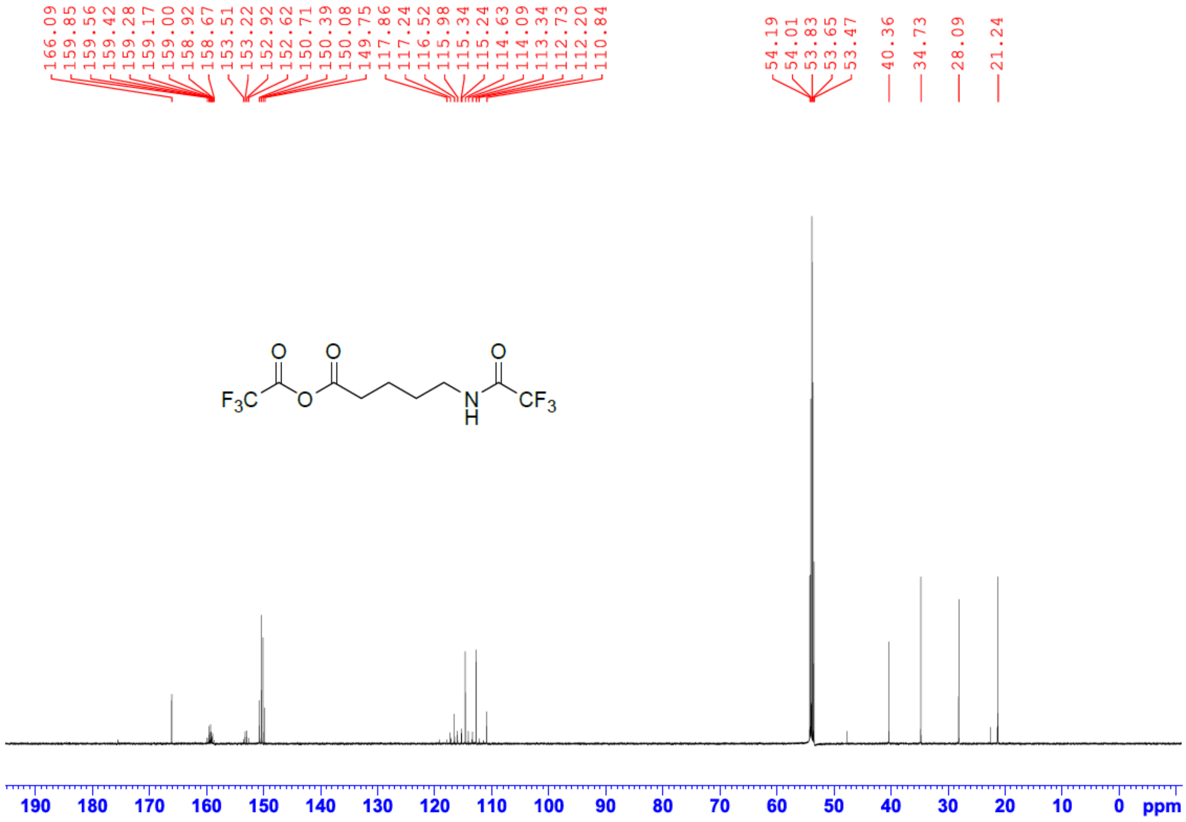

Figure S53. $^{13}C\{^{1}H\}$ NMR spectrum of **8d** in CD_2Cl_2 (151 MHz).

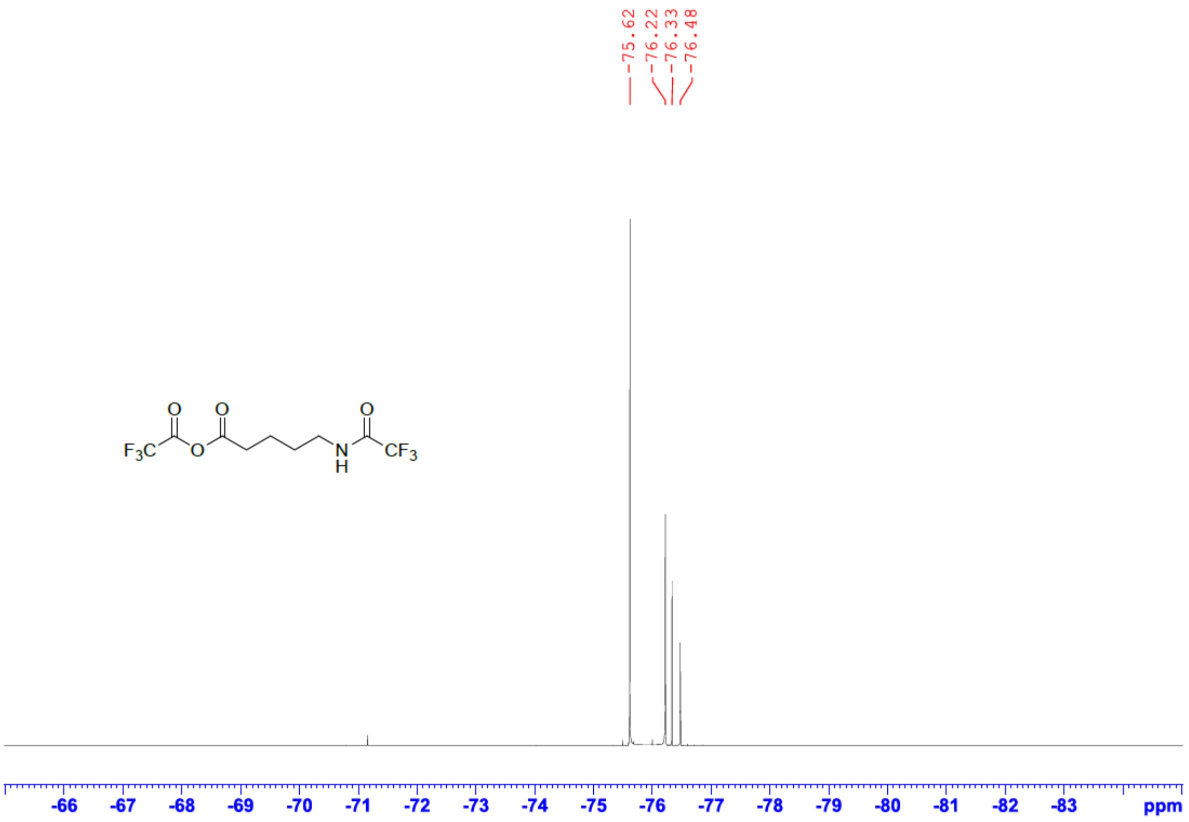

Figure S54. ^{19}F NMR spectrum of **8d** in CD_2Cl_2 (565 MHz).

Elemental Composition Report **Page 1**

Single Mass Analysis
Tolerance = 5.0 PPM / DBE: min = -1.5, max = 60.0
Element prediction: Off
Number of isotope peaks used for i-FIT = 9

Monoisotopic Mass, Even Electron Ions
118 formula(e) evaluated with 3 results within limits (all results (up to 1000) for each mass)
Elements Used:
C: 0-15 H: 0-25 N: 0-1 O: 0-5 F: 0-6

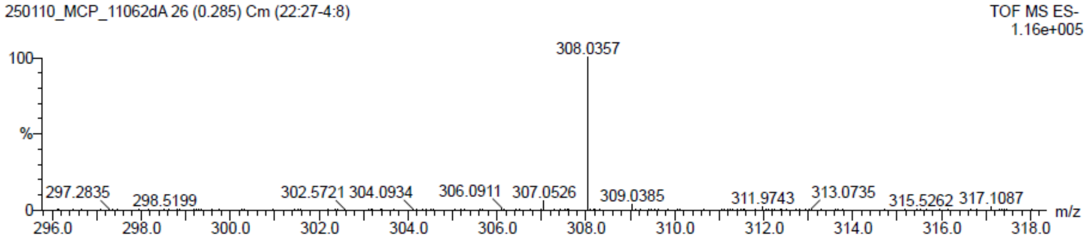

Minimum:				-1.5				
Maximum:		5.0	5.0	60.0				
Mass	Calc. Mass	mDa	PPM	DBE	i-FIT	Norm	Conf(%)	Formula
308.0357	308.0358	-0.1	-0.3	3.5	393.2	0.083	92.08	C9 H8 N O4 F6
	308.0346	1.1	3.6	7.5	395.9	2.693	6.77	C12 H7 N O3 F5
	308.0371	-1.4	-4.5	10.5	397.6	4.460	1.16	C14 H8 N O5 F2

Figure S55. HRMS spectrum of **8d**.

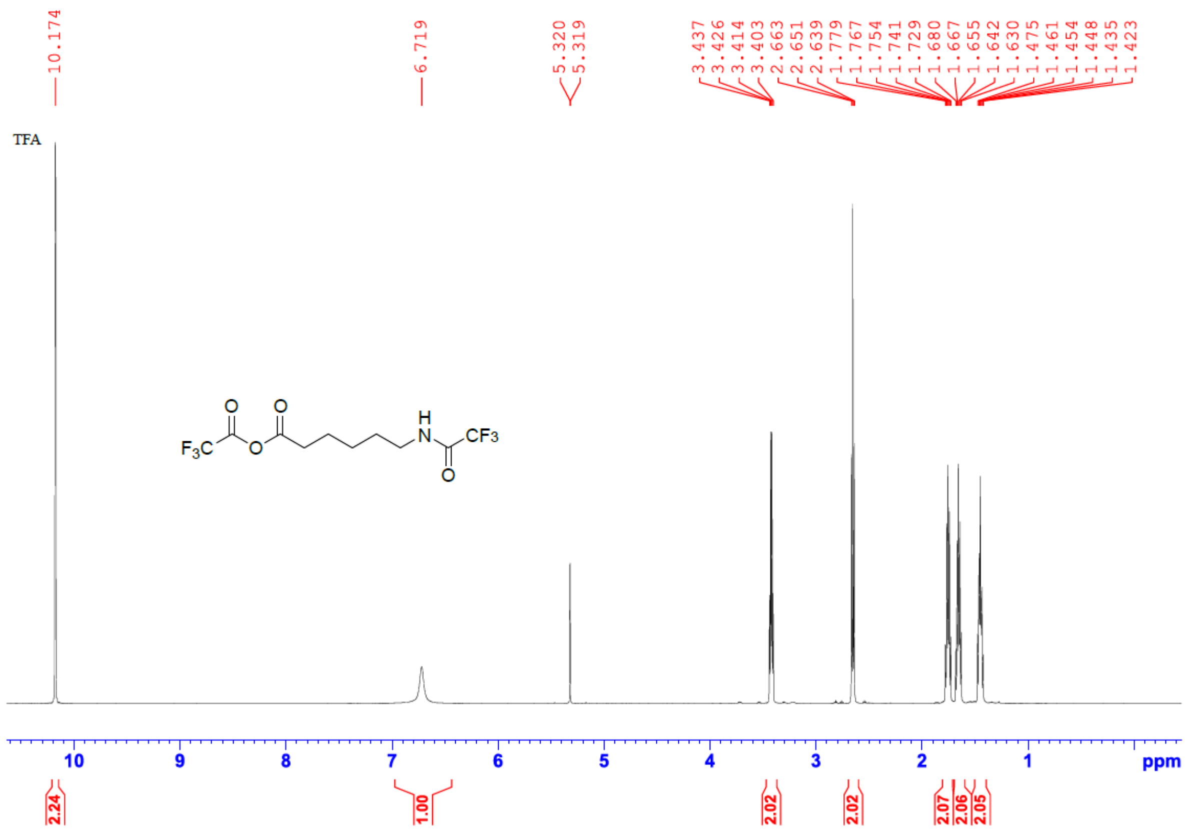

Figure S56. ^{1}H NMR spectrum of **8e** in CD_2Cl_2 (600 MHz).

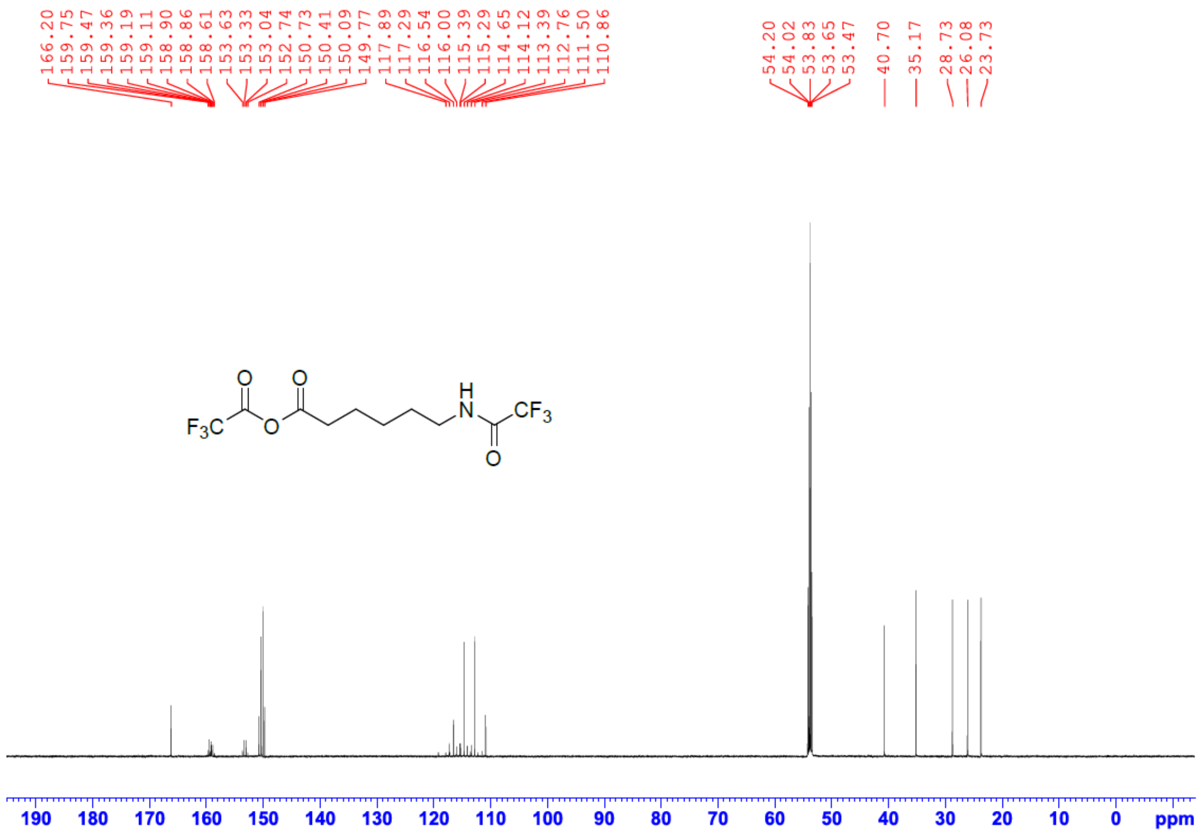

Figure S57. $^{13}C\{^{1}H\}$ NMR spectrum of **8e** in CD_2Cl_2 (151 MHz).

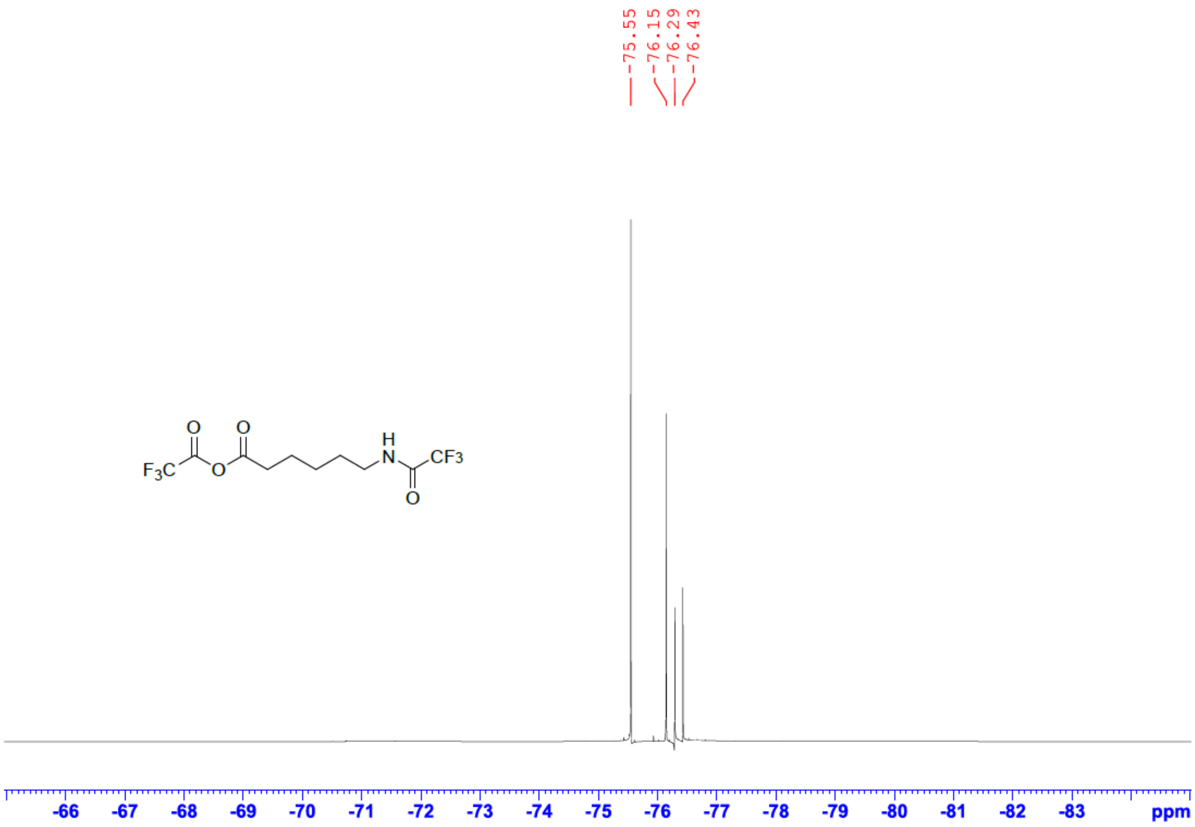

Figure S58. ^{19}F NMR spectrum of **8e** in CD_2Cl_2 (565 MHz).

Elemental Composition Report **Page 1**

Single Mass Analysis
Tolerance = 5.0 PPM / DBE: min = -1.5, max = 60.0
Element prediction: Off
Number of isotope peaks used for i-FIT = 9

Monoisotopic Mass, Even Electron Ions
105 formula(e) evaluated with 2 results within limits (all results (up to 1000) for each mass)
Elements Used:
C: 0-15 H: 0-25 N: 0-1 O: 0-5 F: 0-6

250110_MCP_11062eA 27 (0.294) Cm (27:30-13:15) TOF MS ES- 9.59e+004

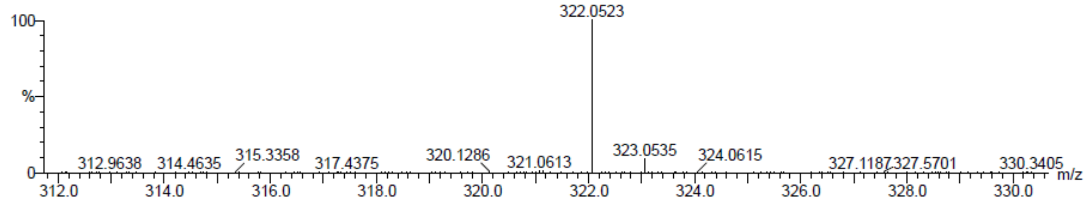

Minimum:				-1.5				
Maximum:		5.0	5.0	60.0				
Mass	Calc. Mass	mDa	PPM	DBE	i-FIT	Norm	Conf(%)	Formula
322.0523	322.0514	0.9	2.8	3.5	442.7	0.002	99.83	C10 H10 N O4 F6
	322.0527	-0.4	-1.2	10.5	449.0	6.351	0.17	C15 H10 N O5 F2

Figure S59. HRMS spectrum of **8e**.

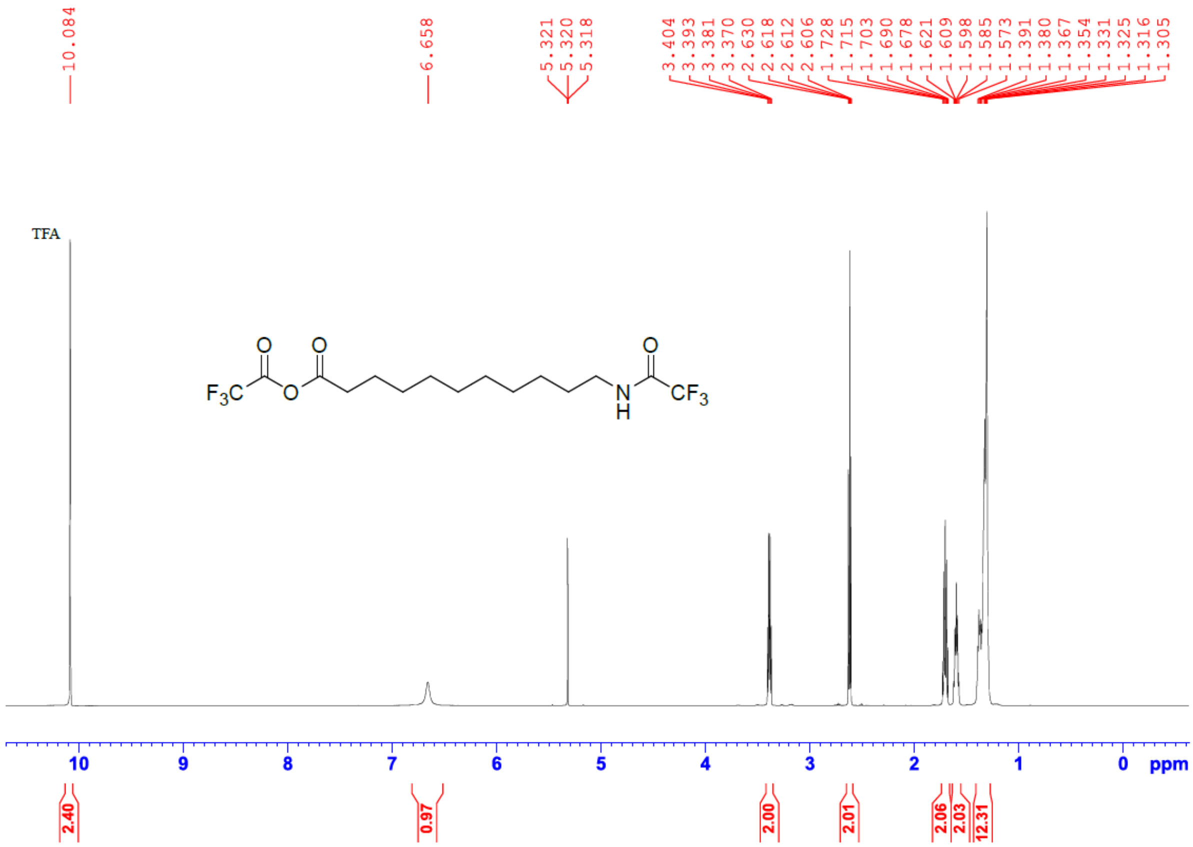
10.084
6.658
5.321
5.320
5.318
3.404
3.393
3.381
3.370
2.630
2.618
2.612
2.606
1.728
1.715
1.703
1.690
1.678
1.621
1.609
1.598
1.585
1.573
1.391
1.380
1.367
1.354
1.331
1.325
1.316
1.305
TFA
10 9 8 7 6 5 4 3 2 1 0 ppm
2.40
0.97
2.00
2.01
2.06
2.03
12.31

Figure S60. ^{1}H NMR spectrum of **8f** in CD_2Cl_2 (600 MHz).

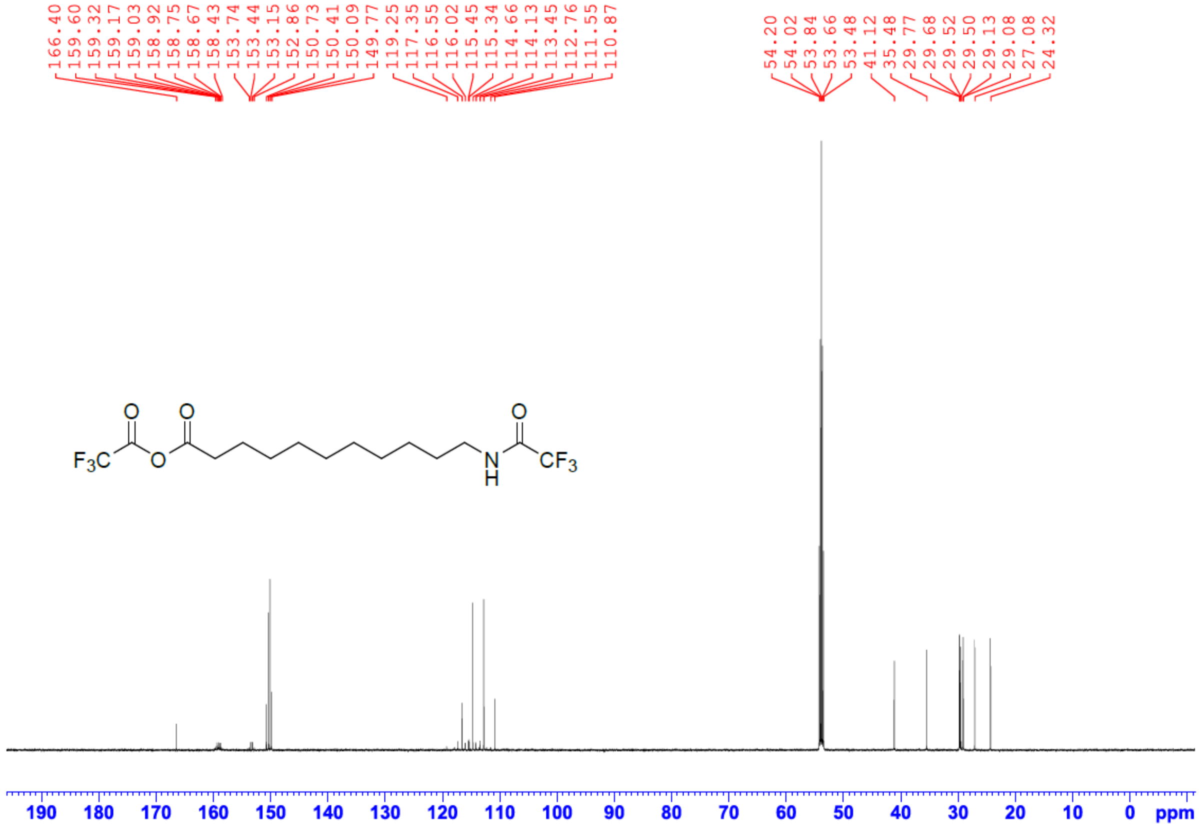

Figure S61. $^{13}C\{^{1}H\}$ NMR spectrum of **8f** in CD_2Cl_2 (151 MHz).

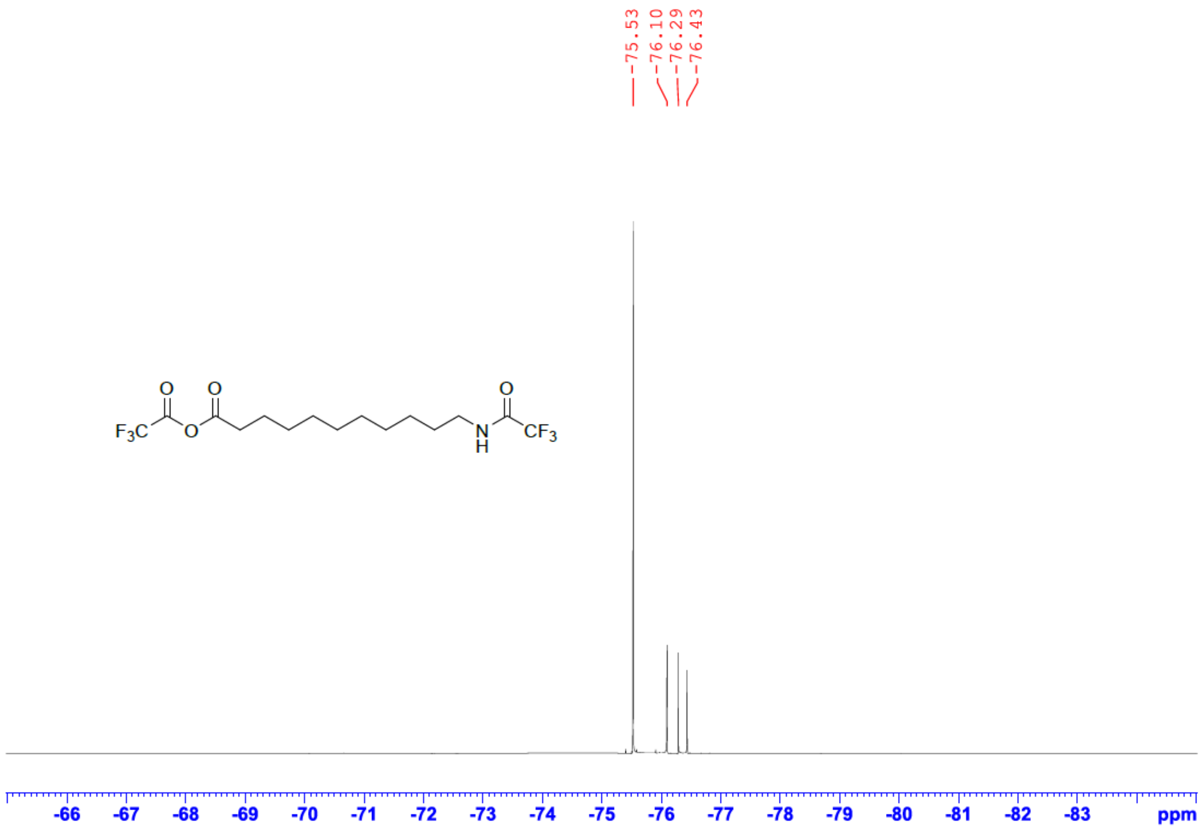

Figure S62. ^{19}F NMR spectrum of **8f** in CD_2Cl_2 (565 MHz).

Elemental Composition Report **Page 1**

Single Mass Analysis
Tolerance = 5.0 PPM / DBE: min = -1.5, max = 60.0
Element prediction: Off
Number of isotope peaks used for i-FIT = 9

Monoisotopic Mass, Even Electron Ions
107 formula(e) evaluated with 3 results within limits (all results (up to 1000) for each mass)
Elements Used:
C: 0-20 H: 0-30 N: 0-1 O: 0-5 F: 0-6

250110_MCP_11062fA 26 (0.285) Cm (26:32-60:64) TOF MS ES- 5.73e+005

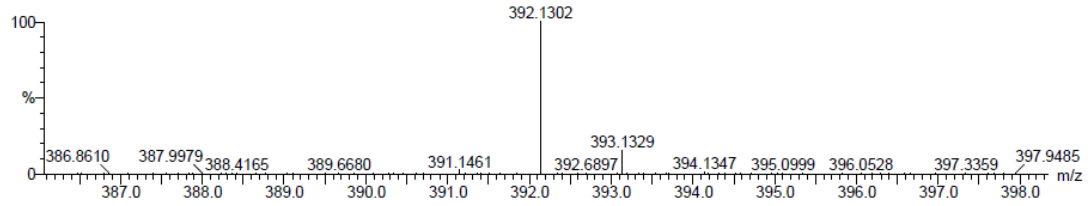

Minimum:				-1.5				
Maximum:		5.0	5.0	60.0				

Mass	Calc. Mass	mDa	PPM	DBE	i-FIT	Norm	Conf(%)	Formula
392.1302	392.1297	0.5	1.3	3.5	320.0	0.015	98.46	C15 H20 N O4 F6
	392.1285	1.7	4.3	7.5	324.2	4.197	1.50	C18 H19 N O3 F5
	392.1310	-0.8	-2.0	10.5	328.0	8.018	0.03	C20 H20 N O5 F2

Figure S63. HRMS spectrum of **8f**.

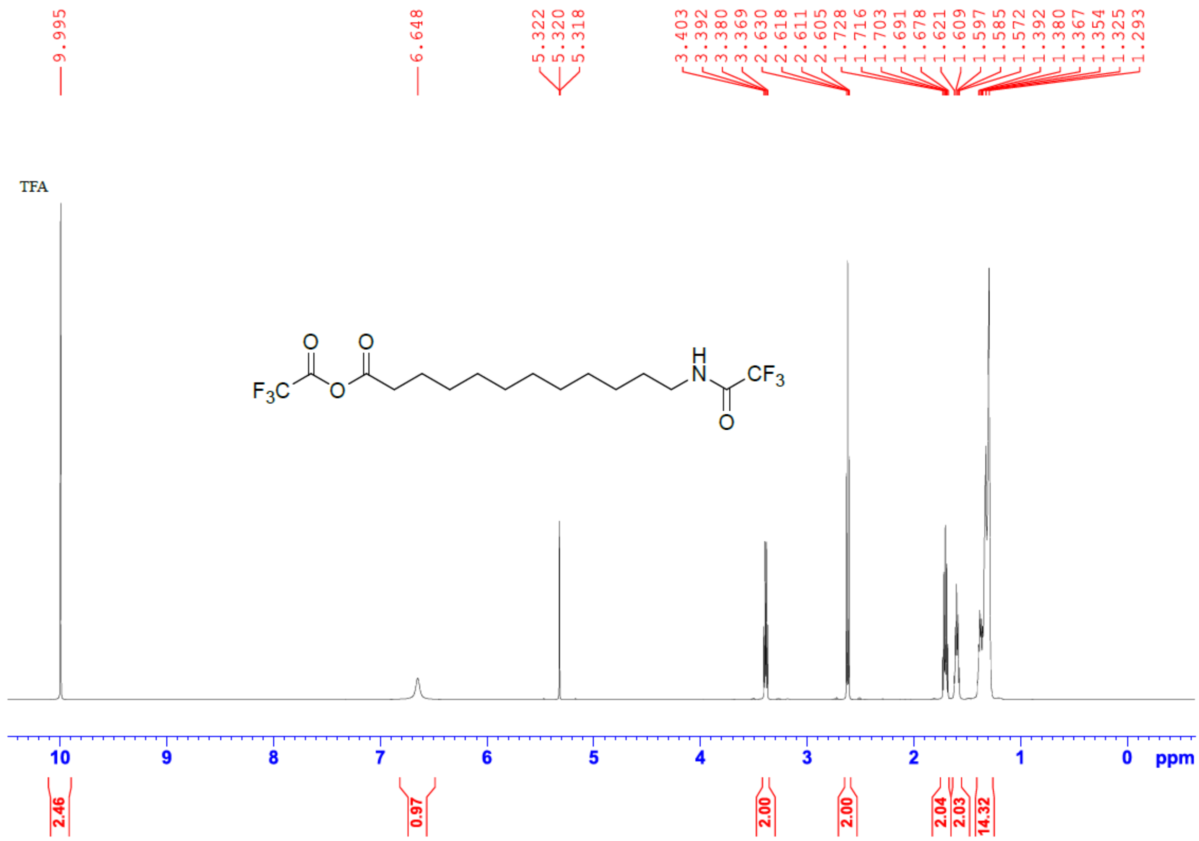

Figure S64. ^{1}H NMR spectrum of **8g** in CD_2Cl_2 (600 MHz).

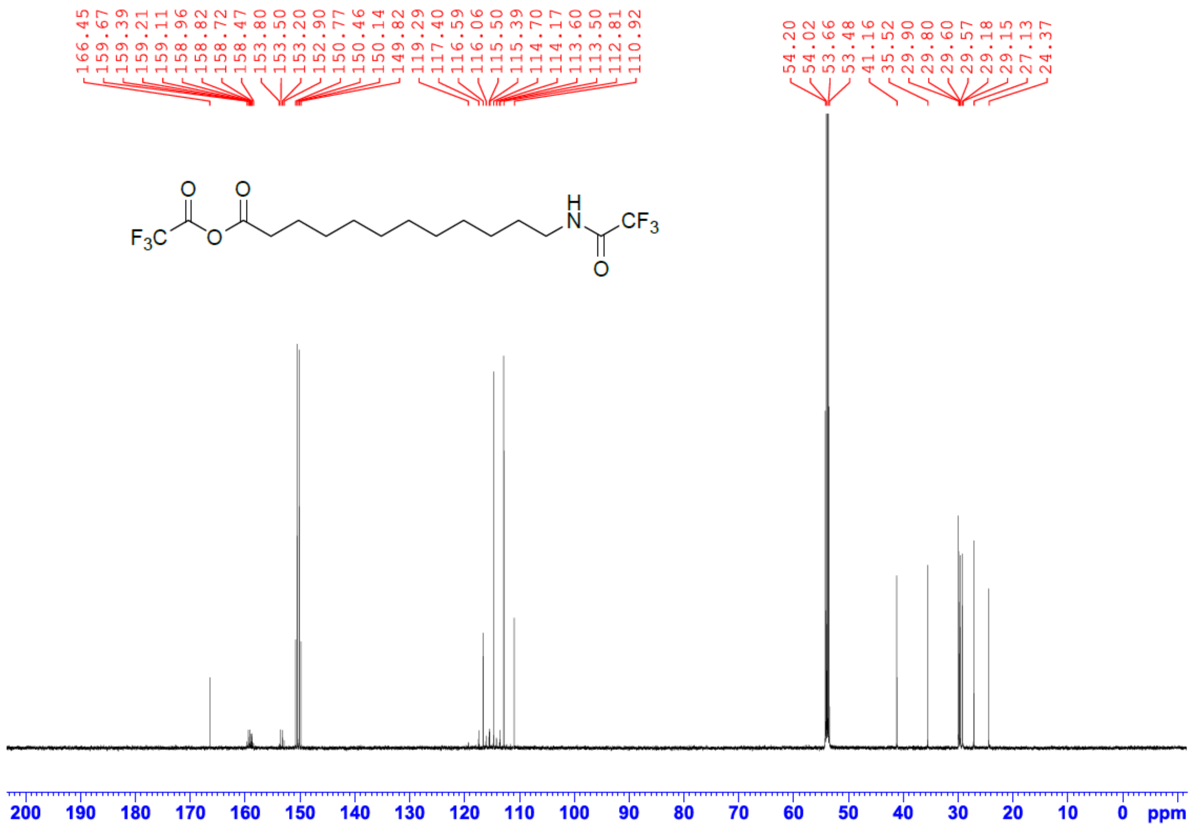

Figure S65. $^{13}C\{^{1}H\}$ NMR spectrum of **8g** in CD_2Cl_2 (151 MHz).

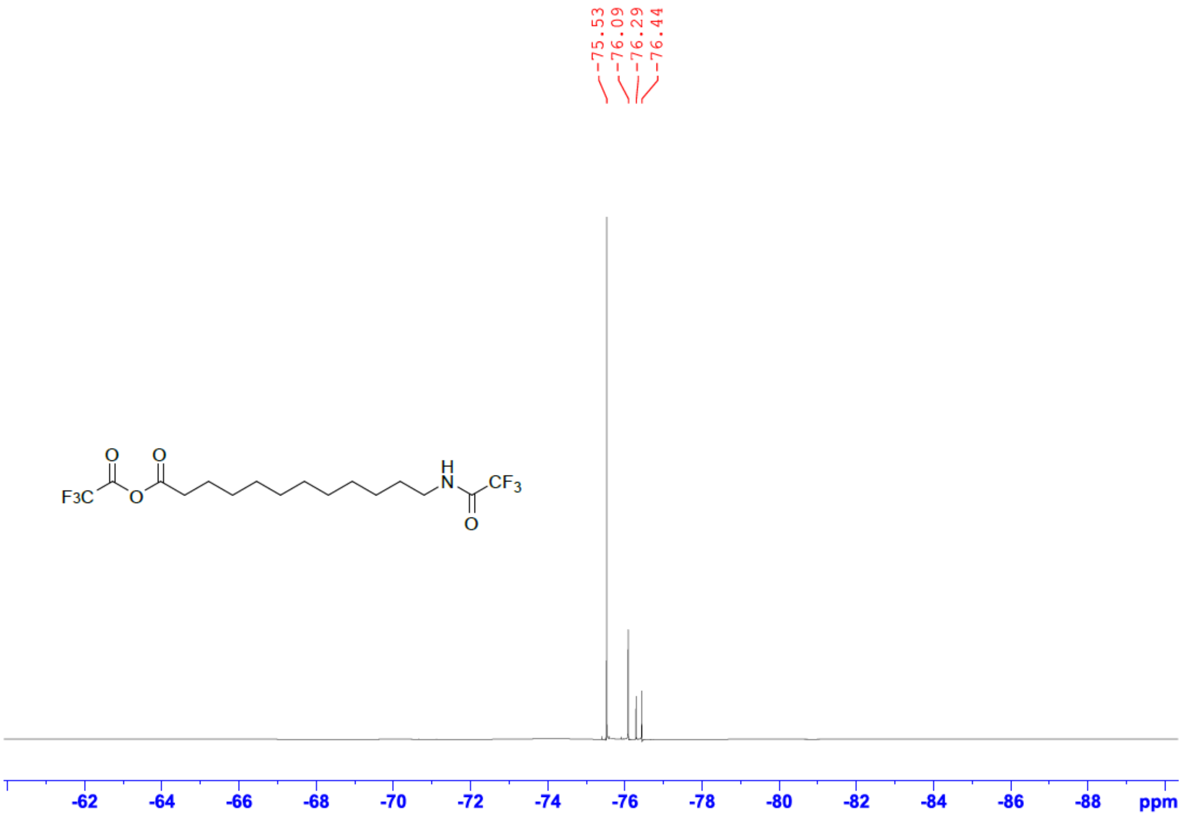

Figure S66. ^{19}F NMR spectrum of **8g** in CD_2Cl_2 (565 MHz).

Elemental Composition Report **Page 1**

Single Mass Analysis
Tolerance = 5.0 PPM / DBE: min = -1.5, max = 60.0
Element prediction: Off
Number of isotope peaks used for i-FIT = 9

Monoisotopic Mass, Even Electron Ions
96 formula(e) evaluated with 2 results within limits (all results (up to 1000) for each mass)
Elements Used:
C: 0-20 H: 0-30 N: 0-1 O: 0-5 F: 0-6

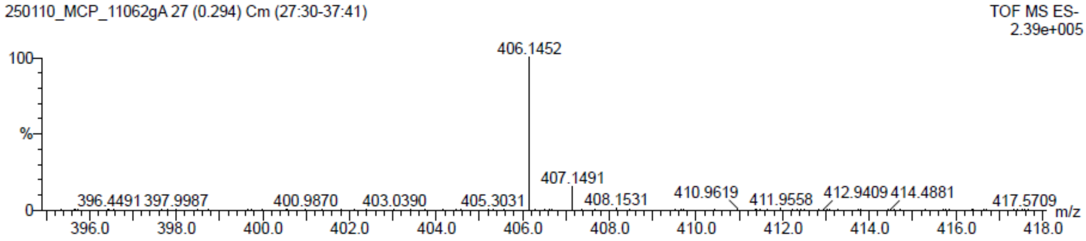

Minimum:				-1.5				
Maximum:		5.0	5.0	60.0				
Mass	Calc. Mass	mDa	PPM	DBE	i-FIT	Norm	Conf(%)	Formula
406.1452	406.1453	-0.1	-0.2	3.5	289.3	0.416	65.94	C16 H22 N O4 F6
	406.1442	1.0	2.5	7.5	290.0	1.077	34.06	C19 H21 N O3 F5

Figure S67. HRMS spectrum of **8g**.

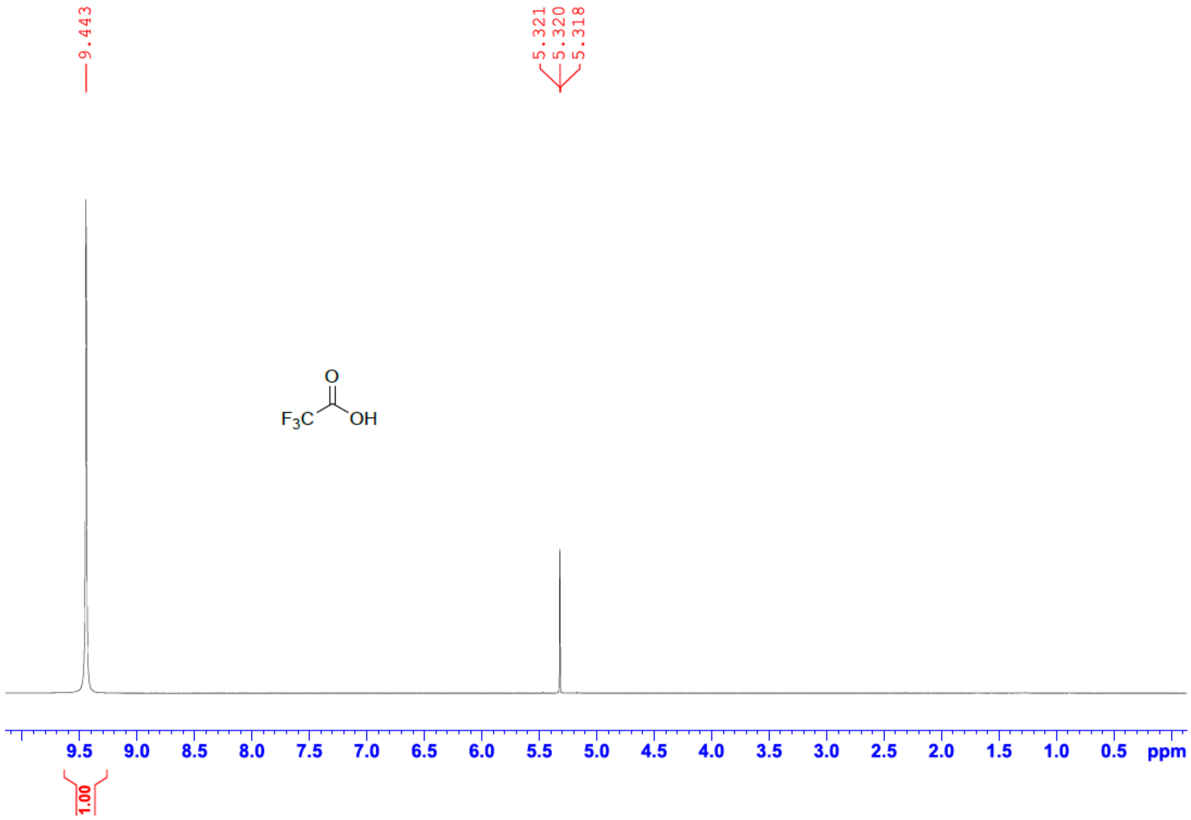

Figure S68. ^{1}H NMR spectrum of **trifluoroacetic acid** in CD_2Cl_2 (600 MHz).

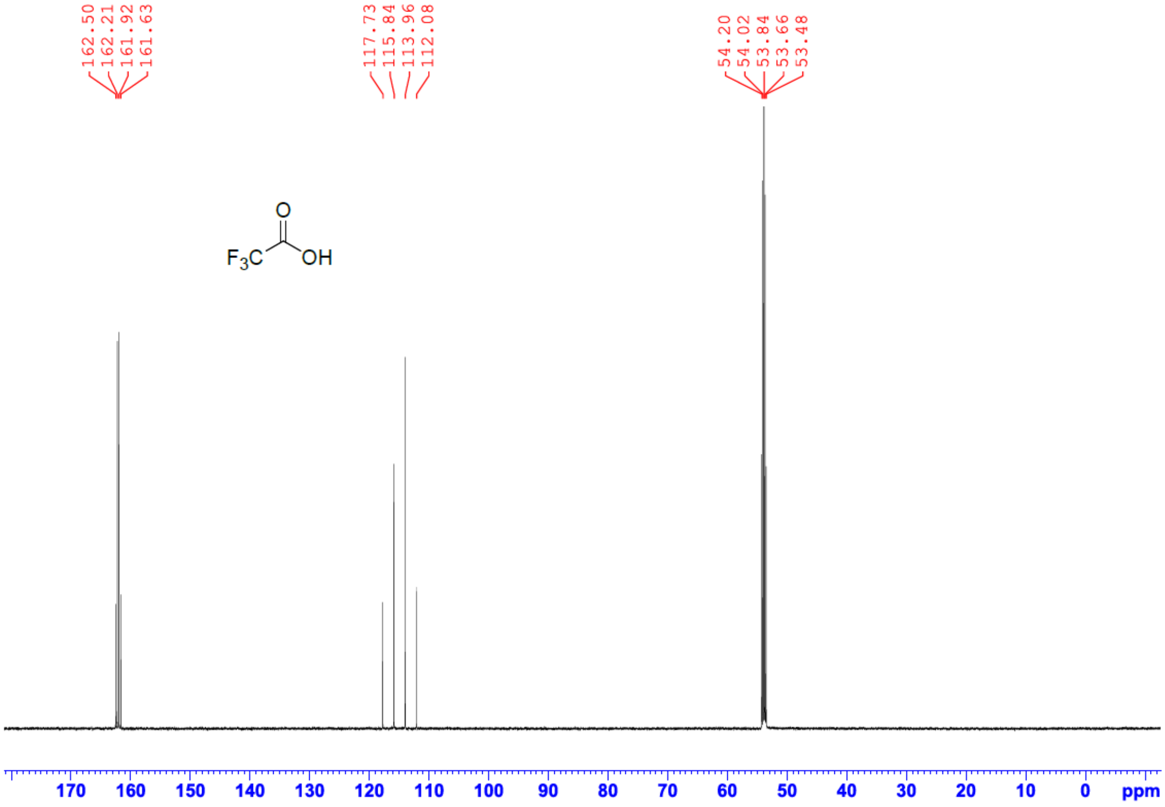

Figure S69. $^{13}C\{^{1}H\}$ NMR spectrum of **trifluoroacetic acid** in CD_2Cl_2 (151 MHz).

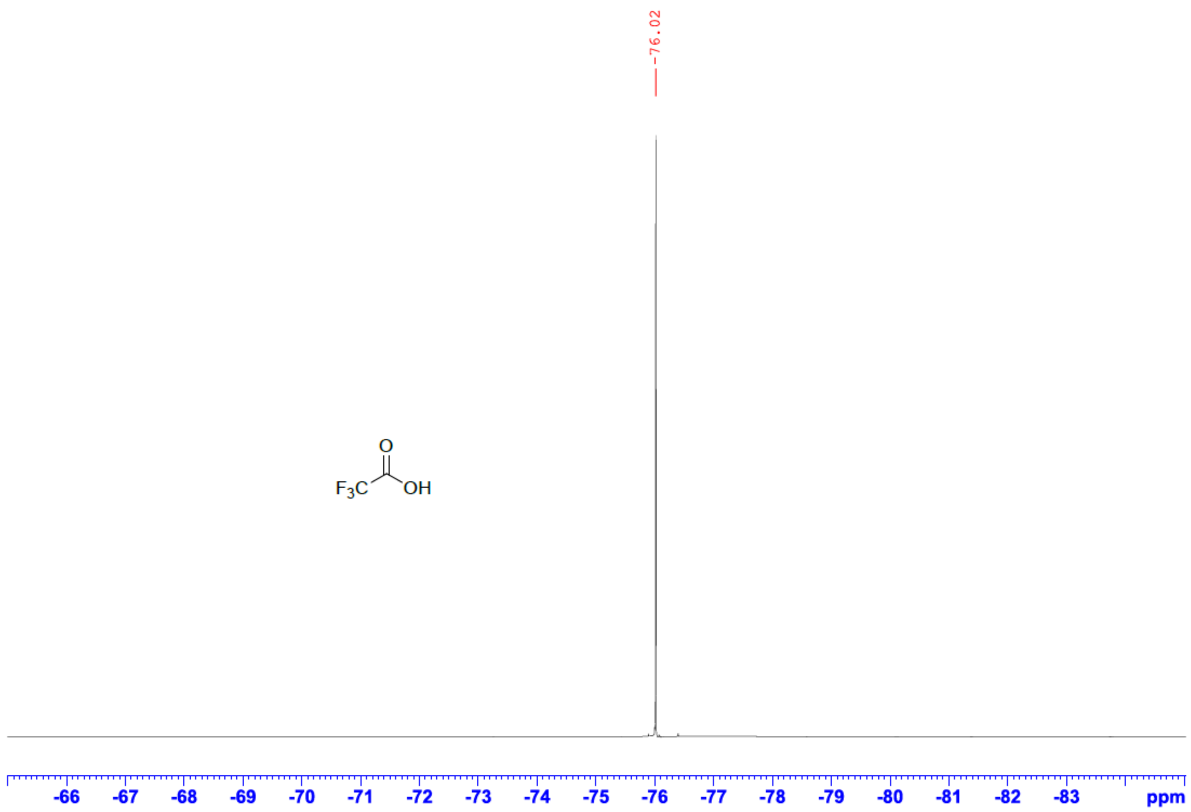

Figure S70. ^{19}F NMR spectrum of **trifluoroacetic acid** in CD_2Cl_2 (565 MHz).

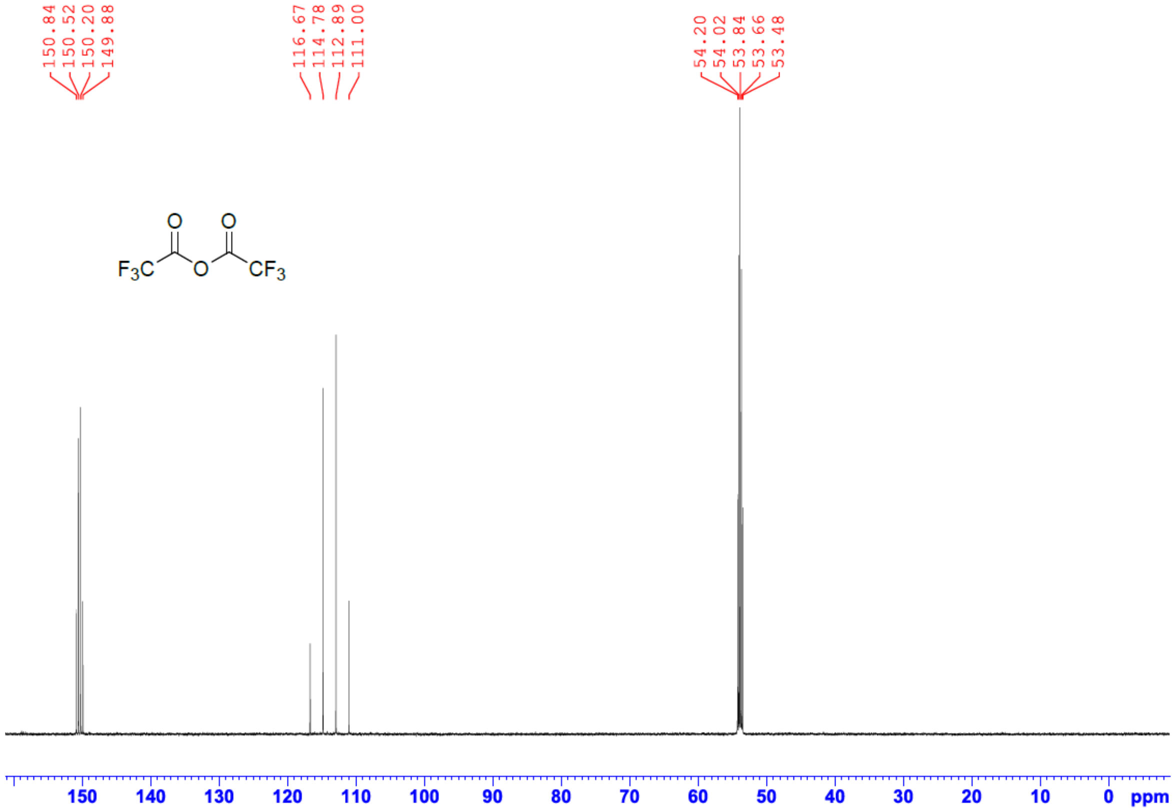

Figure S71. $^{13}C\{^{1}H\}$ NMR spectrum of **trifluoroacetic anhydride** in CD_2Cl_2 (151 MHz).

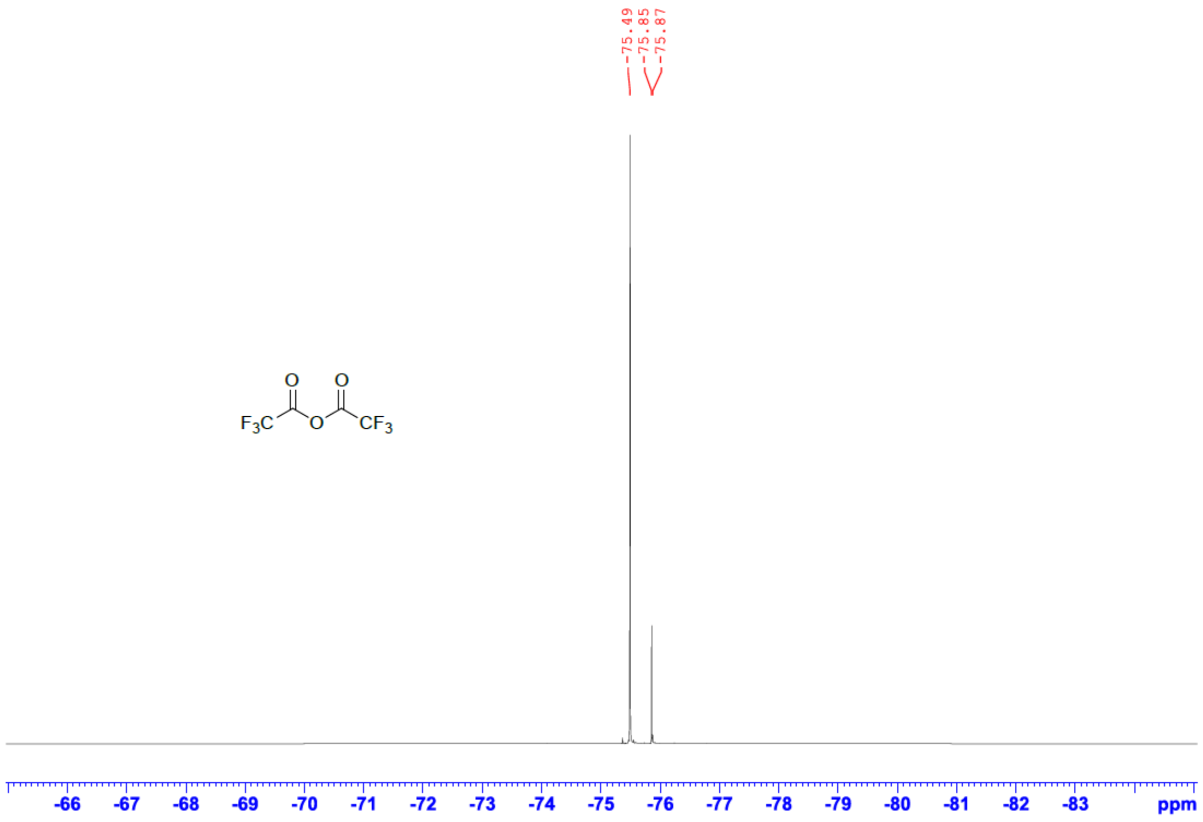

Figure S72. ^{19}F NMR spectrum of **trifluoroacetic anhydride** in CD_2Cl_2 (565 MHz).
